# Gut microbiomes of tribal communities in India vary with dairy and grain consumption

**DOI:** 10.1080/19490976.2026.2694242

**Published:** 2026-07-09

**Authors:** Emily R. Ebel, Abhijit Sanjiv Kulkarni, Dattatray S. Mongad, Matthew R. Olm, Sarangthem Indira Devi, Bilal Ahmad Mir, Shantanu Ozarkar, Erica D. Sonnenburg, Yogesh S. Shouche, Justin L. Sonnenburg, Dhiraj P. Dhotre

**Affiliations:** a Department of Microbiology and Immunology, Stanford University School of Medicine, Stanford, CA, USA; b BRIC - National Centre for Cell Science, Savitribai Phule Pune University, Pune, MH, India; c Symbiosis Centre for Climate Change and Sustainability, Symbiosis International (Deemed University), Pune, India; d Department of Computing, University of Turku, Turku, Finland; e Department of Integrative Physiology, University of Colorado Boulder, Boulder, CO, USA; f Department of Biotechnology, Institute for Bioresource and Sustainable Development, Imphal, MN, India; g Department of Botany, North Campus, University of Kashmir, Baramulla, J&K, India; h Department of Anthropology, Savitribai Phule Pune University, Pune, MH, India; i SKAN Research Trust, Bangalore, KA, India; j Chan Zuckerberg Biohub, San Francisco, CA, USA; k Center for Human Microbiome Studies, Stanford University School of Medicine, Stanford, CA, USA

## Abstract

Highly diverse gut microbiomes of non-industrialized populations share similarities with ancestral states of symbiosis and are linked to low rates of chronic inflammatory diseases. Yet there is still limited understanding of the diverse array of non-industrialized gut microbiomes throughout the world, including among the tribal populations of India. In this study, we surveyed dietary and fecal microbiome variation among 76 adults from eight tribal communities in four biogeographic regions of India, including Warli on the western coast, Gond and Madia in the northeast Deccan Plateau, Kabui (or Rongmei Naga) in the northeast hills of the Himalayas, and Balti, Boto, Brokpa, and Purigpa in the northwest Trans-Himalayas. Metagenomic and 16S sequencing of fecal samples identified *Segatella*, *Agathobacter*, and *Faecalibacterium* as core members of the gut microbiome of all populations, with *Segatella copri* (formerly *Prevotella copri*) dominant at mean 25%–47% relative abundance. Four Trans-Himalayan populations with diets uniquely defined by dairy and diverse cereals had elevated gut alpha diversity and distinct beta diversity, driven by prevalent and abundant *Bifidobacterium* as well as taxa shared with the ruminant microbiome. Strains of *B. adolescentis* present in the dairy-consuming populations were genetically distinct from industrialized strains around the world and encoded CAZymes consistent with selection by dairy and grain consumption. The gut microbiomes of a minority of subjects shared taxonomic and functional features with a previously described sample of Californians, suggesting that the pressures posed by globalization could be impacting the microbiomes of tribal populations. These results highlight the nutritional and microbiological contribution of dairy livestock in shaping gut communities and emphasize the large effect that lifestyle can have on the diversity and function of non-industrialized gut microbiomes.

## Introduction

The human gut microbiome plays numerous roles in health and disease.^1^ ^,^ ^2^ Global variation in the gut microbiome is strongly structured by lifestyle factors including diet, antibiotics, and exposure to environmental microbes.^3-7^ In particular, industrialized lifestyles are associated with large compositional and functional shifts along with reductions in microbiome diversity, which may drive immune dysregulation and contribute to various diseases.^8-10^ At the same time, dietary changes can have health-promoting effects on microbiome composition and function.^11-17^ For example, consuming fermented dairy products increases the relative abundance of *Bifidobacterium* and *Lactobacillus,* ^18^ commensal gut bacteria that have been associated with multiple dimensions of physical health.^19^ ^,^ ^20^ Host factors such as lactase persistence, which is uncommon in India, also shape the gut microbiome by enabling host absorption of carbohydrates that would otherwise reach the colon.^21^ ^,^ ^22^ A major open question is how dietary variation across cultures and geographies impacts functional variation in the gut microbiome.

The large country of India is home to thousands of ethnic groups and enormous dietary diversity,^23^ ^,^ ^24^ posing a unique opportunity to test the relationship between microbiome and diet. Although Indians make up nearly 18% of the global population, they have provided fewer than 1% of the samples used in human microbiome studies through 2022.^25^ The available data indicate that adult gut communities across India are distinct from those in other parts of the globe, including the United States, Europe, East Asia, and the Hadza hunter-gatherers of Tanzania.^26-30^ Indian gut microbiomes are dominated by *Segatella copri*, a subset of the genus previously known as *Prevotella,* ^31^ ^,^ ^32^ which is found at 30-60% relative abundance in tribal populations as well as urban centers.^26^ ^,^ ^33-37^ In Western populations, *S. copri* is a VANISH (Volatile and/or Associated Negatively with Industrialized Societies of Humans) taxon that is typically replaced by *Bacteroides* species with greater capacity to metabolize host mucus.^38-44^ Besides a few widespread taxa like *Segatella* and *Faecalibacterium*, the core microbiome of Indian adults varies with urbanization, ethnicity, and dietary practices. For example, tribal populations tend to have higher gut alpha diversity than urban dwellers.^37^ Populations that regularly consume dairy also have elevated levels of taxa found in fermented milk products, like *Lactobacillus* and *Catenibacterium*; or cultivated by dairy consumption, such as *Bifidobacterium.* ^27^ ^,^ ^29^ ^,^ ^45^ However, only a small fraction of the diverse tribal communities of India has been sampled for microbiome studies to date, and further work is needed to evaluate the link between dietary variation and the non-industrialized gut microbiome.^46^

**Figure 1.**
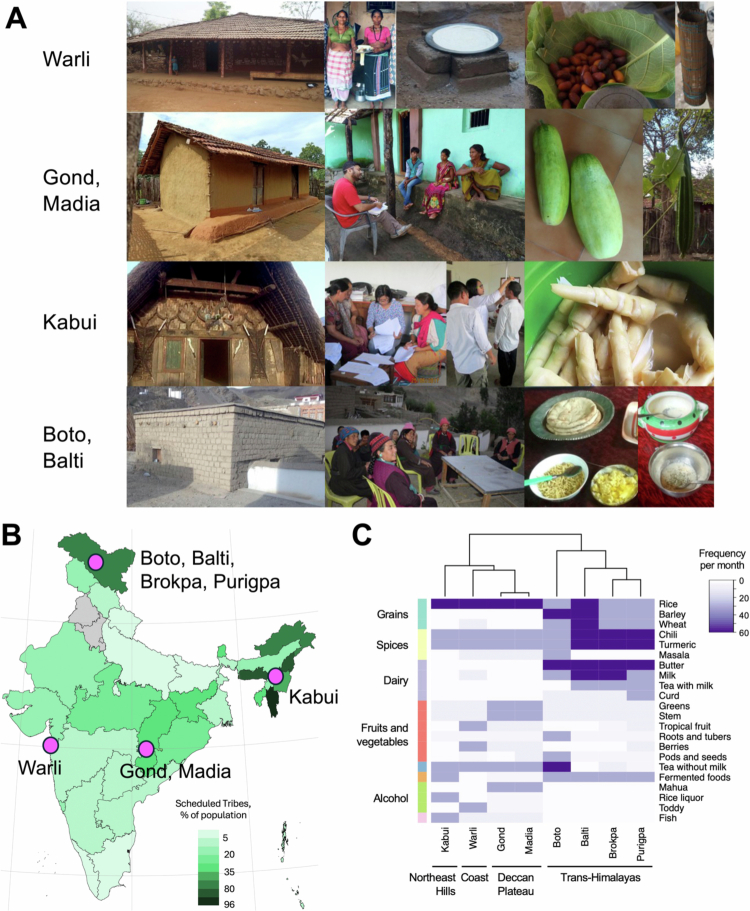
Geographic and dietary diversity of the eight sampled tribal populations in India. **A**, Photographs of traditional dwellings, residents, and unique foods in participating villages. **B**, Geographical locations of the eight sampled populations. Map is from Survey of India. Green shading indicates tribal populations as a percentage of state population in the 2011 Census of India. **C**, Food items consumed daily by at least one population. Populations are hierarchically clustered using food frequency data. Foods are ordered by total monthly consumption across all populations.

In this study we recruited members of eight tribal populations from four biogeographically distinct regions of India (Figure 1A,B). All participants contributed dietary data and fecal samples for 16S and metagenomic sequencing. Each community has a distinct identity, history, and shared culture and follows traditional dietary practices. The Warli live on the western coast of India and speak the native Warli dialect, which belongs to the Indo-European language family. The Kabui, also known as the Rongmei Naga, is a Tibeto-Burmese ethnic group found in the northeast Himalayan hills of the Indian subcontinent. The Gond and an associated group, Madia, speak the Madia language from the Dravidian language family and live in or near the forest in the northeast part of the Deccan plateau. The Brokpa community, which speaks an Indo-Aryan language, and the Purigpa, Balti, and Boto communities, who speak the Tibetan languages Purig, Balti, and Ladhakhi, respectively, live within a 50 km radius in the high-altitude cold desert of the Indian Trans-Himalayas. All eight communities practice a combination of small-scale agriculture and seasonal hunting and foraging: cereals, millets and leafy vegetables are mostly cultivated within a household, with fruits mostly foraged from the landscape and animals either hunted or raised domestically. With the exception of the Gond, whose gut microbiomes have been previously profiled with 16S sequencing,^46^ the sampled populations have never before participated in microbiome studies. Together, these eight communities represent a diversity of Indian geographies, ancestries, and cultures that offer a unique opportunity to examine links between diet, lifestyle, and the gut microbiome.

## Results

### Regional variation in community diets including dairy, grains, and alcohol

From June to October 2019 (summer season), we recruited a total of 76 healthy adults from eight tribal populations located in four different biogeographic regions of India (Figure 1A,B; Supplementary File 4). Fecal samples were collected with approval from the NCCS Institute Ethics Committee and the verbal and written consent of all participants (see **Methods**). Study participants had a mean age of 34 and were 58% female (Table S1). Participant body mass index (BMI) appeared higher among the Trans-Himalayan and Kabui populations (mean 24.2) than the Warli, Gond, and Madia (mean 19.5, *p* = 2.4 × 10^−6^, t-test), with the caveat that not all participants consented to analysis of their metadata (Table S1). For each population, we established an average dietary profile using 24-hour dietary recalls and seasonal food frequency questionnaires completed by each participant (Supplementary Files 1 and 2). The data indicated that rice was a staple cereal and the spices turmeric and chili were consumed daily by all eight populations (Figure 1C; Figure 1 Supplement 1). Most daily diets also included tea, cooking oil, and 2-4 instances of a variety of foraged fruits and homegrown vegetables. Plant-based foods made up the majority of community diets in the sampled summer season, with meat or fish included twice weekly on average. All populations except Warli consumed native fermented foods at least weekly, although the specific food fermented ranged from grain flour to fish or pork to milk in the form of curd/buttermilk. Processed table sugar and fatty street foods were rarely or never consumed, consistent with the non-industrialized lifestyles of all eight populations.

Hierarchical clustering of the food frequency data revealed that the four populations from the Trans-Himalayan region shared similar diets, in contrast to the populations from the three other regions (Figure 1C; Figure 1 Supplement 1). Barley, wheat, milk, and homemade butter were consumed daily by the Purigpa, Boto, Brokpa, and Balti, but were absent or rare in the diets of the Gond, Madia, Kabui, and Warli. The Purigpa regularly consumed fermented milk in the form of curd (or buttermilk), while the other Trans-Himalayan populations primarily consumed unfermented milk and butter. The daily diets of the Gond, Madia, Kabui, and Warli also included traditional fermented alcoholic beverages brewed over short periods within households, which were not part of the living tradition of the Trans-Himalayan populations. Additional dietary differences included higher consumption of fish by the Kabui; fruits by the Warli; and greens and stems by the Gond and Madia. Overall, while the diet of each individual population was unique, the inclusion of dairy products and a variety of grains and the exclusion of alcohol were major factors distinguishing the Trans-Himalayan populations from those in other regions.

### Elevated gut taxonomic diversity in Trans-Himalayan populations

We next examined whether gut microbiome diversity varied among populations and biogeographic regions. Using 16S rRNA gene amplicon sequencing and DADA2 analysis,^47^ we detected a total of 1,005 ribosomal sequence variants (RSVs) belonging to 166 genera from an average of 50,282 filtered reads per sample. We also performed metagenomic sequencing at a mean depth of 1.46 Gb per sample and used inStrain^48^ to identify 500 species-representative genomes (SRGs) belonging to 203 genera from the eight populations combined. Two hundred and twenty of these SRGs were metagenome-assembled genomes (MAGs) assembled in this study, and fourteen represented putatively novel species, including five in the genus *Collinsella* and three in the genus *UBA10281* (family: Borkfalkiaceae) (Table S2). Despite variation in sequencing depth among samples (Figure 2 Supplement 1A,B), relative abundances for common taxa derived from 16S and metagenomics were strongly correlated in all populations (Figure 2 Supplement 2, R^2^ > 0.85, *p* < 2 × 10^−5^, linear models).

**Figure 2.**
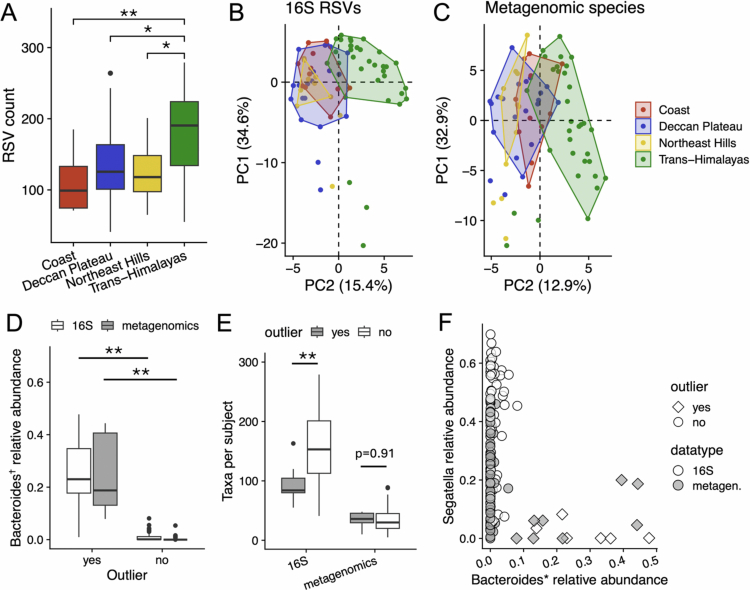
Gut microbiome diversity is elevated in Trans-Himalayan populations and depleted in outlier samples dominated by *Bacteroides.* **A**, Number of ribosomal sequence variants (RSVs) detected per sample varies by region (ANOVA *p* = 0.001) and is highest in the Trans-Himalayan samples. Asterisks represent results of Tukey’s Honest Significant Differences test. **B,C**, PCA of relative abundances of 16S RSVs and metagenomic species. Each point represents the microbiome of one participant. Outlier samples, defined by position on PCA as well as abundance of *Bacteroides* ^†^, are excluded from shaded hulls. **D**, Relative abundance of industrialization-associated *Bacteroides* ^†^ is elevated in outlier samples **E**, Outlier samples have lower alpha diversity in the higher-resolution 16S dataset. **F**, *Bacteroides* ^†^ relative abundance is negatively associated with *Segatella* relative abundance. **p < 0.01, *p < 0.05, t-tests. ^†^Quantification of *Bacteroides* in this figure includes *Phocaeicola* and *Parabacteroides.* These genera are presented separately in Figure 2 Supplements 4-5.

In the 16S data, rarefaction curves showed near-saturation of within-sample taxonomic diversity (Figure 2 Supplement 1D). The number of 16S RSVs per sample was higher in the Trans-Himalayan region than the other three regions (Figure 2A, ANOVA *p* = 0.001, pairwise Tukey’s HSD *p* ≤ 0.05), a pattern driven by all four Trans-Himalayan populations (Figure 2 Supplement 3A). All Trans-Himalayan villages sampled in this study were also located more closely to towns (<25 km) than the villages from other regions (25-50 km), suggesting that isolation by distance from denser settlements was not a major driver of variation in alpha diversity among populations.

Principal component analysis (PCA) of the relative abundances of 16S RSVs revealed that gut beta-diversity was significantly associated with region (Figure 2B, R^2^ = 0.16, *p* = 1 × 10^−5^, PERMANOVA) and population (Figure 2 Supplement 3B, R^2^ = 0.22, *p* = 1 × 10^−5^, PERMANOVA). PC2 strongly separated samples from the Trans-Himalayan region from samples from the Coast, Deccan Plateau, and Northeast Hills regions, which overlapped. A similar pattern was evident in PCA of the relative abundances of metagenomic species (Figure 2C, region R^2^ = 0.13, *p* = 1 × 10^−4^; Figure 2 Supplement 3E, population R^2^ = 0.19, *p* = 1 × 10^−5^, PERMANOVA), with PC2 again separating the Trans-Himalayan samples from other geographic regions. Beta-diversity was more weakly associated with population among the Trans-Himalayan communities only, especially using species-level metagenomic data (Figure 2 Supplement 3CF, 16S R^2^ = 0.13, *p* = 0.02; metagenomics R^2^ = 0.12, *p* = 0.19, PERMANOVA). Together, these results indicate that individuals from the Trans-Himalayan populations harbor a unique microbiome composition in addition to elevated alpha diversity. The relative similarity of the microbiomes of populations from other regions, despite the large distances between them, suggests that geographic separation alone is likely not the driving factor of the distinct aspects of the Trans-Himalayan microbiomes.

Notably, PC1 for both data types appeared to separate several putative outliers belonging to five populations from the majority of samples (Figure 2B,C). Upon inspection of stacked bar plots (Figure 2 Supplement 4 and Figure 2 Supplement 5), we found that these outlier samples harbored very high relative abundances of *Bacteroides* (Figure 2D) and/or *Parabacteroides* and *Phocaeicola*, which were formerly classified as *Bacteroides.* ^49^ ^,^ ^50^ These outlier samples are of interest because *Bacteroides* is typically found at lower abundance in the higher-diversity gut microbiomes of non-industrialized populations, while *Segatella* and other VANISH taxa show the opposite trend.^10^ In our dataset, we observed significant negative correlations between the relative abundances of *Segatella* and *Bacteroides* within samples (Figure 2F, 16S *p* = 4.2 × 10^–6^, metagenomic *p* = 0.006, linear models), suggesting that the gut microbiome composition of the outliers shared features with an industrialized profile. Consistent with this hypothesis, outlier status was negatively associated with alpha diversity in the higher-resolution 16S data (Figure 2E, *p* = 0.002, t-test). We could not identify any dietary patterns or available metadata that distinguished the eight high-*Bacteroides* outliers from others in their populations, nor could we fully rule out the possibility of contamination. We therefore chose to exclude the high-*Bacteroides* outlier samples from subsequent analysis of these traditional Indian microbiomes, except for a final comparison with industrialized Californians.

### Core microbiomes feature *Segatella* and regional diversity

**Figure 3.**
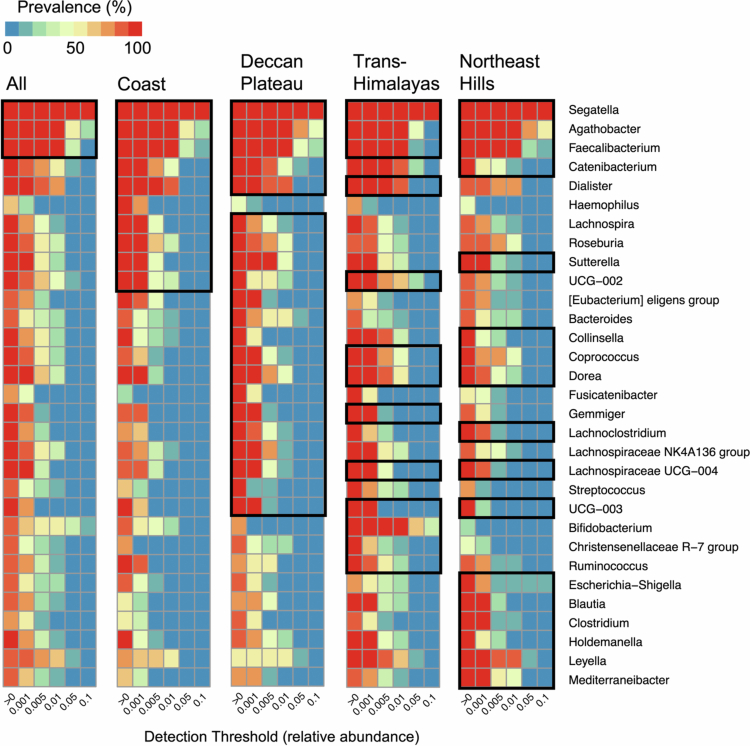
*Segatella, Agathobacter*, and *Faecalibacterium* comprise the core microbiome of all eight populations. The core microbiome is defined by genera detected with 16S sequencing in 100% of a set of samples, regardless of relative abundance. Core genera are outlined in black boxes for all eight populations combined (left) and each region individually.

The taxonomic composition of any individual human gut microbiome forms a unique microbial “fingerprint” that is largely stable through adulthood.^51^ Despite this individuality, a core microbiome of taxa found at very high prevalence across individuals is thought to provide essential, health-promoting functions to the human host.^52^ ^,^ ^53^ Similarly, genetic drift or selective forces within a cohort (e.g., dietary, environmental, medical) may give rise to a population-specific core microbiome. Here, we employed the most-widely used definition of the core microbiome^52^ to identify the set of genera detected with 16S sequencing in 100% of non-outlier samples for each region and the entire dataset (Figure 3).

The core microbiome of all eight populations comprised *Segatella*, *Agathobacter* (formerly *Eubacterium rectale,* ^54^ and *Faecalibacterium* at mean relative abundances of 47.0%, 7.1%, and 4.9%, respectively (Figure 3). Highly abundant gut *Segatella* has been observed across many Indian populations irrespective of urbanization,^27^ ^,^ ^33-37^ ^,^ ^55^ likely related to additional factors such as plant-based diet.^56^ For example, healthy Indian subjects from the large city of Ahmedabad^30^ have a similarly high abundance of the family Prevotellaceae as participants in this study (Figure 3 Supplement 1). *Eubacterium* is also prevalent across India,^27^ ^,^ ^34^ ^,^ ^55^ while *Agathobacter* is enriched in Indian versus Danish volunteers.^57^ The third core taxon, *Faecalibacterium*, is among the most prevalent and abundant bacteria found in the intestines of healthy adults worldwide,^58^ including in Indian populations.^55^ Overall, the 16S core microbiome shared by the eight populations shares major features with previously studied Indian populations.

As the dominant member of the Indian gut microbiome, *Segatella* contains substantial genetic diversity that cannot be profiled with 16S sequencing alone. In our metagenomic dataset, nine species-representative genomes (SRGs) were classified as *Segatella copri* (see **Methods**), consistent with a recent study that identified multiple distinct species within the *Segatella copri* complex.^31^ Seven of these *S. copri* SRGs were MAGs assembled in this study, indicating unique genetic variation in *S. copri* from the sampled populations compared to existing bacterial genomes in the Unified Human Gastrointestinal Genome (UHGG) Collection.^59^ Several additional MAGs were SRGs for other taxa formerly known as *Prevotella*, including *Leyella* (eight MAGs, mean relative abundance 3.9%), *Hallella* (two MAGs, mean relative abundance 1.4%), and *Segatella hominis* (one MAG, mean relative abundance 1.5%). These results highlight the unique genetic diversity present in Indian gut microbiomes that has been undersampled in global studies to date.

**Figure 4.**
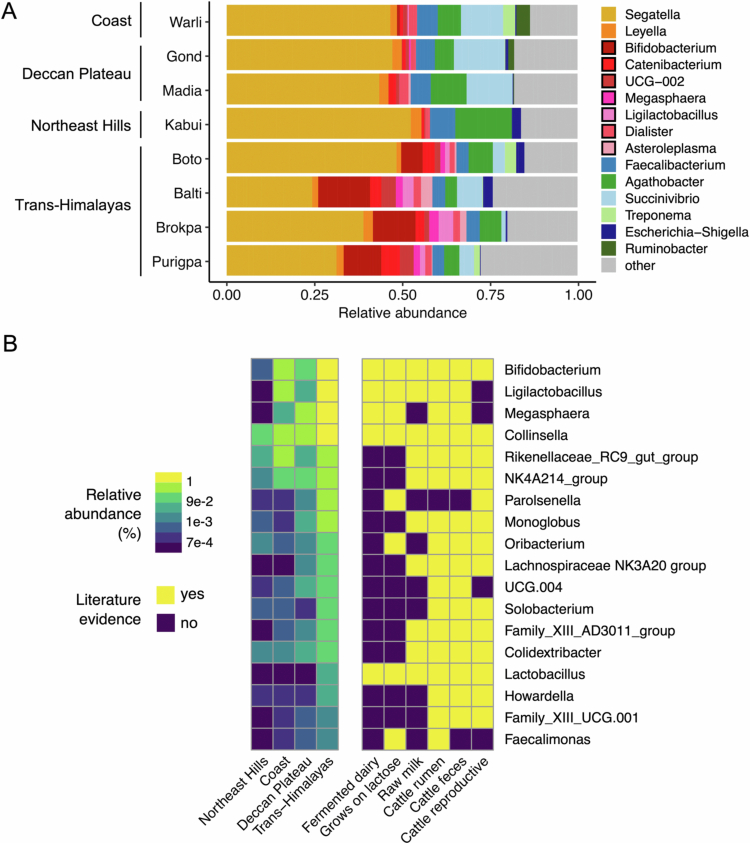
Trans-Himalayan gut microbiomes are enriched for taxa associated with cattle and fermented dairy products. **A**, Stacked bar plot of the average relative abundance per population of the 15 most abundant 16S genera. Rarer taxa are grouped as ‘other.’ Taxa colored red/pink with black borders in the legend are significantly more abundant (q < 0.05, Maaslin2) in the Trans-Himalayan populations than at least one other region. **B**, 16S genus-level taxa found at significantly higher relative abundance (q < 0.05, Maaslin2) in the Trans-Himalayan samples than all three other regions. Taxa are ordered by mean relative abundance in the Trans-Himalayas. Left panel: mean relative abundance by region. Right panel: Literature associations with dairy fermentation and cattle (Table S3).

The core microbiome defined by 16S for each region ranged in size from 10 to 21 genera, suggesting that taxonomic diversity across regions drives the smaller size of the overall core microbiome (Figure 3). *Catenibacterium*, *Dialister*, *Sutterella*, UCG-002, *Coprococus*, *Dorea*, Lachnospiraceae UCG-004, and UCG-003 were core genera in three regions; and *Lachnospira*, *Roseburia*, *Collinsella*, *Gemmiger*, and *Lachnoclostridium* were core genera in two regions. In general, taxa core to any one region were also found at high prevalence in other regions (Figure 3). However, *Bifidobacterium* was a notable exception to this pattern, occurring at 100% prevalence and mean 11.2% relative abundance in the Trans-Himalayan populations but 67% prevalence and mean 0.3% relative abundance in other regions (Figures 3 and 4). *Bifidobacterium* spp. are common residents of human gut microbiomes that are widely considered health-promoting and are often added to yogurt or sold commercially as probiotic supplements.^60^ Our dietary data show that the Trans-Himalayan populations are unique in this study for their frequent consumption of milk and more diverse cereals (Figure 1C), both of which have been associated with *Bifidobacterium.* ^61-63^ The occurrence of *Bifidobacterium* exclusively in the core microbiome of the Trans-Himalayan populations suggests the possibility that their unique diet and lifestyle might promote bacteria specialized in dietary carbohydrates that contribute to gut diversity and health.

### Trans-Himalayan gut microbiomes are enriched for bacteria associated with cattle and fermented dairy

We identified additional bacterial taxa contributing to differences in microbiome diversity across populations using linear models implemented in Maaslin2.^64^ In the 16S data, 58 genera differed significantly in abundance between the Trans-Himalayas and at least one other region (q ≤ 0.05, Figure 4 Supplement 1; Figure 4A, red/pink). The majority of these taxa (42/58) were significantly more abundant in the Trans-Himalayas, and eighteen were significantly more abundant in the Trans-Himalayas than all three other regions. Highly enriched and abundant genera in the Trans-Himalayas included *Bifidobacterium* and *Ligilactobacillus* (formerly *Lactobacillus* ^65^ (Figure 4; Figure 4 Supplement 2A), which are known for their ability to ferment lactose and/or respond to dairy in the human diet.^66^ ^,^ ^67^ Members of the coastal Warli population who consume dairy approximately once per week had lower relative abundances of *Bifidobacterium* and *Ligilactobacillus* (mean 1.1% combined) than the Trans-Himalayan populations (mean 13.8% combined), but higher abundances than the Gond, Madia, and Kabui (mean 0.1% combined), who never consume dairy due to cultural taboo. Interestingly, the Trans-Himalayan populations also had lower relative abundance of *Segatella* than populations in all three other regions (Figure 4 Supplement 2), which had mostly plant-based diets. These patterns of *Bifidobacterium*, *Ligilactobacillus*, and *Segatella* relative abundance were also replicated in the metagenomic data (Figure 4 Supplement 2).

To explore the extent to which dairy consumption might drive these patterns, we searched the literature for evidence linking dairy farming, fermentation, or metabolism to the 18 bacterial taxa enriched in the Trans-Himalayan populations versus all three other regions. Remarkably, all 18 taxa have been previously associated with cattle or milk production in the scientific literature (Figure 4B; Table S3). For example, *Ligilactobacillus, Megasphaera*, *Collinsella*, and *Lactobacillus* have been detected directly in fermented milk products like yogurt and hard cheeses. Some *Bifidobacterium* have also been found in raw or fermented milk, but the species are distinct from those found in human guts, implying a distinct ecological niche. These genera as well as *Parolsenella, Oribacterium*, and *Faecalimonas* have also been experimentally shown to ferment lactose, a natural sugar found exclusively in mammalian milk. Additional taxa including Rikenellaceae_RC9_gut_group, NK4A214_group, *Monoglobus*, and Lachnospiraceae_NK3A20_group have also been detected in raw milk. Furthermore, 17 of the top 18 enriched taxa have been found in the cattle rumen microbiome, 16 in ruminant feces, and 13 in the cattle reproductive system (Figure 4B; Table S3). These findings suggest that the dairy-rich diet and lifestyle of the Trans-Himalayan populations could potentially be drivers of their distinct gut microbiome composition and elevated diversity.

### Abundant *Bifidobacterium* associated with functional shifts in gut sugar metabolism

The high abundance of *Bifidobacterium* in populations that regularly consume milk could impact the functional ecology of fermentation in the gut, such as the conversion of dietary carbohydrates into short-chain fatty acids (SCFAs). Mammalian milk is the only natural dietary source of lactose, a disaccharide composed of galactose and glucose.^68^ Gut bacteria in the order Bifidobacteriales encode a unique fermentation pathway known as the “bifid shunt” that produces the SCFAs acetate and lactate from hexoses like galactose and glucose.^69^ In the presence of lactose, *Bifidobacterium* species have been shown to upregulate lactate production and L-lactate dehydrogenase activity and preferentially deplete lactose from media that also contains glucose.^70^ ^,^ ^71^ Using our gut metagenomic data, we tested the hypothesis that dairy consumption and high abundance of *Bifidobacterium* was associated with lactose, glucose, and galactose fermentation capacity by quantifying functional pathways using the software HUMAnN 2.0.^72^

**Figure 5.**
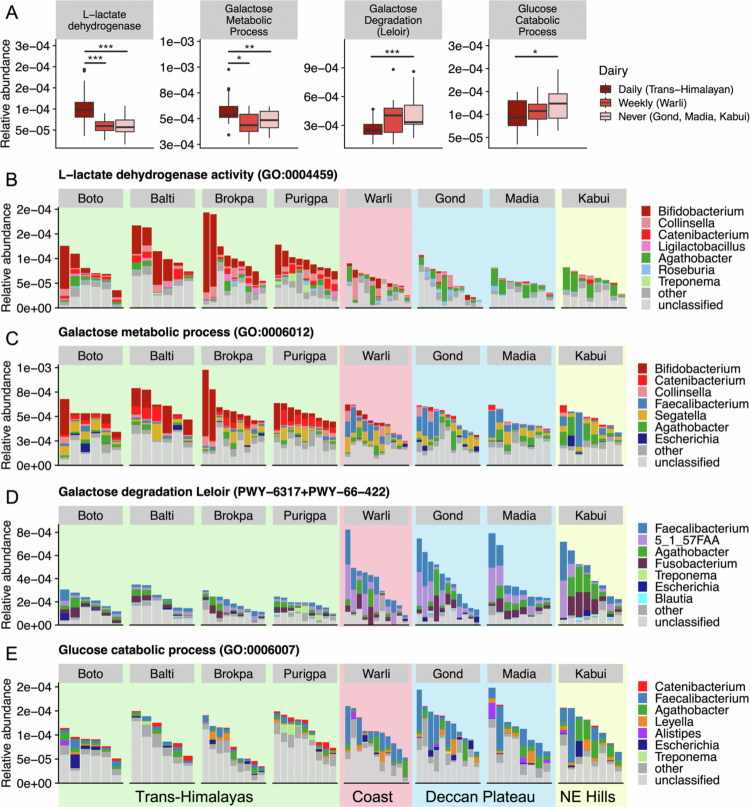
Dairy consumption is associated with functional shifts in sugar metabolism across populations, driven in part by abundant *Bifidobacterium*. **a,** Mean relative abundance of four GO and Metacyc functional pathways for lactose, galactose, and glucose catabolism that vary with dairy consumption. Three additional pathways are shown in Figure 5 Supplement 1. Asterisks represent results of Student’s t-tests. ***p < 0.001, **p < 0.01, *p < 0.05. **B–E**, Contribution of metagenomic genera to the pathways from **A**, quantified with HUMAnN 2.0. Lachnospiraceae_bacterium_5_1_57FAA is abbreviated 5_1_57FAA.

Metagenomic sequences with ontology to L-lactate dehydrogenase, a key enzyme in the Bifid shunt, were significantly more abundant in samples from populations that consumed dairy daily (Trans-Himalayan) versus weekly (Warli, *p* = 1.1 × 10^−5^, t-test) or never (Gond, Madia and Kabui, *p* = 5.9 × 10^−7^, t-test, Figure 5A). As expected, *Bifidobacterium* contributed a plurality of genes to this function in the Trans-Himalayan populations (mean 32%), while in all populations, diverse other taxa including *Agathobacter* and *Roseburia* encoded homologous genes (Figure 5B). A distinct “Lactose and Galactose Degradation” pathway (Metacyc) was also enriched in the Trans-Himalayan populations versus those who never consumed dairy (*p* = 0.006, t-test, Figure 5 Supplement 1A), driven by the presence of *Ligilactobacillus* rather than *Bifidobacterium* (Figure 5 Supplement 1B).

In contrast to lactose, galactose is a ubiquitous monomer of carbohydrates found in many organisms, including edible plants and human gut mucus. We identified two phylogenetically distinct pathways for galactose degradation that differed among gut microbiomes in relation to dairy consumption. “Galactose Metabolic Process” encoded by *Bifidobacterium* and *Catenibacterium* was more abundant in Trans-Himalayan guts (*p* ≤ 0.011, t-tests, Figure 5A,C), whereas the Leloir pathway encoded by *Faecalibacterium*, *Agathobacter*, and Lachnospiraceae_bacterium_5_1_57FAA was significantly more abundant in populations that never consumed dairy (*p* = 2.5 × 10^−4^, t-test, Figure 5A,D).

Finally, pathways for glucose catabolism and transport were significantly less abundant in samples from the Trans-Himalayas than samples from other regions (Figure 5A, Figure 5 Supplement 1 A, *p* ≤ 0.014, t-tests). *Faecalibacterium*, *Agathobacter*, and *Leyella* made substantially larger contributions to glucose metabolism in populations that consumed little or no dairy compared to those that consumed dairy daily (Figure 5E). Taken together, the data suggest that the high abundance of *Bifidobacterium* associated with daily dairy consumption may shift the functional capacity of the gut microbiome away from glucose metabolism and towards specific types of lactose and galactose metabolism. Since gut species of *Bifidobacterium* are not typically found in fermented milk products, their high abundance in Trans-Himalayan guts could potentially result from selective pressure exerted by dairy-associated dietary carbohydrates on resident strains within the gut microbiome.

### Endemic strains of *Bifidobacterium adolescentis* encode distinct CAZymes

Gut *Bifidobacterium* are highly responsive to human diet thanks to their large repertoire of carbohydrate-active enzymes (CAZymes), many of which act on plant fibers indigestible to the human host.^73^ In addition to the presence of milk products, a unique feature of the Trans-Himalayan diets compared to the other groups was regular consumption of wheat and barley alongside the widespread staple of rice (Figure 1C). We performed additional metagenomic analysis to further explore the genetic capacity for carbohydrate degradation present in *Bifidobacterium* MAGs.

Across all populations, *B. adolescentis* was the most prevalent and abundant of the nine *Bifidobacterium* species in our metagenomic dataset, followed by *B. longum* and *B. sp002742445* (Table S4). *B. adolescentis* is prevalent in adult gut microbiomes around the world and has been associated with many aspects of gut health, including maintenance of gut barrier integrity.^74^ While best known as a specialist on plant-derived glycans, many strains of *B. adolescentis* grow well on lactose and glycogen in addition to plant carbohydrates.^75^ We obtained 20 *B. adolescentis* MAGs from our samples with mean 90% completeness and 1.6% contamination and compared them to 112 publicly available *B. adolescentis* MAGs and isolate genomes from around the globe (Table S5), using the type strain ATCC 15703 as a reference.

**Figure 6.**
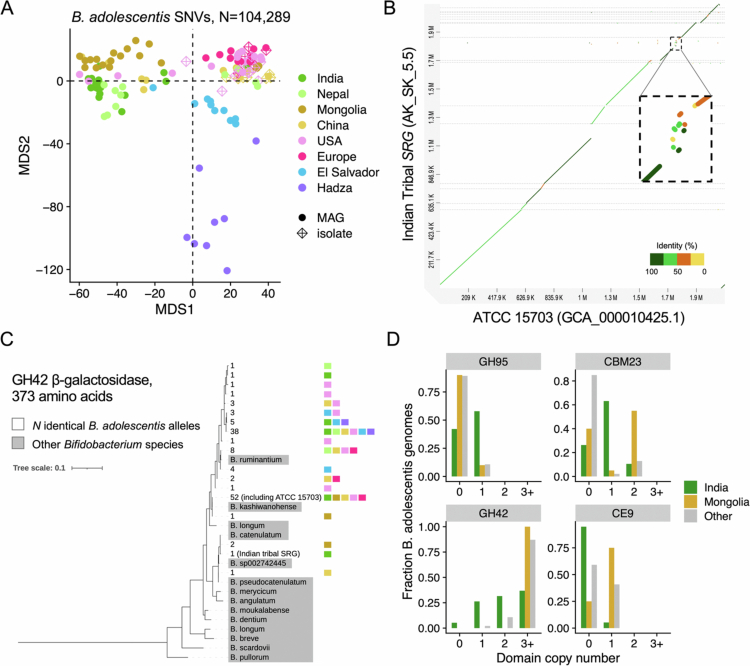
*Bifidobacterium adolescentis* strains endemic to Indian populations encode CAZyme variation under positive selection. **A**, Multidimensional scaling (MDS) plot of 104,289 biallelic SNVs from 133 *B. adolescentis* genomes sampled from human guts worldwide. **B**, Dot plot of an alignment between the *B. adolescentis* reference genome ATCC 15703 and the species-representative MAG assembled in this study. The inset highlights a region of low-quality alignment beginning in a gene containing a GH42 *β*-galactosidase domain. **C**, Phylogenetic tree of GH42 amino acid sequences from gut *Bifidobacterium.* Identical *B. adolescentis* gene sequences are collapsed for clarity, with the number on each tip representing the number of identical sequences. Colors are as in **A**, indicating the geographical location from which genomes containing each allele were sampled. **D**, Four CAZyme families occur at significantly higher copy number in *B. adolescentis* genomes from India (*N* = 20) and/or Mongolia (*N* = 20) than industrialized populations (*N* = 63). Benjamini-Hochberg-corrected *p*-values < 0.05 from one-sided t-tests are: GH95 *p* = 0.010 India; CBM23 *p* = 0.010 India, *p* = 0.010 Mongolia; GH42 *p* = 0.044 Mongolia; CE9 *p* = 0.044 Mongolia.

Multidimensional scaling (MDS) analysis of 104,289 biallelic SNVs across these 133 *B. adolescentis* genomes revealed strong genetic structuring by geography and host lifestyle (Figure 6A). In particular, Indian MAGs formed a cluster with MAGs from transitional populations in nearby Nepal, which was separated by MDS1 from most other global strains except those from Mongolia. Mongolian *B. adolescentis* MAGs were previously shown to be enriched for *β*-galactosidase (or lactase) genes, consistent with the importance of dairy in traditional Mongolian diets.^76^ Similarly, MDS2 separated all other global strains of *B. adolescentis* from MAGs from the Hadza hunter-gatherers of Tanzania, who consume high volumes of fiber but little to no dairy.^77^ A third major cluster consisted of *B. adolescentis* MAGs and isolates from China, Europe, and the USA. Consistent with these patterns, phylogenetic analysis of the 104,289 SNVs demonstrated more robust evidence for monophyly among Indian strains than among strains from China, Europe, and the USA (Figure 6 Supplement 1). These results suggest an evolutionary history of geographical constraint and potential host specialization for *B. adolescentis* that, in industrialized populations subject to globalization, may have begun to be overwritten. Nonetheless, the gut microbiomes of Indian populations still contain local strains of *B. adolescentis* that are genetically distinct from others around the globe.

To search for genomic regions unique to Indian *B. adolescentis*, we plotted the alignment between the SRG assembled in this study and the genome of type strain ATCC 15703 (Figure 6B). The two *B. adolescentis* genomes were 98.4% identical across 80% of their length, confirming that they belonged to the same species despite differences in gene content.

We investigated a poorly-aligned region of ~70 kb that was salient in the dot plot (Figure 6B) and found that it was immediately adjacent to four genes encoding CAZymes. Specifically, the alignment failed partway through a gene belonging to the GH42 family of *β*-galactosidases, which cleave *β*-linked galactose in carbohydrates such as lactose.

To understand the evolution of the Indian GH42 allele, we annotated CAZymes in the genomes of 26 gut *Bifidobacterium* species and 133 *B. adolescentis* strains (Table S5) and built a phylogenetic tree from homologous GH42 amino acid sequences (Figure 6C). This analysis revealed 18 variants of this GH42 domain among global *B. adolescentis* that differed by at least one amino acid. The two most abundant alleles were present in 52 (39%) and 38 (29%) of *B. adolescentis* genomes, respectively, and were detected in many regions of the world (Figure 6C), inconsistent with neutral evolution via geographical population structure (Figure 6A). Furthermore, *B. adolescentis* alleles in the gene tree were interspersed with alleles from other *Bifidobacterium* spp. including *B. ruminantium*, *B. kashiwanohense*, and *B. catenulatum* (Figure 6C). For example, the GH42 domain encoded by the Indian SRG differed by only 1/373 amino acids (0.3%) from the domain encoded by *B. catenulatum* (GenBank: AP012325.1), but by 29 amino acids (7.8%) from the *B. adolescentis* type strain ATCC 15703 and by an average of 28 amino acids (7.5%) from other Indian strains. This pattern is suggestive of a history of horizontal gene transfer between closely related *Bifidobacterium* species (Figure 6 Supplement 2).

The dN/dS ratio of the GH42 domain alignment was 0.091, indicating purifying selection overall. However, 23 of 373 codons had higher rates of non-synonymous than synonymous substitution (Table S6). Using a likelihood ratio model,^78^ we detected significant evidence of episodic diversifying selection on specific codons and branches of the GH42 tree (BUSTED *p* = 0.0016). At 10 codons, the major allele detected among Indian *B. adolescentis* was positively selected on branches sampled from the USA and/or China. Three codons were also positively selected on branches leading to Indian MAGs, including a valine at position 309 that was not observed in any other *B. adolescentis* genomes. Overall, substantial recombination of this GH42 *β*-galactosidase domain within and between *Bifidobacterium* species appears to have shuffled non-synonymous variation subject to diversifying selection in human guts across the world. This result highlights the relative mobility and rapid evolution of one CAZyme family, in contrast to the geographical population structure of genome-wide SNVs (Figure 6A).

We next used copy number analysis to identify additional CAZyme families potentially under positive selection in Indian *B. adolescentis*, using GH42 in Mongolian MAGs as a positive control. Two CAZyme families, GH95 and CBM23, were significantly enriched in Indian *B. adolescentis* compared to industrialized populations from around the world (Figure 6D). GH95 is a family of fucosidases and galactosidases that was detected in over half of Indian *B. adolescentis* MAGs, despite its absence from most other genomes of this species (Figure 6D; Drula et al.^79^ http://www.cazy.org/). While the function of this particular gene remains to be determined, GH95 is notably part of two PULs active on fucosyllactose and human milk oligosaccharides in *Bifidobacterium longum*, which is considered a breastmilk specialist.^80^ The majority of Indian *B. adolescentis* MAGs also carried one copy of CBM23, a mannan-binding domain that was absent from most industrialized *B. adolescentis* and also enriched in Mongolian MAGs. Mongolian MAGs were additionally enriched for GH42, as expected; as well as CE9, a family of *N*-acetylhexosamine phosphate deacetylases active in the metabolism of animal carbohydrates. Unlike Mongolian MAGs, Indian MAGs contained relatively few copies of GH42, despite evidence of diversifying selection on GH42 amino acid variation. These results demonstrate that the CAZyme content of human gut *B. adolescentis* varies with geography and host population, which could be consistent with variation in diet and lifestyle. In particular, the unique presence of milk-related domains in Indian MAGs could be consistent with adaptation to host diet via gain or loss of CAZymes.

## Gut microbiome CAZymes and substrates vary strongly with lifestyle and diet

Carbohydrate-active enzymes are essential to the fitness of bacteria competing for dietary carbohydrates in the host gut.^81-83^ As human diets change with industrialization, so too does the fermentation capacity of the gut microbiome, often embodied by shifts in *Bacteroides* and *Segatella* abundance. For example, the low-*Bacteroides* gut microbiomes of Hadza hunter-gatherers encode more diverse CAZymes with relatively more plant substrates and fewer mucin substrates than the high-*Bacteroides* microbiomes of U.S. residents.^84^ In light of the high abundance of *Bacteroides* observed in a small number of outlier samples (Figure 2), we were prompted to compare the CAZyme content of the Indian gut samples to a previously published sample of 37 healthy Californians, whose metagenomic data were assembled and quantified using the same protocol.^17^ ^,^ ^85^ For each sample from both studies, we constructed a CAZyme abundance profile using dbCAN2 to annotate CAZymes in each detected bacterial genome (see **Methods**).

**Figure 7.**
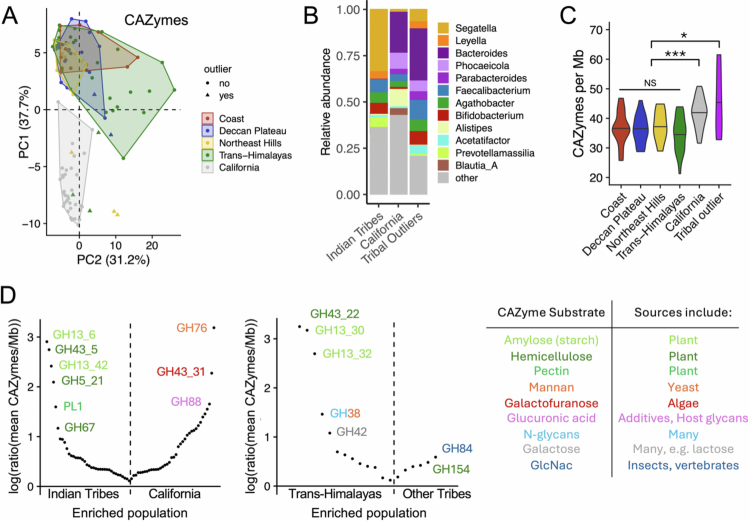
Gut microbiome CAZymes and carbohydrate substrates vary strongly by population. **A**, PCA of the density (genes/Mb) of 412 CAZyme families per gut microbiome. Indian outlier samples defined by high abundance of *Bacteroides* are excluded from shaded hulls. Californian samples are the average of two baseline samples of each subject from the FeFiFo study.^17^ ^,^ ^85^ **B**, Stacked bar plot of the average relative abundances of the top 12 genera detected by metagenomics. Rarer taxa are grouped as ‘other.’ **C**, Total density (genes/Mb) of 412 CAZyme families by region. Indian outliers with high *Bacteroides* are shown separately. ***p < 0.001, *p < 0.05, ^NS^ *p* > 0.05. **D**, Enrichment of CAZyme families by population (left) and summary of CAZyme dietary substrates (right). Points represent CAZyme families with gene density ≥0.1/Mb in at least one tested population and a Benjamini-Hochberg-adjusted Wilcoxon *p*-value ≤ 0.05 between populations. CAZyme families with the highest enrichment ratios are labeled with colors corresponding to substrates (right). GH38 targets include alpha-mannan, *N*-glycans, and high-mannose N-glycans. CAZyme substrates are derived from the Carbohydrate Active Enzymes database at http://www.cazy.org/ ^79^ and dbCAN-PUL^80^ and linked to dietary sources in Table S7.

PCA of the CAZyme abundance profiles revealed significant variation among Indian populations (R^2^ = 0.16, *p* = 0.003, PERMANOVA) and regions (R^2^ = 0.10, *p* = 0.001, PERMANOVA), with the Trans-Himalayan samples once again occupying a relatively distinct space (Figure 7A). The CAZyme profiles of the Californian samples clustered almost completely separately from the Indian samples (R^2^ = 0.19, *p* = 1 × 10^−4^, PERMANOVA), with the stark exception of previously mentioned outliers with high abundance of *Bacteroides* (Figure 7A,B). Unlike most Indian samples, the Californians and Indian outliers shared high relative abundances of *Bacteroides, Parabacteroides,* and *Phocaeicola* and low relative abundance of *Segatella* (Figure 7B). These similarities are unlikely to result from contamination of the Californian samples into the outlier subset of Indian samples, since *Alistipes* and *Blautia_A* occur at high relative abundance in the Californians and were not detected in the outliers. The average density of CAZymes encoded by the total gut microbiome did not vary among Indian populations (*p* = 0.76, ANOVA) or regions (*p* = 0.54, ANOVA) but was significantly higher in the Californians (*p* = 2.3 × 10^−7^, t-test) and Indian outliers (*p* = 0.04, t-test, Figure 7C). This result can be explained by the higher density of CAZymes in *Bacteroides* genomes than *Segatella* genomes in our dataset (mean 60.9 vs 45.1 genes per Mb, *p* = 3.6 × 10^−8^, KS test).

To link specific CAZyme families to dietary substrates, we first identified 76 CAZyme families that varied significantly in relative abundance between Indian (outliers excluded) and Californian microbiomes (Figure 7D). We then searched http://cazy.org/ and dbCAN-PUL for experimental evidence of carbohydrate substrates targeted by CAZyme families that were strongly enriched in one group versus the other.^79^ ^,^ ^80^ Many carbohydrates are synthesized by a subset of the domains of life, like plants or mammals, and are thereby linked to broad dietary categories (Table S7). In the Indian non-outlier samples compared to the Californians, we found six CAZyme families enriched by 10-1000X that have been shown to degrade plant polysaccharides like starch (GH13_6, GH13_42), hemicellulose (GH43), and pectin (PL1) (Figure 7D). In contrast, the top CAZyme families enriched in Californians degrade polysaccharides from yeast (GH76), seaweed (GH43_31), and animals (GH88). These functional differences in the gut microbiomes of globally distinct populations underscore the differences between the largely plant-based diets of the sampled Indian populations and more varied industrialized diets. However, identifying the drivers of microbiome shifts among the Indian outliers will require further in-depth investigation such as increasing metadata capture, larger sample sizes, and longitudinal sampling.

In an additional comparison among populations, we found that Trans-Himalayan gut microbiomes were ~1000X enriched for plant-targeting CAZymes in the GH13 and GH43 subfamilies compared to the four other Indian populations (Figure 7D). The Trans-Himalayan microbiomes were also ~10X enriched for GH42, a beta-galactosidase encoded by *B. adolescentis* and 618 other genomes in our dataset. These results are consistent with the regular presence of barley, wheat, and dairy in the diets of the Trans-Himalayan populations compared to the staple of rice for all populations. Overall, the large differences in CAZyme relative abundance between populations highlight taxonomic differences in the gut microbiome that may be shaped in part by differences in diet and lifestyle.

## Discussion

Since the launch of the Human Microbiome Project in 2007, a large body of research has demonstrated that microbial communities in the human gut play important roles in health^2^ ^,^ ^86^ and vary strongly with geography and lifestyle.^4^ ^,^ ^85^ ^,^ ^87^ ^,^ ^88^ In particular, diet is a modifiable component of lifestyle that interacts with the gut microbiome to shape health and disease.^7^ ^,^ ^11^ ^,^ ^13^ ^,^ ^14^ ^,^ ^17^ India has the largest indigenous population in the world outside of Africa, and tribal populations practice traditional diets shaped by culture and locally available foods.^89^ However, relatively few Indian tribal populations have participated in gut microbiome studies to date.^46^ In this study, we analyzed dietary habits and fecal samples from eight tribal populations in four biogeographic regions of India to explore the relationship between diet and non-industrialized gut microbiomes.

A number of dietary and microbiome features distinguished the Boto, Brokpa, Purigpa, and Balti populations located in the northwest Trans-Himalayas from the Gond, Madia, Kabui, and Warli populations located in other regions of India. Only members of the Trans-Himalayan populations reported daily consumption of wheat, barley, and (mostly non-fermented) dairy, in addition to never drinking alcohol. Gut microbiome diversity metrics were also elevated in the Trans-Himalayan populations, driven partly by conspicuous *Bifidobacterium* but also by low-abundance taxa shared with the rumen microbiome of cattle. This result suggests that bacterial transfer from cattle to humans, potentially via milk products or contact with dairy farmers, could help drive elevated diversity in Trans-Himalayan gut microbiomes. A similar association between gut microbiome diversity and livestock feeding chores was previously identified in children from Kenyan villages,^90^ while multiple studies have reported transfer of antibiotic resistance genes and/or bacteria between farm animals and humans.^91-93^ Although our study lacks direct sequencing of Trans-Himalayan livestock or milk products, environmental microbes including the well-documented microbiota of cattle could contribute to the distinct composition of the Trans-Himalayan gut microbiomes.

The prevalence and abundance of *Bifidobacterium* observed in Trans-Himalayan guts exceeds that of adults in most industrialized populations, but is similar to Ethiopian children in pastoral communities whose diets consist mainly of milk.^19^ ^,^ ^94^ This result raises the question of whether the *Bifidobacterium* in these populations are long-term gut residents and not transients frequently replenished by dairy consumption. *Bifidobacterium* are widely considered to be commensal gut taxa, and recent genetic evidence has demonstrated their co-diversification with humans.^95^ *Bifidobacterium* species are nearly ubiquitous in human guts of all ages and are associated with numerous positive health outcomes in both correlation and intervention studies.^19^ ^,^ ^96^ Bifidobacteria also devote 30% more genes to carbohydrate metabolism than other gut bacteria, enabling them to grow on a wide variety of dietary carbohydrates and quickly respond to host dietary changes.^19^ ^,^ ^73^ Unlike *Lactobacillus* and *Streptococcus*, *Bifidobacterium* are also not found at high abundance in naturally fermented milk products.^97^ ^,^ ^98^ In contrast, *Bifidobacterium* strains originally isolated from human infant feces are commonly added to industrially-fermented dairy to serve as probiotics.^99^ ^,^ ^100^ Given this context, it is more likely that highly abundant *Bifidobacterium* in Trans-Himalayan guts are long-term residents ecologically selected by diet, rather than poorly-adapted transients repeatedly replenished by fermented dairy. The functional shifts that we observed in galactose and glucose metabolism further support the notion that dietary lactose may select for *Bifidobacterium* in the gut.

In mammals and all prehistoric humans, lactase is produced in the small intestine during infancy and is downregulated after weaning.^101^ ^,^ ^102^ Although lactase persistence into adulthood has been positively selected in some human populations,^103^ nearly two-thirds of modern humans are lactase non-persistent, including 70-100% of people sampled across India.^21^ ^,^ ^104-106^ The absence of host lactase in the small intestine allows dietary lactose to reach the colon, where it is fermented by gut bacteria.^102^ As a consequence, one of the few replicated associations between the gut microbiome and human genotype is the association between *Bifidobacterium* and lactase non-persistence.^22^ ^,^ ^107^ In non-persistent adults, greater dairy consumption is associated with higher abundance of *Bifidobacterium,* ^108^ which has itself been associated with experimental acquisition of lactose tolerance.^109^ Such findings suggest a mutually beneficial relationship between *Bifidobacterium* and lactose non-persisters who consume dairy, which may also apply to the Trans-Himalayan populations (though their lactase persistence status is unknown). Intriguingly, recent studies have found that lactase persistence evolved independently of the spread of dairy farming and is poorly associated with milk consumption in India as well as the United Kingdom.^21^ ^,^ ^101^ The potential selection by dairy products of commensal *Bifidobacterium* in lactase non-persisters could even have helped establish dairy as an important component of nutrition and culture around the world.

An important limitation of this study is the near-perfect correlation among populations between geography and diet, which partially confounds associations between diet and the microbiome. Specifically, all four Trans-Himalayan populations are located fairly close together and consume fairly similar diets, but are located at a great distance from the other four populations who share a qualitatively different diet. This pattern occurred because our study was designed to sample four rural locations without prior knowledge of community diets, which were unexpectedly similar among Boto, Brokpa, Balti, and Purigpa despite their distinct lineage and cultures. Additional limitations include the modest sample size from each population and the restricted availability of participant metadata (Table S1), which limit statistical power and may obscure the effects of known confounders like age and sex on the gut microbiome. For example, the higher BMI observed from the limited data available in the Trans-Himalayan and Kabui populations could be related to microbiome composition in a number of ways. Nonetheless, we present functional metagenomic analyzes suggesting that both dairy and plants (such as cereals) may contribute to the unique composition of Trans-Himalayan gut microbiomes. While a cross-sectional population study like this one cannot prove causal links between diet and the gut microbiome, it can identify intriguing associations to be explored with future experiments or replicated in other populations.

Tribal populations in India, like many other non-industrialized populations, are a unique and under-sampled resource for understanding the human gut microbiome.^85^ ^,^ ^110^ ^,^ ^111^ Even the modest depth of metagenomic sequencing performed in this study uncovered fourteen novel bacterial species and 200 local strains, suggesting that much more microbial diversity remains to be discovered in the guts of undersampled populations. We nonetheless observed an unusually high abundance of *Bacteroides* in several outlier subjects, replicated across DNA extractions and sequencing methods, which suggests that a path to industrialization for these populations may have already begun. Previous studies have found that local gut microbiome signatures can be interrupted almost immediately, as in the case of Hmong and Karen individuals immigrating from Thailand to the US^44^; or over decades of lifestyle change, as in the case of Nepalese populations on a lifestyle gradient.^41^ In the future, metagenomic sequencing from many more Indian populations paired with standardized data on diet and lifestyle will be key to deepening our understanding of microbiome evolution in India. This study of eight traditional populations with diverse diets provides new insight into human-microbiome relationships that appear, as of yet, relatively undisturbed by industrialization.

## Materials and methods

### Ethical approval and sample collection

Ethical approval for the collection of human stool samples was obtained from the Institute Ethics Committee of the BRIC-National Center for Cell Science, India (approval number NCCS/IEC/2017-II/5). Verbal and written consent was obtained from all participants. The scientific aims of the study were verbally explained to participants, with the help of a translator when required.

Over a single summer season (June to September 2019), approximately 10 healthy adults (5 men and 5 women) were sampled from each of the eight populations. Height and weight measurements were taken from each individual to calculate BMI. Healthy adults were defined as persons between 18 and 55 y old without known infectious or chronic ailments, including hypertension, cardiovascular disease, and diabetes. Additional exclusion criteria included antibiotic treatment within 30 d prior to sampling; gastrological ailments including diarrhea, constipation, or ulcers within 30 d; surgery within 90 d; current pregnancy or breastfeeding; and non-endogamy within the last three generations (Supplementary Files 3 and 4).

Each participant collected at least 10 g of their own stool sample using kits and instructions provided by the study team, including Sterile Clinicol (PW015, HiMedia Laboratories), sterile collection paper, sterile gloves, and a set of visual instructions. Sampling containers were collected from participating individuals immediately and processed on-site by aliquoting 10 g of feces and adding 15 ml of DNA stabilization buffer (RNA Liv^TM^ ML161, HiMedia Laboratories). Field negative controls were also collected, defined as exposing a sterile collection container to field site air for 60 seconds (roughly the same time taken to transfer fecal samples) before adding 15 ml of DNA stabilization buffer. Processed samples were stored in refrigerators at 4 °C or coolers with ice packs for 1–2 hours on the day of sampling, which was different for each population over the course of the summer season, before being transported back to the laboratory and preserved at –20 °C initially and –80 °C for long-term. Samples were stored for varying periods, as DNA extraction was performed after all sampling was complete. Warli and Kabui were sampled in June 2019; Gond and Madia in October 2019; and the Trans-Himalayan populations in August 2019. DNA extraction for all was done in December 2019.

#### Dietary analysis

Qualitative dietary data was collected from a 24-hour dietary recall (Supplementary File 1) and a seasonal Food Frequency Questionnaire (Supplementary File 2) completed by each participant. Foods reported on the seasonal food frequency questionnaires were used to define dietary features unique to particular populations. Based on the responses collected, individuals not consuming traditional diets (e.g. traveling to markets and consuming fried foods) were excluded from the study. Heatmaps of dietary data were plotted in R version 4.2.0 “Vigorous Calisthenics”^112^ using the base function heatmap and package Cairo.^113^

## 16S rRNA gene amplicon sequencing and analysis

DNA was extracted from fecal samples suspended in DNA stabilization buffer (RNA Liv^TM^ ML161, HiMedia Laboratories) using an initial step of bead-beating followed by the QIAamp DNA Mini Kit (Qiagen). All fecal samples were processed for DNA extraction in a single batch, with field and reagent negative controls also processed and sequenced with the same protocol. Extracted DNA concentrations were estimated using a Nanodrop Spectrophotometer (ThermoScientific) and normalized among samples. The V4 region of the 16S rRNA gene was amplified by PCR using published primers.^114^ Amplified PCR products were purified using AMPure XP beads (Beckman Colter Life Sciences). Sequencing adapters were added to the purified PCR products using the Nextera XT v2 Dual Index Kit with 96 indices (Illumina). The final product was quantified using a Qubit Fluorometer (ThermoFisher Scientific). For sequencing, 5 uL of a 4 nM library (size 420 bp) was loaded onto the cartridge of the Miseq Reagent Kit V2 (Illumina), with 2 µL of 10 nM PhiX Control v3 (Illumina) added as a positive control. 2 × 250 bp paired-end sequencing was performed on the MiSeq platform using the MiSeq Reagent Kit V2, 500 cycles (Illumina). To minimize batch variation, all 16S samples were run on the same MiSeq cartridge.

Raw amplicon reads were demultiplexed and processed to ribosomal sequence variants (RSVs) using the DADA2 analysis pipeline.^47^ Taxonomy was assigned using SILVA 138.2,^115^ and plots were created using custom scripts in R v4.2.0.^112^

### Metagenomic library preparation and sequencing

DNA extracted from fecal samples using the QIAamp DNA Mini Kit (Qiagen) was purified using PEG (polyethylene glycol) NaCl and dried before shipping from Pune to Stanford. Metagenomic libraries were prepared from 0.072 ng of rehydrated input DNA on randomized plate positions using the Nextera XT v2 Dual Index Kit with 12-bp barcodes (Illumina). Libraries were quantified using a Fragment Analyzer (Agilent) and size-selected using AMPure XP beads (Beckman Colter), targeting a fragment length of 450bp. Paired-end sequencing (2x140bp) was performed on an Illumina NovaSeq 6000 using S4 flow cells at the Chan Zuckerberg Biohub (San Francisco, CA, USA).

Raw metagenomic reads were processed using software in the BBtools suite.^116^ Specifically, exact duplicate reads (subs = 0) potentially representing PCR duplicates were marked using clumpify. Adapters and low-quality bases were trimmed using bbduk (trimq = 16 minlen = 55). To exclude host sequences, trimmed reads were mapped using BBmap against the human genome (hg19) with masks over regions conserved broadly in eukaryotes. FastQC (https://www.bioinformatics.babraham.ac.uk/projects/fastqc/) was used as a final check for sequence quality, with no modules reporting errors.

### Metagenomic assembly and genome quantification

Individual metagenomes were assembled for each sample using metaSPAdes v3.13^117^ with default error correction enabled and the following k-mer sizes: 21, 33, 55, and 77. QUAST v5.0^118^ was used to evaluate assembly size and contig metrics, and metagenome assemblies were filtered to only include contigs ≥ 1500 base pairs. Reads from each sample were mapped together with other samples from the same population using Bowtie 2 (--very-sensitive -X 1000)^119^ to generate coverage profiles for binning, which benefits from using related samples. Binning is the process of grouping assembled contigs into draft genomes (MAGs) by clustering sequences that show similar nucleotide composition and abundance patterns across samples. Because contigs originating from the same organism are expected co-vary in abundance across related samples, multi-sample coverage profiles substantially improve the accuracy and completeness of genome reconstruction compared with single-sample coverage alone. Genome binning was performed with MetaBAT2 v2.13^120^ using default settings and contig depth information from all samples. Genome bin quality was assessed using CheckM v1.1.2,^121^ and genome taxonomy was assigned using GTDB-Tk v1.7 with database release R205.^122^

Medium-quality genomes with ≥50% completeness and <15% contamination, per MIMAG standards,^123^ were next combined with genomes from the Unified Human Gastrointestinal Genome Collection (UHGG v1)^59^ to create a non-redundant species-level genome database for read mapping. These genomes were de-replicated into species-level groups using dRep,^124^ which assigned genomes that share ≥95% average nucleotide identity (ANI) over 30% of their length to the same species.

The genus-level taxonomy of genomes classified as *Prevotella* by GTDB-tk was manually updated to current genera (e.g., *Segatella, Leyella, Xylanibacter*, *Hallella* ^32^) based on a phylogenetic tree computed from NCBI reference genomes using GToTreev1.6.3^125^ (Figure 2 Supplement 6). The taxonomy of *Prevotella* genomes with unclear placement in the tree was not specified below family level. Placeholder species names from GTDB-tk were retained (e.g. *Hallella sp002299635* in place of *Prevotella sp002299635*). Newly-defined species within the *Segatella copri* complex^31^ were not considered for the purposes of this work because their genome assemblies were not publicly available.

A single .fasta file was created by concatenating all species representative genomes together. Reads from each sample were mapped to the concatenated file using Bowtie 2 with default settings. “inStrain profile” and “inStrain quick_profile” with coverM were run on all resulting mapping files with inStrain v1.5.2 on default settings.^48^ ^,^ ^126^ Detection of a species in a sample was defined as that species representative genome being present with ≥0.5 breadth, meaning that at least half of the bases in the genome were covered by at least a read.

### Metagenomic enrichment samples

One gram of fecal sample from each of five Warli participants, who were most closely located to the laboratory, were aliquoted in the field directly into 10 mL of sterile, pre-reduced phosphate-buffered saline (PBS) in Falcon tubes to minimize exposure of viable gut microorganisms to environmental oxygen. The samples were homogenized in the Falcon tubes, and 1 ml of the homogenate was transferred using a sterile syringe into sterile, pre-reduced PBS in stoppered glass culture bottles. The vials were transported back to the laboratory under refrigerated conditions using gel packs to preserve microbial viability.

In the laboratory, 1 ml of each sample was inoculated in duplicate into two culture media—Robertson’s Cooked Meat Medium (HiMedia M149S) and Gifu Anaerobic Broth (HiMedia M1801)—under anaerobic conditions and incubated at 37 °C for 15 d to support the growth of both fastidious and non-fastidious gut microorganisms. Following incubation, 1 ml from each culture was aseptically transferred into microcentrifuge tubes using a sterile syringe. Samples were centrifuged at 14,000 rpm for 10 minutes to separate suspended cells from the culture media. The resulting pellets were then subjected to the same metagenomic sequencing and assembly protocol as the original fecal samples. While further characterization of the enrichment samples is beyond the scope of this study, 22 bacterial genomes assembled from metagenomic sequencing of these community samples were retained for mapping and quantification in the fecal samples (see Table S2).

### Gut microbiome data from other studies

Relative abundance data analyzed in this work that originated from other studies are available at https://github.com/emily-ebel/indian-gut-microbiome.

To perform comparisons with urban Indians, we located publicly-available 16S relative abundance data from a study^30^ that sampled feces from ~80 healthy adults in the large city of Ahmedabad, Gujarat. Available metadata from that study included individual health metrics, age, and some dietary data. We were unable to locate comparable relative abundance data with associated MAGs from a metagenomic study of urban Indian gut microbiomes.

To perform comparisons with urban Californians, we located publicly-available 16S relative abundance data from the FeFiFo study^17^, which sampled feces from ~40 healthy adults in the San Francisco Bay Area. These subjects provided detailed demographic, physiologic, and dietary data including food logs. We used the average genus-level relative abundance per subject over two baseline timepoints sampled three weeks apart, prior to the study’s dietary intervention. For metagenomic analysis of CAZymes from these samples, we utilized inStrain relative abundance data and MAGs assembled from ultra-deep resequencing, as described in Carter et al.^85^

### Statistical analysis

All statistical analyzes were performed in R (version 4.2.0). Relative abundances were calculated using depth of reads with taxonomic assignments. Mean values among groups were compared using ANOVA followed by pairwise comparisons using Tukey’s Honest Significant Differences test. Mean values were compared between pairs of groups using Student’s t-test (parametric) or Wilcoxon’s rank-sum test (non-parametric), followed by Benjamini-Hochberg correction for multiple testing. Beta diversity was assessed using Bray-Curtis dissimilarities and visualized by principal component analysis (see below). Group differences in community composition were tested by PERMANOVA (adonis function in *vegan*, 100001 permutations). Relationships between pairs of continuous variables were evaluated with linear models. Unless otherwise noted, *p* < 0.05 was considered statistically significant.

Differentially abundant taxa were identified using Maaslin2 on 16S rRNA gene’s relative abundance data without normalization or prevalence filtering, using Region as a fixed effect with Trans-Himalayas as the reference. Age, sex, and BMI were omitted as fixed effects because 10-25% of subjects declined consent for the use of these categories of metadata (Table S1).

### Principal component analysis

Relative abundances for each taxon and sample were arcsin square root transformed and used to calculate Bray-Curtis distances between samples with the vegan package in R.^112^ ^,^ ^127^ The resulting distance matrix was scaled and centered. Principal component analysis (PCA) was performed using the functions “dudi.pca” and “get_eigenvalue” from the packages ade4 and factoextra.^128^ ^,^ ^129^ PCA plots were visualized using ggplot2 and “chull” function from grDevices.^130^

### Definition and exclusion of outlier samples

Outliers were initially noted in PCA plots of taxonomic beta diversity using both 16S (*N* = 6) and metagenomic (*N* = 8) data. To understand why these samples did not cluster with their populations, we examined stacked bar plots for each subject and data type and noted that all but one PCA outlier sample had atypically high relative abundance of *Bacteroides*. The single exception was AK_SG_17 (Madia) in the metagenomics data, which instead had atypically high relative abundance of *Phocaeicola.* We noted that six of the seven high-*Bacteroides* metagenomics outliers also contained appreciable relative abundances of *Phocaeicola* and *Parabacteroides*, which were previously classified as *Bacteroides.* ^49^ ^,^ ^50^ Because these taxa are usually rare in the gut microbiomes of non-industrialized populations, we chose to define outlier samples as those with a combined relative abundance of *Bacteroides*, *Phocaeicola*, and *Parabacteroides* greater than 7% in the metagenomics data and 13% in the 16S data.

Six outlier samples met this “high *Bacteroides*” criterion in both the 16S and metagenomics data. Of the remaining two PCA outliers, AK_SR_2 (Kabui) had high *Bacteroides* in the metagenomics data but failed 16S sequencing, while AK_SR_1 (Kabui) had high *Bacteroides* in the metagenomics data but typical composition and PCA placement in the 16S data. We chose to classify AK_SR_1 as a high-*Bacteroides* outlier along with the seven other high-*Bacteroides* samples apparent in the metagenomic species PCA. Consequently, eight total high-*Bacteroides* outliers were excluded from the following analyzes with either data type: core microbiome (Figure 3), relative abundance (Figure 4), functional metagenomics (Figure 5), and population CAZyme enrichment (Figure 7D).

Finally, we noted that sample AK_SR_4 (Kabui) had high relative abundance of *Streptococcus* in the 16S data (66%) as well as the metagenomics data (17%). Although this sample was not considered a high-*Bacteroides* outlier, it was also excluded from all subsequent analyzes.

### Functional metagenomic pathways and CAZyme analysis

HUMAnN 2.0^72^ was used to profile metagenomic samples for functional microbial pathways in the MetaCyc and Gene Ontology databases. We manually identified six pathways related to lactose, glucose, and galactose fermentation and compared their relative abundances among dietary groups using Student’s t-test.

Genes encoding carbohydrate-active enzymes (CAZymes) were identified in bacterial genomes using an HMMER search against the dbCAN HMM database implemented in dbCAN2.^131^ Custom R scripts were used to count CAZyme family members in each bacterial genome, normalize counts by genome length, and create a CAZyme profile for each metagenomic sample weighted by the relative abundance of each genome. PCA of CAZyme profiles was performed as described above, except without the arcsin square root transformation. To calculate enrichment of CAZyme families in one population versus another (i.e., California vs. all Indian populations or Trans-Himalayan vs. other populations), the number of genes per family per Mb per sample were compared across populations using the Wilcoxon Rank Sum Test in R. The Benjamini-Hochberg method was used to adjust *p*-values for multiple testing. To further reduce potential false positives for rare CAZyme families, we also excluded families with mean relative abundance <0.1 gene/Mb in both populations.

Most CAZyme families contain multiple genes that have been experimentally proven to degrade certain carbohydrates. To better understand how different foods supply various carbohydrates to the gut microbiome, we manually annotated the dietary sources of substrates included in dbCAN-PUL (Table S7).^80^ To better understand the function of CAZyme families of interest, we manually combined this dietary information with enzyme functions annotated in http://cazy.org/. ^79^

### *Bifidobacterium adolescentis* genetic variation

To compare Indian *B. adolescentis* MAGs to strains from human guts around the globe, we downloaded an ad hoc set of 112 geographically diverse *B. adolescentis* isolate genomes and MAGs from NCBI and UHGG/MGnify (Table S5). We also gathered 11 *B. adolescentis* MAGs from Nepalese populations on a lifestyle gradient,^41^ 8 from Hadza hunter-gatherers in Tanzania,^84^ and 3 from healthy adults in California^17^ that were recently generated with ultra-deep sequencing.^85^

Homologous sites across *B. adolescentis* genomes were identified via alignment to the ATCC 15703 reference genome using ‘nucmer’ from MUMmer4 with default settings.^132^ Single-nucleotide variants (SNVs) were called using ‘show-snps’ and the following variants were removed: indels and SNVs within 1 kb of the end of an alignment; multiallelic SNVs; SNVs private to a single genome; and SNVs missing in over half of genomes. A final matrix of 104,289 SNVs was coded for multidimensional scaling analysis (MDS) using 0 and 1 for minor and major alleles, respectively. Euclidean distances between genomes were calculated from this matrix with the vegan package and MDS was performed using ‘cmdscale’ in R.^112^ ^,^ ^127^

A dot plot comparing the *B. adolescentis* SRG MAG from this study to the reference strain ATCC 15703 was created by uploading the .fasta files to the D-GENIES web interface (https://dgenies.toulouse.inra.fr/run) and choosing minimap2 v2.28 for alignment.^133^ ^,^ ^134^

### Phylogenetics and natural selection

A phylogeny for 133 *B. adolescentis* strains was constructed from the 104,289 SNVs described above using RAxML v8.0.0^135^ with the parameters ‘-m GTRCAT -p 199’. Reference genomes for 26 other *Bifidobacterium* species previously found in humans^136^ were downloaded from NCBI and used to construct a phylogeny with GToTree v1.6.3.^125^ Briefly, prodigal v2.6.3 and HMMER3 v3.2.2 were used to identify 74 single-copy target genes, which were individually aligned with muscle v5.1, trimmed with trimal v1.4.rev15, and concatenated prior to phylogenetic estimation with FastTree2 v2.1.11.^137-141^

To build a phylogeny of GH42 genes in *Bifidobacterium*, dbCAN2 was first used to predict CAZymes from the genomes of global *B. adolescentis* strains and other *Bifidobacterium* species. GH42 gene sequences were extracted from each genome and translated using a custom Python 3 script. Protein sequences were aligned using Clustal Omega v1.2.4,^142^ and a phylogeny was constructed using RAxML v8.0.0 with the parameters ‘-m PROTGAMMALGF -*p* 199’. This phylogeny revealed four distinct clades of GH42 in the sampled genomes. Only the clade containing the *B. adolescentis* allele from the poorly-aligned region shown in Figure 6B is shown in Figure 6C.

Tests of natural selection on GH42 in *B. adolescentis* were implemented on the Datamonkey 2.0 web server^143^ using the nucleotide version of the alignment shown in Figure 6C. Single Likelihood Ancestor Counting was used to calculate dN/dS overall,^144^ while BUSTED was used to identify codons subject to episodic positive selection.^78^

### Terminology

We acknowledge that the term “tribe” may be considered inaccurate and pejorative, given its historical connotations and roots in the colonial administration of India. The term “indigenous” is also contested in India and does not map cleanly onto constitutional categories.^145^ To avoid inaccuracy and ethical issues, we have replaced over 150 usages of “tribe” throughout the manuscript with the more neutral term “tribal populations/communities.” At the same time, the term “tribe” is actively used by Indian government administrators in the form of a Scheduled Tribe list for affirmative action schemes and benefits.^146-148^ The concept of ‘tribe’ thus survives in Indian anthropology as a ‘social and cultural entity’^148^ ^,^ ^149^ used by and for communities that not only see themselves as different from others, but are also seen as different by other communities, retaining a distinct cultural identity, having their own ethnonyms, cultural practices, diets, and in general a shared sense of belonging to that community. Anthropologists recommend studying the tribes in India in reference to this cultural identity.^145^ This cultural identity is also accentuated by the Indian practice of endogamy.^150-152^ In the context of biological studies, such endogamous groups with distinct cultural identity are units of evolutionary and molecular anthropological studies.^151^ ^,^ ^153^ ^,^ ^154^ More importantly, in the context of the present manuscript, are the dietary and culinary practices, which are cultural traits closely linked to the community's identity. Tribal communities in India are now being studied for their microbiome composition, given their unique and traditional dietary practices, which have stood the test of rapid food and lifestyle changes due to westernization.^45^

In this context, we use the concept of ‘Tribe/Tribal population’ as “a type of social formation without a centralized authority, an absence of specialized occupational groups, a society that is lineage-based, and having a relatively distinct culture” (Chophy^146^ p. 5; Srivastava^155^) and identify the communities under study collectively as Tribe/tribal population/community, but also identify individual populations by their own ethnonyms; namely, Warli, Gond, Madia, Kabui, Purigpa, Boto, Brokpa, and Boto. Our consistent use of “population” or “community” and the removal of the standalone word “tribe” follow established best practices in interdisciplinary and biomedical research to prioritize precision, respect, and the self-identification of the participating groups.^150^ ^,^ ^156^

## Supplementary Material

REVISED Supplemental Figures.pdf REVISED Supplemental Figures.pdf

Supplementary Materials excluding Figures.zip Supplementary Materials excluding Figures.zip

Supplementary Captions Supplementary Captions

Supp Files.zip Supp Files.zip

## Data Availability

Data analysis scripts and tables used in this study are available at https://github.com/emily-ebel/indian-gut-microbiome. Read data and genome assemblies generated by this study are publicly accessible on the short read archive (SRA) and GenBank under NCBI BioProject PRJNA1266865.

## References

[1] Bäckhed F , Ley RE , Sonnenburg JL , Peterson DA , Gordon JI . Host-bacterial mutualism in the human intestine. Science. 2005;307(5717):1915–1920. doi: 10.1126/science.1104816. 15790844

[2] Lynch SV , Pedersen O . The human intestinal microbiome in health and disease. N Engl J Med. 2016;375(24):2369–2379. doi: 10.1056/NEJMra1600266. 27974040

[3] Bokulich NA , Chung J , Battaglia T , Henderson N , Jay M , Li H , Lieber A , Wu F , Perez-Perez GI , Chen Y , et al. Antibiotics, birth mode, and diet shape microbiome maturation during early life. Sci Transl Med. 2016;8(343):343–382. doi: 10.1126/scitranslmed.aad7121. PMC5308924 27306664

[4] Clemente JC , Pehrsson EC , Blaser MJ , Sandhu K , Gao Z , Wang B , Magris M , Hidalgo G , Contreras M , Noya-Alarcón Ó , et al. The microbiome of uncontacted amerindians. Sci Adv. 2015;1(3):e1500183. doi: 10.1126/sciadv.1500183. 26229982 PMC4517851

[5] Dethlefsen L , Relman DA . Incomplete recovery and individualized responses of the human distal gut microbiota to repeated antibiotic perturbation. Proc Natl Acad Sci. 2011;108(supplement_1):4554–4561. doi: 10.1073/pnas.1000087107. 20847294 PMC3063582

[6] Rothschild D , Weissbrod O , Barkan E , Kurilshikov A , Korem T , Zeevi D , Costea PI , Godneva A , Kalka IN , Bar N , et al. Environment dominates over host genetics in shaping human gut microbiota. Nature. 2018;555(7695):210–215. doi: 10.1038/nature25973. 29489753

[7] Wu GD , Chen J , Hoffmann C , Bittinger K , Chen Y-Y , Keilbaugh SA , Bewtra M , Knights D , Walters WA , Knight R , et al. Linking long-term dietary patterns with gut microbial enterotypes. Science. 2011;334(6052):105–108. doi: 10.1126/science.1208344. 21885731 PMC3368382

[8] Blaser MJ . The past and future biology of the human microbiome in an age of extinctions. Cell. 2018;172(6):1173–1177. doi: 10.1016/j.cell.2018.02.040. 29522739

[9] Deehan EC , Walter J . The fiber gap and the disappearing gut microbiome: implications for human nutrition. Trends Endocrinol Metab. 2016;27(5):239–242. doi: 10.1016/j.tem.2016.03.001. 27079516

[10] Sonnenburg ED , Sonnenburg JL . The ancestral and industrialized gut microbiota and implications for human health. Nat Rev Microbiol. 2019;17(6):383–390. doi: 10.1038/s41579-019-0191-8. 31089293

[11] David LA , Maurice CF , Carmody RN , Gootenberg DB , Button JE , Wolfe BE , Ling AV , Devlin AS , Varma Y , Fischbach MA , et al. Diet rapidly and reproducibly alters the human gut microbiome. Nature. 2014;505(7484):559–563. doi: 10.1038/nature12820. 24336217 PMC3957428

[12] Flint HJ , Duncan SH , Louis P . The impact of nutrition on intestinal bacterial communities. Curr Opin Microbiol. 2017;38:59–65. doi: 10.1016/j.mib.2017.04.005. 28486162

[13] Ghosh TS , Rampelli S , Jeffery IB , Santoro A , Neto M , Capri M , Giampieri E , Jennings A , Candela M , Turroni S , et al. Mediterranean diet intervention alters the gut microbiome in older people reducing frailty and improving health status: the NU-AGE 1-year dietary intervention across five european countries. Gut. 2020;69(7):1218–1228. doi: 10.1136/gutjnl-2019-319654. 32066625 PMC7306987

[14] Lim J-M , Letchumanan V , Tan LT-H , Hong K-W , Wong S-H , Ab Mutalib N-S , Lee L-H , Law JW-F . Ketogenic diet: a dietary intervention via gut microbiome modulation for the treatment of neurological and nutritional disorders (a Narrative Review). Nutrients. 2022;14(17):3566. doi: 10.3390/nu14173566. 36079829 PMC9460077

[15] Lim RRX , Park MA , Wong LH , Haldar S , Lim KJ , Nagarajan N , Henry CJ , Jiang YR , Moskvin OV . Gut microbiome responses to dietary intervention with hypocholesterolemic vegetable oils. NPJ Biofilms Microbiomes. 2022;8(1):1–12. doi: 10.1038/s41522-022-00287-y. 35411007 PMC9001705

[16] Vijay A , Astbury S , Panayiotis L , Marques FZ , Spector TD , Menni C , Valdes AM . Dietary interventions reduce traditional and novel cardiovascular risk markers by altering the gut microbiome and their metabolites. Front Cardiovasc Med. 2021;8:691564. doi: 10.3389/fcvm.2021.691564. 34336953 PMC8319029

[17] Wastyk HC , Fragiadakis GK , Perelman D , Dahan D , Merrill BD , Yu FB , Topf M , Gonzalez CG , Van Treuren W , Han S , et al. Gut-microbiota-targeted diets modulate human immune status. Cell. 2021;184(16):4137–4153.e14. doi: 10.1016/j.cell.2021.06.019. 34256014 PMC9020749

[18] Aslam H , Marx W , Rocks T , Loughman A , Chandrasekaran V , Ruusunen A , Dawson SL , West M , Mullarkey E , Pasco JA , et al. The effects of dairy and dairy derivatives on the gut microbiota: a systematic literature review. Gut Microbes. 2020;12(1):1799533. doi: 10.1080/19490976.2020.1799533. 32835617 PMC7524346

[19] Derrien M , Turroni F , Ventura M , van Sinderen D . Insights into endogenous bifidobacterium species in the human gut microbiota during adulthood. TIM. 2022;30:940–947. doi: 10.1016/j.tim.2022.04.004. 35577716

[20] Rastogi S , Singh A . Gut microbiome and human health: exploring how the probiotic genus lactobacillus modulate immune responses. Front Pharmacol. 2022;13. doi: 10.3389/fphar.2022.1042189. PMC9638459 36353491

[21] Baadkar SV , Mukherjee MS , Lele SS . A study on genetic test of lactase persistence in relation to milk consumption in regional groups of India. Genet Test Mol Biomarkers. 2012;16(12):1413–1418. doi: 10.1089/gtmb.2012.0191. 23030683

[22] Goodrich JK , Davenport ER , Beaumont M , Jackson MA , Knight R , Ober C , Spector TD , Bell JT , Clark AG , Ley RE . Genetic determinants of the gut microbiome in UK twins. Cell Host Microbe. 2016;19(5):731–743. doi: 10.1016/j.chom.2016.04.017. 27173935 PMC4915943

[23] Sen CT . Food Culture in India. Bloomsbury Publishing USA; 2004.

[24] Shetty SA , Marathe NP , Shouche YS . Opportunities and challenges for gut microbiome studies in the Indian population. Microbiome. 2013;1(1):24. doi: 10.1186/2049-2618-1-24. 24451035 PMC3971629

[25] Abdill RJ , Adamowicz EM , Blekhman R . Public human microbiome data are dominated by highly developed countries. PLoS Biol. 2022;20(2):e3001536. doi: 10.1371/journal.pbio.3001536. 35167588 PMC8846514

[26] Bhute S , Pande P , Shetty SA , Shelar R , Mane S , Kumbhare SV , Gawali A , Makhani H , Navandar M , Dhotre D , et al. Molecular characterization and meta-analysis of gut microbial communities illustrate enrichment of prevotella and megasphaera in Indian subjects. Front Microbiol. 2016;7:660. doi: 10.3389/fmicb.2016.00660. 27242691 PMC4860526

[27] Dehingia M , Thangjam devi K , Talukdar NC , Talukdar R , Reddy N , Mande SS , Deka M , Khan MR . Gut bacterial diversity of the tribes of India and comparison with the worldwide data. NatSR. 2015;5:18563. doi: 10.1038/srep18563. PMC4686986 26689136

[28] Dhakan DB , Maji A , Sharma AK , Saxena R , Pulikkan J , Grace T , Gomez A , Scaria J , Amato KR , Sharma VK . The unique composition of Indian gut microbiome, gene catalogue, and associated fecal metabolome deciphered using multi-omics approaches. Gigascience. 2019;8(3):giz004. doi: 10.1093/gigascience/giz004. 30698687 PMC6394208

[29] Kaur K , Khatri I , Akhtar A , Subramanian S , Ramya TNC . Metagenomics analysis reveals features unique to Indian distal gut microbiota. PLoS One. 2020;15(4):e0231197. doi: 10.1371/journal.pone.0231197. 32267865 PMC7141701

[30] Tandon D , Haque MMRS , Shaikh SPS , Dubey AK , Mande SS . A snapshot of gut microbiota of an adult urban population from Western region of India. PLoS One. 2018;13(4):e0195643. doi: 10.1371/journal.pone.0195643. 29624599 PMC5889170

[31] Blanco-Míguez A , Gálvez EJC , Pasolli E , De Filippis F , Amend L , Huang KD , Manghi P , Lesker T-R , Riedel T , Cova L , et al. Extension of the *segatella copri* complex to 13 species with distinct large extrachromosomal elements and associations with host conditions. Cell Host Microbe. 2023;31(11):1804–1819.e9. doi: 10.1016/j.chom.2023.09.013. 37883976 PMC10635906

[32] Hitch TCA , Bisdorf K , Afrizal A , Riedel T , Overmann J , Strowig T , Clavel T . A taxonomic note on the genus prevotella: description of four novel genera and emended description of the genera hallella and xylanibacter. Syst Appl Microbiol. 2022;45(6):126354. doi: 10.1016/j.syapm.2022.126354. 36067550

[33] Chaudhari DS , Dhotre DP , Agarwal DM , Gaike AH , Bhalerao D , Jadhav P , Mongad D , Lubree H , Sinkar VP , Patil UK , et al. Gut, oral and skin microbiome of Indian patrilineal families reveal perceptible association with age. NatSR. 2020;10(1):5685. doi: 10.1038/s41598-020-62195-5. PMC7105498 32231240

[34] Das B , Ghosh TS , Kedia S , Rampal R , Saxena S , Bag S , Mitra R , Dayal M , Mehta O , Surendranath A , et al. Analysis of the gut microbiome of rural and urban healthy Indians living in sea level and high altitude areas. NatSR. 2018;8(1). doi: 10.1038/s41598-018-28550-3. PMC6031670 29973712

[35] Dubey AK , Uppadhyaya N , Nilawe P , Chauhan N , Kumar S , Gupta UA , Bhaduri A . LogMPIE, pan-India profiling of the human gut microbiome using 16S rRNA sequencing. Sci Data. 2018;5:180232. doi: 10.1038/sdata.2018.232. 30375992 PMC6207063

[36] Kulkarni AS , Kumbhare SV , Dhotre DP , Shouche YS . Mining the core gut microbiome from a sample Indian population. Indian J Microbiol. 2019;59(1):90–95. doi: 10.1007/s12088-018-0742-0. 30728635 PMC6328410

[37] Singh R , Haque MM , Mande SS . Lifestyle-induced microbial gradients: an Indian perspective. Front Microbiol. 2019;10:2874. doi: 10.3389/fmicb.2019.02874. 31921052 PMC6928055

[38] De Filippo C , Cavalieri D , Di Paola M , Ramazzotti M , Poullet JB , Massart S , Collini S , Pieraccini G , Lionetti P . Impact of diet in shaping gut microbiota revealed by a comparative study in children from Europe and rural Africa. Proc Natl Acad Sci. 2010;107(33):14691–14696. doi: 10.1073/pnas.1005963107. 20679230 PMC2930426

[39] Fragiadakis GK , Smits SA , Sonnenburg ED , Van Treuren W , Reid G , Knight R , Manjurano A , Changalucha J , Dominguez-Bello MG , Leach J , et al. Links between environment, diet, and the hunter-gatherer microbiome. Gut Microbes. 2019;10(2):216–227. doi: 10.1080/19490976.2018.1494103. 30118385 PMC6546328

[40] Gellman RH , Olm MR , Terrapon N , Enam F , Higginbottom SK , Sonnenburg JL , Sonnenburg ED . Hadza prevotella require diet-derived microbiota-accessible carbohydrates to persist in mice. Cell Rep. 2023;42(11):113233. doi: 10.1016/j.celrep.2023.113233. 38510311 PMC10954246

[41] Jha AR , Davenport ER , Gautam Y , Bhandari D , Tandukar S , Ng KM , Fragiadakis GK , Holmes S , Gautam GP , Leach J , et al. Gut microbiome transition across a lifestyle gradient in himalaya. PLoS Biol. 2018;16(11):e2005396. doi: 10.1371/journal.pbio.2005396. 30439937 PMC6237292

[42] Kaplan RC , Wang Z , Usyk M , Sotres-Alvarez D , Daviglus ML , Schneiderman N , Talavera GA , Gellman MD , Thyagarajan B , Moon J-Y , et al. Gut microbiome composition in the hispanic community health Study/Study of latinos is shaped by geographic relocation, environmental factors, and obesity. Genome Biol. 2019;20(1):219. doi: 10.1186/s13059-019-1831-z. 31672155 PMC6824043

[43] Tett A , Huang KD , Asnicar F , Fehlner-Peach H , Pasolli E , Karcher N , Armanini F , Manghi P , Bonham K , Zolfo M , et al. The prevotella copri complex comprises four distinct clades underrepresented in westernized populations. Cell Host Microbe. 2019;26(5):666–679.e7. doi: 10.1016/j.chom.2019.08.018. 31607556 PMC6854460

[44] Vangay P , Johnson AJ , Ward TL , Al-Ghalith GA , Shields-Cutler RR , Hillmann BM , Lucas SK , Beura LK , Thompson EA , Till LM , et al. US immigration westernizes the human gut microbiome. Cell. 2018;175(4):962–972.e10. doi: 10.1016/j.cell.2018.10.029. 30388453 PMC6498444

[45] Hazarika P , Chattopadhyay I , Umpo M , Choudhury Y , Sharma I . Elucidating the gut microbiome alterations of tribal community of arunachal pradesh: perspectives on their lifestyle or food habits. NatSR. 2022;12(1). doi: 10.1038/s41598-022-23124-w. PMC9622709 36316382

[46] Chakraborty S , Mollick SA , Roy B . Gut bacterial diversity in tribes of India: a review. Quant J Med Health Sci. 2024;3(4).

[47] Callahan BJ , Sankaran K , Fukuyama JA , McMurdie PJ , Holmes SP . Bioconductor workflow for microbiome data analysis: from raw reads to community analyses. F1000Res. 2016;5:1492. doi: 10.12688/f1000research.8986.2. 27508062 PMC4955027

[48] Olm MR , Crits-Christoph A , Bouma-Gregson K , Firek BA , Morowitz MJ , Banfield JF . inStrain profiles population microdiversity from metagenomic data and sensitively detects shared microbial strains. NatBi. 2021;39(6):727–736. doi: 10.1038/s41587-020-00797-0. PMC9223867 33462508

[49] Sakamoto M , Benno Y . Reclassification of bacteroides distasonis, bacteroides goldsteinii and bacteroides merdae as parabacteroides distasonis gen. Nov., comb. Nov., parabacteroides goldsteinii comb. Nov. and parabacteroides merdae comb. Nov. Int J Syst Evol Microbiol. 2006;56(Pt 7):1599–1605. doi: 10.1099/ijs.0.64192-0. 16825636

[50] García-López M , Meier-Kolthoff JP , Tindall BJ , Gronow S , Woyke T , Kyrpides NC , Hahnke RL , Göker M . Analysis of 1,000 type-strain genomes improves taxonomic classification of bacteroidetes. Front Microbiol. 2019;10:2083. doi: 10.3389/fmicb.2019.02083. 31608019 PMC6767994

[51] Chen L , Wang D , Garmaeva S , Kurilshikov A , Vila AV , Gacesa R , Sinha T , Segal E , Weersma RK , Wijmenga C , et al. The long-term genetic stability and individual specificity of the human gut microbiome. Cell. 2021;184(9):2302–2315.e12. doi: 10.1016/j.cell.2021.03.024. 33838112

[52] Neu AT , Allen EE , Roy K . Defining and quantifying the core microbiome: challenges and prospects. PNAS. 2021;118(51):e2104429118. doi: 10.1073/pnas.2104429118. 34862327 PMC8713806

[53] Sharon I , Quijada NM , Pasolli E , Fabbrini M , Vitali F , Agamennone V , Dötsch A , Selberherr E , Grau JH , Meixner M , et al. The core human microbiome: does it exist and how can we find it? A critical review of the concept. Nutrients. 2022;14(14):2872. doi: 10.3390/nu14142872. 35889831 PMC9323970

[54] Rosero JA , Killer J , Sechovcová H , Mrázek J , Benada O , Fliegerová K , Havlík J , Kopečný J . Reclassification of eubacterium rectale (Hauduroy et al. 1937) Prévot 1938 in a new genus agathobacter gen. Nov. as agathobacter rectalis comb. Nov., and description of agathobacter ruminis sp. Nov., isolated from the rumen contents of sheep and cows. Int J Syst Evol Microbiol. 2016;66(2):768–773. doi: 10.1099/ijsem.0.000788. 26619944

[55] Pulipati P , Sarkar P , Jakkampudi A , Kaila V , Sarkar S , Unnisa M , Reddy DN , Khan M , Talukdar R . The Indian gut microbiota—Is it unique? Indian J Gastroenterol. 2020;39(2):133–140. doi: 10.1007/s12664-020-01037-8. 32388710

[56] Prasoodanan PKV , Sharma AK , Mahajan S , Dhakan DB , Maji A , Scaria J , Sharma VK . Western and non-Western gut microbiomes reveal new roles of prevotella in carbohydrate metabolism and mouth–gut axis. NPJ Biofilms Microbiomes. 2021;7(1):1–17. doi: 10.1038/s41522-021-00248-x. 34620880 PMC8497558

[57] Pinna NK , Anjana RM , Saxena S , Dutta A , Gnanaprakash V , Rameshkumar G , Aswath S , Raghavan S , Rani CSS , Radha V , et al. Trans-ethnic gut microbial signatures of prediabetic subjects from India and Denmark. Genome Med. 2021;13(1):36. doi: 10.1186/s13073-021-00851-9. 33658065 PMC7931552

[58] Miquel S , Martín R , Rossi O , Bermúdez-Humarán L , Chatel J , Sokol H , Thomas M , Wells J , Langella P . *Faecalibacterium prausnitzii* and human intestinal health. Curr Opin Microbiol Ecol Indus Microbiol • Special Section: Innate Immunity. 2013;16(3):255–261. doi: 10.1016/j.mib.2013.06.003. 23831042

[59] Almeida A , Nayfach S , Boland M , Strozzi F , Beracochea M , Shi ZJ , Pollard KS , Sakharova E , Parks DH , Hugenholtz P , et al. A unified catalog of 204,938 reference genomes from the human gut microbiome. NatBi. 2020;39:1–10. doi: 10.1038/s41587-020-0603-3. PMC7801254 32690973

[60] Jena R , Choudhury PK . Bifidobacteria in fermented dairy foods: a health beneficial outlook. Prob Antimicrob Prot. 2023;17:1–22. doi: 10.1007/s12602-023-10189-w. 37979040

[61] Orihara K , Yahagi K , Saito Y , Watanabe Y , Sasai T , Hara T , Tsukuda N , Oki K , Fujimoto J , Matsuki T . Characterization of bifidobacterium kashiwanohense that utilizes both milk- and plant-derived oligosaccharides. Gut Microbes. 2023;15(1):2207455. doi: 10.1080/19490976.2023.2207455. 37188713 PMC10187079

[62] Gamage HKAH , Tetu SG , Chong RWW , Ashton J , Packer NH , Paulsen IT . Cereal products derived from wheat, sorghum, rice and oats alter the infant gut microbiota in vitro. NatSR. 2017;7(1):14312. doi: 10.1038/s41598-017-14707-z. PMC5662621 29085002

[63] Singh RK , Chang H-W , Yan D , Lee KM , Ucmak D , Wong K , Abrouk M , Farahnik B , Nakamura M , Zhu TH , et al. Influence of diet on the gut microbiome and implications for human health. J Transl Med. 2017;15(1):73. doi: 10.1186/s12967-017-1175-y. 28388917 PMC5385025

[64] Mallick H , Rahnavard A , McIver LJ , Ma S , Zhang Y , Nguyen LH , Tickle TL , Weingart G , Ren B , Schwager EH , et al. Multivariable association discovery in population-scale meta-omics studies. PLoS Comput Biol. 2021;17(11):e1009442. doi: 10.1371/journal.pcbi.1009442. 34784344 PMC8714082

[65] Zheng J , Wittouck S , Salvetti E , Franz CMAP , Harris HMB , Mattarelli P , O’Toole PW , Pot B , Vandamme P , Walter J , et al. A taxonomic note on the genus lactobacillus: description of 23 novel genera, emended description of the genus lactobacillus beijerinck 1901, and union of lactobacillaceae and leuconostocaceae. Int J Syst Evol Microbiol. 2020;70(4):2782–2858. doi: 10.1099/ijsem.0.004107. 32293557

[66] Volokh O , Klimenko N , Berezhnaya Y , Tyakht A , Nesterova P , Popenko A , Alexeev D . Human gut microbiome response induced by fermented dairy product intake in healthy volunteers. Nutrients. 2019;11(3):547. doi: 10.3390/nu11030547. 30836671 PMC6470569

[67] Yan S , Huang P , Yu L , Tian F , Zhao J , Chen W , Zhai Q . Metabolomic analysis reveals *Ligilactobacillus salivarius* CCFM 1266 fermentation improves dairy product quality. Food Res Int. 2024;188:114309. doi: 10.1016/j.foodres.2024.114309. 38823823

[68] Arola H , Tamm A . Metabolism of lactose in the human body. Scand J Gastroenterol. 1994;29(sup202):21–25. doi: 10.3109/00365529409091741. 8042015

[69] Palframan RJ , Gibson GR , Rastall RA , Vriers D . Carbohydrate preferences of bifidobacterium species-isolated from the human gut. Curr Issues Intest Microbiol. 2003;4(2):71–75. https://pubmed.ncbi.nlm.nih.gov/14503691/. 14503691

[70] González-Rodríguez I , Gaspar P , Sánchez B , Gueimonde M , Margolles A , Neves AR . Catabolism of glucose and lactose in bifidobacterium animalis subsp. Lactis, studied by 13C nuclear magnetic resonance. Appl Environ Microbiol (work). 2013;79:7628–7638. doi: 10.1128/AEM.02529-13. PMC3837816 24077711

[71] Parche S , Beleut M , Rezzonico E , Jacobs D , Arigoni F , Titgemeyer F , Jankovic I . Lactose-over-glucose preference in bifidobacterium longum NCC2705: glcP, encoding a glucose transporter, is subject to lactose repression. J Bacteriol. 2006;188(4):1260–1265. doi: 10.1128/jb.188.4.1260-1265.2006. 16452407 PMC1367232

[72] Franzosa EA , McIver LJ , Rahnavard G , Thompson LR , Schirmer M , Weingart G , Lipson KS , Knight R , Caporaso JG , Segata N , et al. Species-level functional profiling of metagenomes and metatranscriptomes. Nat Methods. 2018;15(11):962–968. doi: 10.1038/s41592-018-0176-y. 30377376 PMC6235447

[73] Pokusaeva K , Fitzgerald GF , van Sinderen D . Carbohydrate metabolism in bifidobacteria. Genes Nutr. 2011;6(3):285–306. doi: 10.1007/s12263-010-0206-6. 21484167 PMC3145055

[74] Leser T , Baker A . Bifidobacterium adolescentis—A beneficial microbe. Benef Microbes. 2023;14(6):525–551. 10.1163/18762891-20230030. 38350464

[75] Duranti S , Milani C , Lugli GA , Mancabelli L , Turroni F , Ferrario C , Mangifesta M , Viappiani A , Sánchez B , Margolles A , et al. Evaluation of genetic diversity among strains of the human gut commensal bifidobacterium adolescentis. NatSR. 2016;6(1):23971. doi: 10.1038/srep23971. PMC4817515 27035119

[76] Kim CY , Lee M , Yang S , Kim K , Yong D , Kim HR , Lee I . Human reference gut microbiome catalog including newly assembled genomes from under-represented asian metagenomes. Genome Med. 2021;13(1):134. doi: 10.1186/s13073-021-00950-7. 34446072 PMC8394144

[77] Schnorr SL , Candela M , Rampelli S , Centanni M , Consolandi C , Basaglia G , Turroni S , Biagi E , Peano C , Severgnini M , et al. Gut microbiome of the hadza hunter-gatherers. Nat Commun. 2014;5(1):3654. doi: 10.1038/ncomms4654. 24736369 PMC3996546

[78] Murrell B , Weaver S , Smith MD , Wertheim JO , Murrell S , Aylward A , Eren K , Pollner T , Martin DP , Smith DM , et al. Gene-wide identification of episodic selection. Mol Biol Evol. 2015;32(5):1365–1371. doi: 10.1093/molbev/msv035. 25701167 PMC4408417

[79] Drula E , Garron M-L , Dogan S , Lombard V , Henrissat B , Terrapon N . The carbohydrate-active enzyme database: functions and literature. Nucleic Acids Res. 2022;50(D1):D571–D577. doi: 10.1093/nar/gkab1045. 34850161 PMC8728194

[80] Ausland C , Zheng J , Yi H , Yang B , Li T , Feng X , Zheng B , Yin Y . dbCAN-PUL: a database of experimentally characterized CAZyme gene clusters and their substrates. Nucleic Acids Res. 2021;49(D1):D523–D528. doi: 10.1093/nar/gkaa742. 32941621 PMC7778981

[81] Koropatkin NM , Cameron EA , Martens EC . How glycan metabolism shapes the human gut microbiota. Nat Rev Microbiol. 2012;10(5):323–335. doi: 10.1038/nrmicro2746. 22491358 PMC4005082

[82] La Rosa SL , Ostrowski MP , Vera-Ponce de León A , McKee LS , Larsbrink J , Eijsink VG , Lowe EC , Martens EC , Pope PB . Glycan processing in gut microbiomes. Curr Opin Microbiol. 2022;67:102143. doi: 10.1016/j.mib.2022.102143. 35338908

[83] Wardman JF , Bains RK , Rahfeld P , Withers SG . Carbohydrate-active enzymes (CAZymes) in the gut microbiome. Nat Rev Microbiol. 2022;20(9):542–556. doi: 10.1038/s41579-022-00712-1. 35347288

[84] Smits SA , Leach J , Sonnenburg ED , Gonzalez CG , Lichtman JS , Reid G , Knight R , Manjurano A , Changalucha J , Elias JE , et al. Seasonal cycling in the gut microbiome of the hadza hunter-gatherers of Tanzania. Science. 2017;357(6353):802–806. doi: 10.1126/science.aan4834. 28839072 PMC5891123

[85] Carter MM , Olm MR , Merrill BD , Dahan D , Tripathi S , Spencer SP , Yu FB , Jain S , Neff N , Jha AR , et al. Ultra-deep sequencing of hadza hunter-gatherers recovers vanishing gut microbes. Cell. 2023;186(14):3111–3124.e13. doi: 10.1016/j.cell.2023.05.046. 37348505 PMC10330870

[86] Bäckhed F . Host responses to the human microbiome. Nutr Rev. 2012;70(Suppl 1):S14–17. doi: 10.1111/j.1753-4887.2012.00496.x. 22861802

[87] Andreu-Sánchez S , Blanco-Míguez A , Wang D , Golzato D , Manghi P , Heidrich V , Fackelmann G , Zhernakova DV , Kurilshikov A , Valles-Colomer M , et al. Global genetic diversity of human gut microbiome species is related to geographic location and host health. Cell. 2025;S0092-8674(25):00416–00417. doi: 10.1016/j.cell.2025.04.014. 40311618

[88] Yatsunenko T , Rey FE , Manary MJ , Trehan I , Dominguez-Bello MG , Contreras M , Magris M , Hidalgo G , Baldassano RN , Anokhin AP , et al. Human gut microbiome viewed across age and geography. Nature. 2012;486(7402):222–227. doi: 10.1038/nature11053. 22699611 PMC3376388

[89] Bulliyya , Karr . Diet and nutritional issues of scheduled tribes and primitive tribal communities in India. In AB Ota (Ed.), Critical Issues in Tribal Development. 2009. p. 248–262 Scheduled Castes & Scheduled Tribes Research and Training Institute.

[90] Mosites E , Sammons M , Otiang E , Eng A , Noecker C , Manor O , Hilton S , Thumbi SM , Onyango C , Garland-Lewis G , et al. Microbiome sharing between children, livestock and household surfaces in Western Kenya. PLoS One. 2017;12(2):e0171017. doi: 10.1371/journal.pone.0171017. 28152044 PMC5289499

[91] Hu Y , Yang X , Li J , Lv N , Liu F , Wu J , Lin IYC , Wu N , Weimer BC , Gao GF , et al. The bacterial mobile resistome transfer network connecting the animal and human microbiomes. ApEnM. 2016;82(22):6672–6681. doi: 10.1128/AEM.01802-16. PMC5086561 27613679

[92] Levy SB , Fitzgerald GB , Macone AB . Spread of antibiotic-resistant plasmids from chicken to chicken and from chicken to man. Nature. 1976;260(5546):40–42. doi: 10.1038/260040a0. 772441

[93] Sun J , Liao X-P , D’Souza AW , Boolchandani M , Li S-H , Cheng K , Luis Martínez J , Li L , Feng Y-J , Fang L-X , et al. Environmental remodeling of human gut microbiota and antibiotic resistome in livestock farms. Nat Commun. 2020;11(1):1427. doi: 10.1038/s41467-020-15222-y. 32188862 PMC7080799

[94] Yersin S , Garneau JR , Schneeberger PHH , Osman KA , Cercamondi CI , Muhummed AM , Tschopp R , Zinsstag J , Vonaesch P . Gut microbiomes of agropastoral children from the adadle region of Ethiopia reflect their unique dietary habits. NatSR. 2023;13(1). doi: 10.1038/s41598-023-47748-8. PMC10696028 38049420

[95] Suzuki TA , Fitzstevens JL , Schmidt VT , Enav H , Huus KE , Mbong Ngwese M , Grießhammer A , Pfleiderer A , Adegbite BR , Zinsou JF , et al. Codiversification of gut microbiota with humans. Science. 2022;377(6612):1328–1332. doi: 10.1126/science.abm7759. 36108023 PMC10777373

[96] Arboleya S , Watkins C , Stanton C , Ross RP . Gut bifidobacteria populations in human health and aging. Front Microbiol. 2016;7. doi: 10.3389/fmicb.2016.01204. PMC4990546 27594848

[97] Berhe T , Ipsen R , Seifu E , Kurtu MY , Fugl A , Hansen EB . Metagenomic analysis of bacterial community composition in dhanaan: Ethiopian traditional fermented camel milk. FEMS Microbiol Lett. 2019;366(11):fnz128. doi: 10.1093/femsle/fnz128. 31183493

[98] de Melo Pereira GV , de Carvalho Neto DP , Maske BL , De Dea Lindner J , Vale AS , Favero GR , Viesser J , de Carvalho JC , Góes-Neto A , Soccol CR . An updated review on bacterial community composition of traditional fermented milk products: what next-generation sequencing has revealed so far? Crit Rev Food Sci Nutr. 2022;62(7):1870–1889. doi: 10.1080/10408398.2020.1848787. 33207956

[99] Derrien M , Vlieg JET , van H . Fate, activity, and impact of ingested bacteria within the human gut microbiota. TIM. 2015;23(6):354–366. doi: 10.1016/j.tim.2015.03.002. 25840765

[100] Lamoureux L , Roy D , Gauthier SF . Production of oligosaccharides in yogurt containing bifidobacteria and yogurt cultures. J Dairy Sci. 2002;85(5):1058–1069. doi: 10.3168/jds.S0022-0302(02)74166-0. 12086039

[101] Evershed RP , Davey Smith G , Roffet-Salque M , Timpson A , Diekmann Y , Lyon MS , Cramp LJE , Casanova E , Smyth J , Whelton HL , et al. Dairying, diseases and the evolution of lactase persistence in Europe. Nature. 2022;608(7922):336–345. doi: 10.1038/s41586-022-05010-7. 35896751 PMC7615474

[102] Itan Y , Jones BL , Ingram CJ , Swallow DM , Thomas MG . A worldwide correlation of lactase persistence phenotype and genotypes. BMC Evol Biol. 2010;10(1):36. doi: 10.1186/1471-2148-10-36. 20144208 PMC2834688

[103] Sabeti PC , Schaffner SF , Fry B , Lohmueller J , Varilly P , Shamovsky O , Palma A , Mikkelsen TS , Altshuler D , Lander ES . Positive natural selection in the human lineage. Science. 2006;312(5780):1614–1620. doi: 10.1126/science.1124309. 16778047

[104] Babu J , Kumar S , Babu P , Prasad JH , Ghoshal UC . Frequency of lactose malabsorption among healthy Southern and Northern Indian populations by genetic analysis and lactose hydrogen breath and tolerance tests123. Am J Clin Nutr. 2010;91(1):140–146. doi: 10.3945/ajcn.2009.27946. 19889824

[105] Gallego Romero I , Basu Mallick C , Liebert A , Crivellaro F , Chaubey G , Itan Y , Metspalu M , Eaaswarkhanth M , Pitchappan R , Villems R , et al. Herders of Indian and european cattle share their predominant allele for lactase persistence. Mol Biol Evol. 2012;29(1):249–260. doi: 10.1093/molbev/msr190. 21836184

[106] Reddy V , Pershad J . Lactase deficiency in Indians. Am J Clin Nutr. 1972;25(1):114–119. doi: 10.1093/ajcn/25.1.114. 5066618

[107] Blekhman R , Goodrich JK , Huang K , Sun Q , Bukowski R , Bell JT , Spector TD , Keinan A , Ley RE , Gevers D , et al. Host genetic variation impacts microbiome composition across human body sites. Genome Biol. 2015;16(1):191. doi: 10.1186/s13059-015-0759-1. 26374288 PMC4570153

[108] Kato K , Ishida S , Tanaka M , Mitsuyama E , Xiao J , Odamaki T . Association between functional lactase variants and a high abundance of bifidobacterium in the gut of healthy Japanese people. PLoS One. 2018;13(10):e0206189. doi: 10.1371/journal.pone.0206189. 30339693 PMC6195297

[109] Azcarate-Peril MA , Ritter AJ , Savaiano D , Monteagudo-Mera A , Anderson C , Magness ST , Klaenhammer TR . Impact of short-chain galactooligosaccharides on the gut microbiome of lactose-intolerant individuals. Proc Natl Acad Sci. 2017;114(3):E367–E375. doi: 10.1073/pnas.1606722113. 28049818 PMC5255593

[110] Nayfach S , Shi ZJ , Seshadri R , Pollard KS , Kyrpides NC . New insights from uncultivated genomes of the global human gut microbiome. Nature. 2019;568(7753):505–510. doi: 10.1038/s41586-019-1058-x. 30867587 PMC6784871

[111] Pasolli E , Asnicar F , Manara S , Zolfo M , Karcher N , Armanini F , Beghini F , Manghi P , Tett A , Ghensi P , et al. Extensive unexplored human microbiome diversity revealed by over 150,000 genomes from metagenomes spanning age, geography, and lifestyle. Cell. 2019;176(3):649–662.e20. doi: 10.1016/j.cell.2019.01.001. 30661755 PMC6349461

[112] R Core Team . R: A language and environment for statistical computing. [Computer software]. 2022. https://www.R-project.org/

[113] Urbanek S , Horner J . _Cairo: R Graphics Device using Cairo Graphics Library for Creating High-Quality Bitmap (PNG, JPEG, TIFF), Vector (PDF, SVG, PostScript) and Display (X11 and Win32) Output_ (Version R package version 1.6-2) [Computer software]. 2023. https://CRAN.R-project.org/package=Cairo

[114] Klindworth A , Pruesse E , Schweer T , Peplies J , Quast C , Horn M , Glöckner FO . Evaluation of general 16S ribosomal RNA gene PCR primers for classical and next-generation sequencing-based diversity studies. Nucleic Acids Res. 2013;41(1):e1. doi: 10.1093/nar/gks808. 22933715 PMC3592464

[115] Quast C , Pruesse E , Yilmaz P , Gerken J , Schweer T , Yarza P , Peplies J , Glöckner FO . The SILVA ribosomal RNA gene database project: improved data processing and web-based tools. Nucleic Acids Res. 2013;41(D1):D590–D596. doi: 10.1093/nar/gks1219. 23193283 PMC3531112

[116] Bushnell B . BBTools software package [Computer software]. 2014. https://sourceforge.net/projects/bbmap.

[117] Nurk S , Meleshko D , Korobeynikov A , Pevzner PA . MetaSPAdes: a new versatile metagenomic assembler. Genome Res. 2017;27(5):824–834. doi: 10.1101/gr.213959.116. 28298430 PMC5411777

[118] Gurevich A , Saveliev V , Vyahhi N , Tesler G . QUAST: quality assessment tool for genome assemblies. Bioinformatics. 2013;29(8):1072–1075. doi: 10.1093/bioinformatics/btt086. 23422339 PMC3624806

[119] Langmead B , Salzberg SL . Fast gapped-read alignment with bowtie 2. Nat Methods. 2012;9(4):357–359. doi: 10.1038/nmeth.1923.​​​​ 22388286 PMC3322381

[120] Kang DD , Li F , Kirton E , Thomas A , Egan R , An H , Wang Z . MetaBAT 2: an adaptive binning algorithm for robust and efficient genome reconstruction from metagenome assemblies. PeerJ. 2019;7:e7359. doi: 10.7717/peerj.7359. 31388474 PMC6662567

[121] Parks DH , Imelfort M , Skennerton CT , Hugenholtz P , Tyson GW . CheckM: assessing the quality of microbial genomes recovered from isolates, single cells, and metagenomes. Genome Res. 2015;25(7):1043–1055. doi: 10.1101/gr.186072.114. 25977477 PMC4484387

[122] Chaumeil P-A , Mussig AJ , Hugenholtz P , Parks DH . GTDB-Tk: a toolkit to classify genomes with the genome taxonomy database. Bioinformatics. 2020;36(6):1925–1927. doi: 10.1093/bioinformatics/btz848. PMC7703759 31730192

[123] Bowers RM , Kyrpides NC , Stepanauskas R , Harmon-Smith M , Doud D , Reddy TBK , Schulz F , Jarett J , Rivers AR , Eloe-Fadrosh EA , et al. Minimum information about a single amplified genome (MISAG) and a metagenome-assembled genome (MIMAG) of bacteria and archaea. NatBi. 2017;35(8):725–731. doi: 10.1038/nbt.3893. PMC6436528 28787424

[124] Olm MR , Brown CT , Brooks B , Banfield JF . dRep: a tool for fast and accurate genomic comparisons that enables improved genome recovery from metagenomes through de-replication. ISME J. 2017;11(12):2864–2868. doi: 10.1038/ismej.2017.126. 28742071 PMC5702732

[125] Lee MD . GToTree: a user-friendly workflow for phylogenomics. Bioinformatics. 2019;35(20):4162–4164. doi: 10.1093/bioinformatics/btz188. 30865266 PMC6792077

[126] Aroney STN , Newell RJP , Nissen J , Camargo AP , Tyson GW , Woodcroft BJ . CoverM: read coverage calculator for metagenomics (Version 0.7.0). [Computer software]. 2024. doi: 10.5281/zenodo.10531253.

[127] Oksanen J , Blanchet FG , Friendly M , Kindt R , Legendre P , McGlinn D , Minchin PR , O’Hara RB , Simpson GL , Solymos P . Vegan: community ecology package. R package version. 2022;2:5–7. 2020.

[128] Dray S , Dufour A-B . The ade4 package: implementing the duality diagram for ecologists. J Stat Softw. 2007;22:1–20. doi: 10.18637/jss.v022.i04.

[129] Kassambara A , Mundt F (2020) Extract and Visualize the Results of Multivariate Data Analyses [R package factoextra version 1.0.7]. https://www.scinapse.io

[130] Wickham H . ggplot2: Elegant Graphics for Data Analysis. 2016. Springer-Verlag New York. https://ggplot2.tidyverse.org.

[131] Zhang H , Yohe T , Huang L , Entwistle S , Wu P , Yang Z , Busk PK , Xu Y , Yin Y. dbCAN2: a meta server for automated carbohydrate-active enzyme annotation. Nucleic Acids Research. 2018;46:W95–W101. doi: 10.1093/nar/gky418. 29771380 PMC6031026

[132] Marçais G , Delcher AL , Phillippy AM , Coston R , Salzberg SL , Zimin A . MUMmer4: a fast and versatile genome alignment system. PLoS Comput Biol. 2018;14(1):e1005944. doi: 10.1371/journal.pcbi.1005944. 29373581 PMC5802927

[133] Cabanettes F , Klopp C . D-GENIES: dot plot large genomes in an interactive, efficient and simple way. PeerJ. 2018;6:e4958. doi: 10.7717/peerj.4958. 29888139 PMC5991294

[134] Li H . Minimap2: pairwise alignment for nucleotide sequences. Bioinformatics. 2018;34(18):3094–3100. doi: 10.1093/bioinformatics/bty191. 29750242 PMC6137996

[135] Stamatakis A . RAxML version 8: a tool for phylogenetic analysis and post-analysis of large phylogenies. Bioinformatics. 2014;30(9):1312–1313. doi: 10.1093/bioinformatics/btu033. 24451623 PMC3998144

[136] Ladeira R , Tap J , Derrien M . Exploring bifidobacterium species community and functional variations with human gut microbiome structure and health beyond infancy. Microb Res Rep. 2023;2(2):9. doi: 10.20517/mrr.2023.01. PMC10688807 38047280

[137] Capella-Gutiérrez S , Silla-Martínez JM , Gabaldón T . trimAl: a tool for automated alignment trimming in large-scale phylogenetic analyses. Bioinformatics. 2009;25(15):1972–1973. doi: 10.1093/bioinformatics/btp348. 19505945 PMC2712344

[138] Eddy SR . Accelerated profile HMM searches. PLoS Comput Biol. 2011;7(10):e1002195. doi: 10.1371/journal.pcbi.1002195. 22039361 PMC3197634

[139] Edgar RC . Search and clustering orders of magnitude faster than BLAST. Bioinformatics. 2010;26(19):2460–2461. doi: 10.1093/bioinformatics/btq461. 20709691

[140] Hyatt D , LoCascio PF , Hauser LJ , Uberbacher EC . Gene and translation initiation site prediction in metagenomic sequences. Bioinformatics. 2012;28(17):2223–2230. doi: 10.1093/bioinformatics/bts429. 22796954

[141] Price MN , Dehal PS , Arkin AP . FastTree 2 – approximately maximum-likelihood trees for large alignments. PLoS One. 2010;5(3):e9490. doi: 10.1371/journal.pone.0009490. 20224823 PMC2835736

[142] Sievers F , Wilm A , Dineen D , Gibson TJ , Karplus K , Li W , Lopez R , McWilliam H , Remmert M , Söding J , et al. Fast, scalable generation of high-quality protein multiple sequence alignments using clustal omega. Mol Syst Biol. 2011;7:539. doi: 10.1038/msb.2011.75. 21988835 PMC3261699

[143] Weaver S , Shank SD , Spielman SJ , Li M , Muse SV , Kosakovsky Pond SL . Datamonkey 2.0: a modern web application for characterizing selective and other evolutionary processes. Mol Biol Evol. 2018;35(3):773–777. doi: 10.1093/molbev/msx335. 29301006 PMC5850112

[144] Kosakovsky Pond SL , Frost SDW . Not so different after all: a comparison of methods for detecting amino acid sites under selection. Mol Biol Evol. 2005;22(5):1208–1222. doi: 10.1093/molbev/msi105. 15703242

[145] Xaxa V . State, Society, and Tribes: Issues in Post-colonial India. Pearson Education India; 2008.

[146] Chophy GK . Contested tribes: the subjectivity of tribe and state in India. Public Humanities. 2025;1:e143. doi: 10.1017/pub.2025.10059.

[147] Sneath D . Tribe. The Open Encyclopedia of Anthropology. 2016. doi: 10.29164/16tribe.

[148] Srivastava VK . The National committee report on tribal People—Vinay kumar srivastava, 2018. Soc Change. 2018;48:120–130. doi: 10.1177/0049085717743843.

[149] Xaxa V . Report on the high level committee on socio-economic, health and educational status of tribal communities of India. 2014. http://archive.nyu.edu/handle/2451/36746.

[150] Béteille A . The concept of tribe with special reference to India. Eur J Sociol/Arc Eur Sociol. 1986;27(2):296–318. doi: 10.1017/S000397560000463X.

[151] Malhotra KC , Vasulu TS . Structure of human populations in India. In PP Majumder (Ed.), Human Population Genetics: A Centennial Tribute to J. B. S. Haldane 1993. p. 207–233. Springer US. doi: 10.1007/978-1-4615-2970-5_15.

[152] Reddy BM , Tripathy V , Kumar V , Alla N . Molecular genetic perspectives on the Indian social structure. Am J Hum Biol. 2010;22(3):410–417. doi: 10.1002/ajhb.20983. 19743302

[153] Majumder PP . People of India: biological diversity and affinities. Evolutionary Anthropology: Issues, News, and Reviews. 1998;6(3):100–110. doi: 10.1002/(SICI)1520-6505(1998)6:3<100:AID-EVAN4>3.0.CO;2-I.

[154] Tamang R , Chaubey G , Nandan A , Govindaraj P , Singh VK , Rai N , Mallick CB , Sharma V , Sharma VK , Shah AM , et al. Reconstructing the demographic history of the himalayan and adjoining populations. Hum Genet. 2018;137(2):129–139. doi: 10.1007/s00439-018-1867-2. 29356938

[155] Srivastava VK . Concept of ‘tribe’ in the Draft National tribal policy. 2008.

[156] Xaxa V . Tribes as indigenous people of India. Econ Polit Wkly. 1999;34(51):3589–3595.

