# Supplementary material for: Gut microbiomes of tribal communities in India vary with dairy and grain consumption
Source: Gut Microbes. 2026 Jul 9;18(1):2694242. doi: 10.1080/19490976.2026.2694242 (PMC13353789; doi:10.1080/19490976.2026.2694242)

Figure 1 Supplement 1

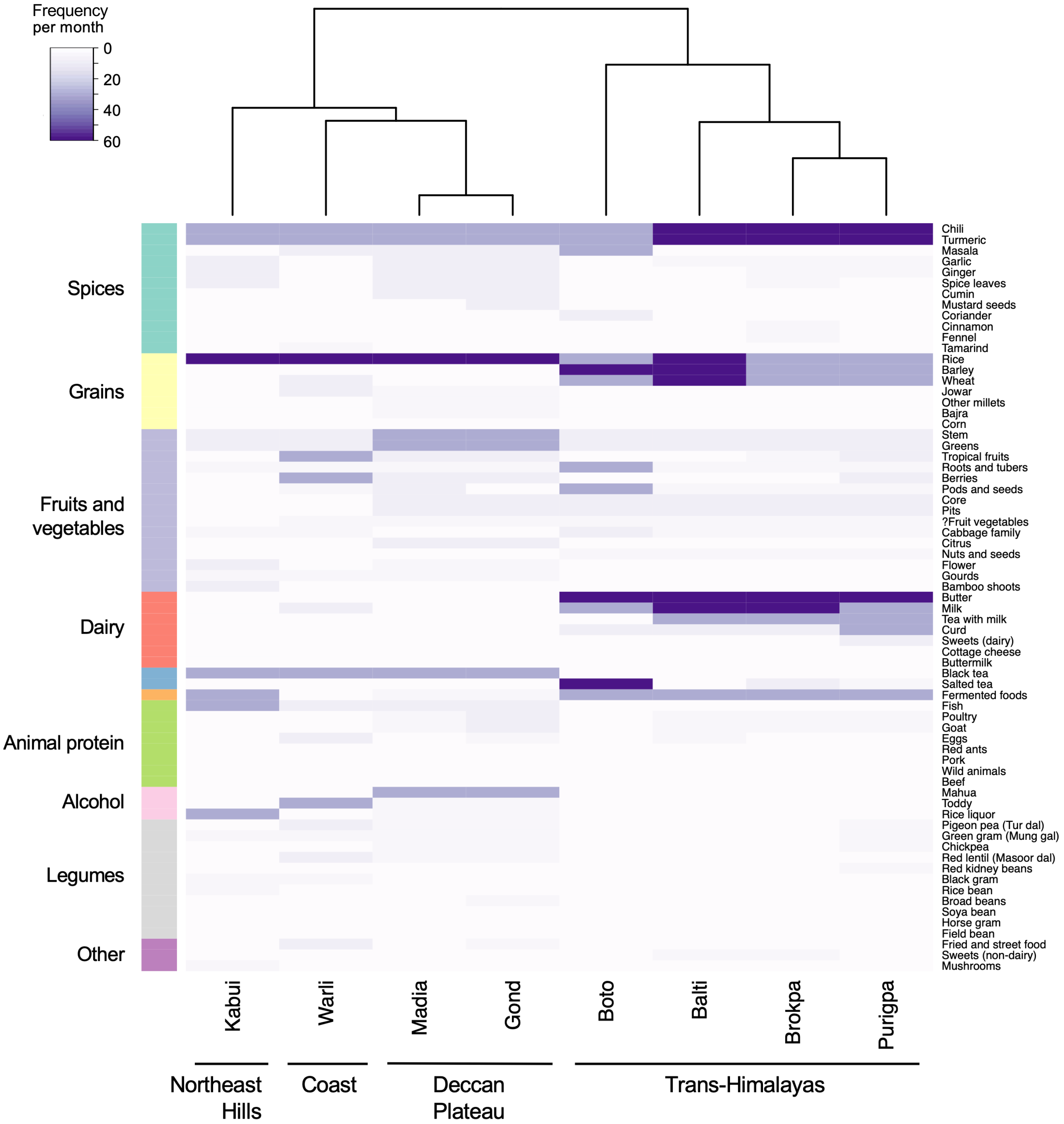

Figure 2 Supplement 1

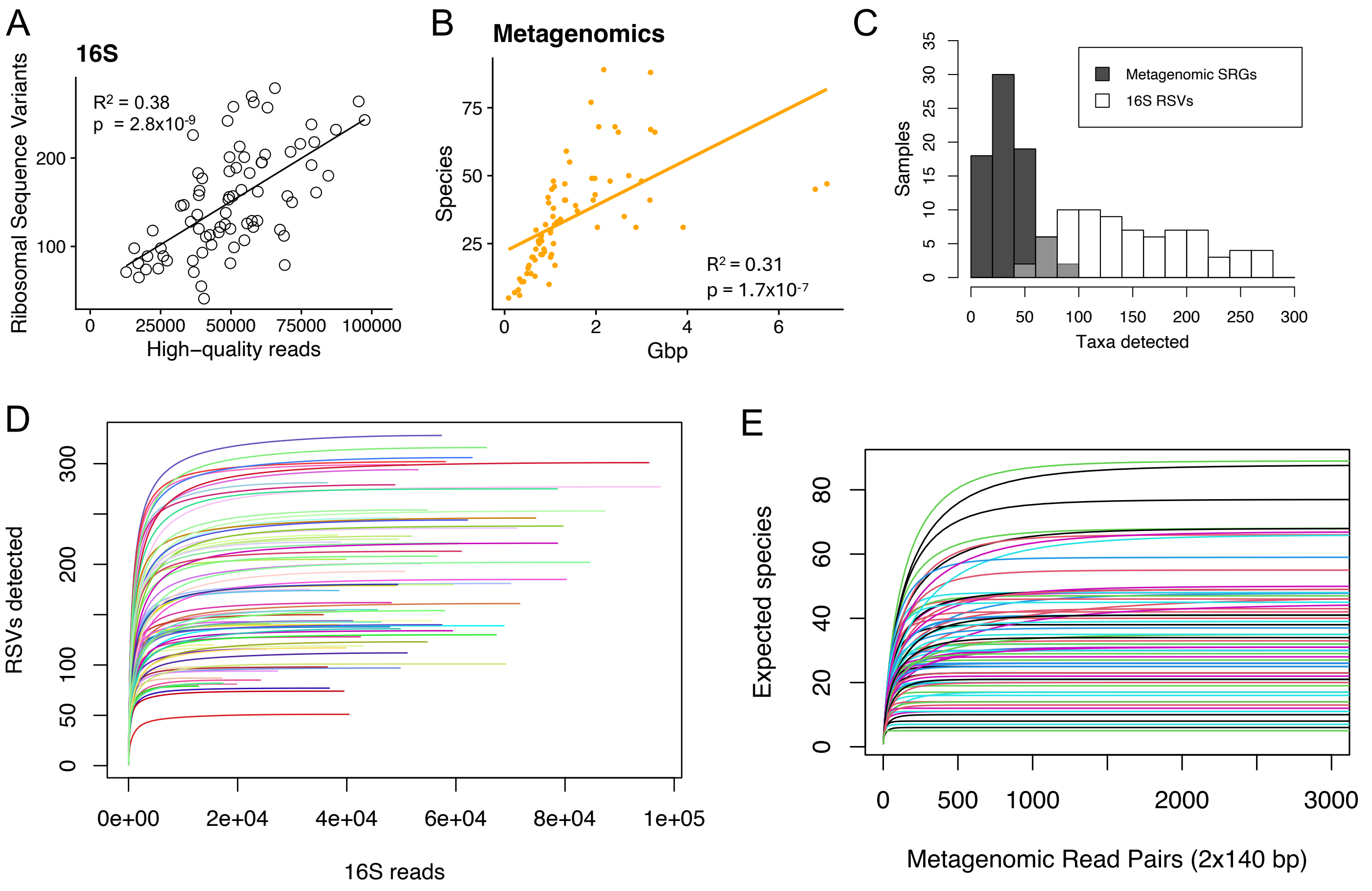

Figure 2 Supplement 2

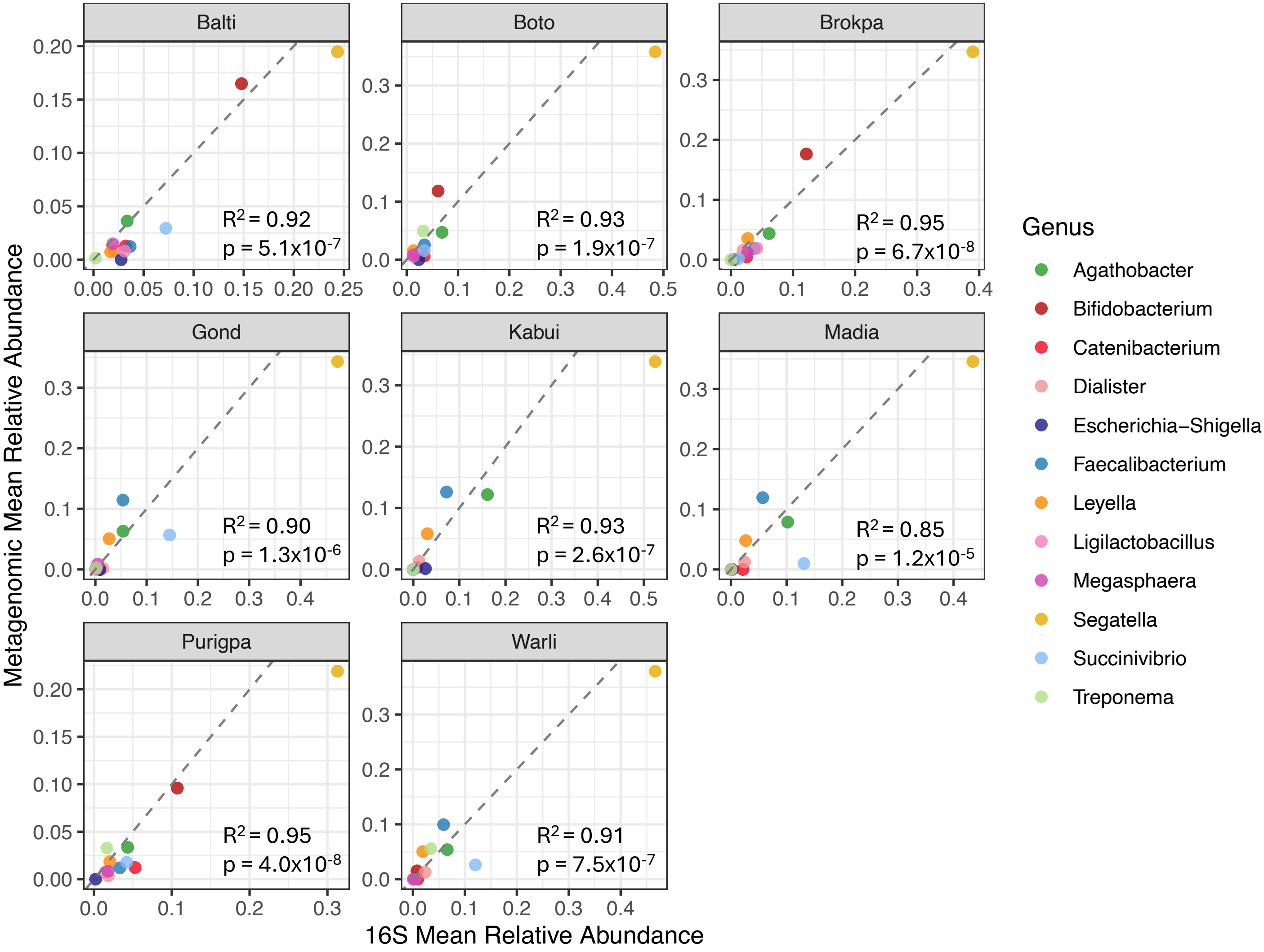

Figure 2 Supplement 3

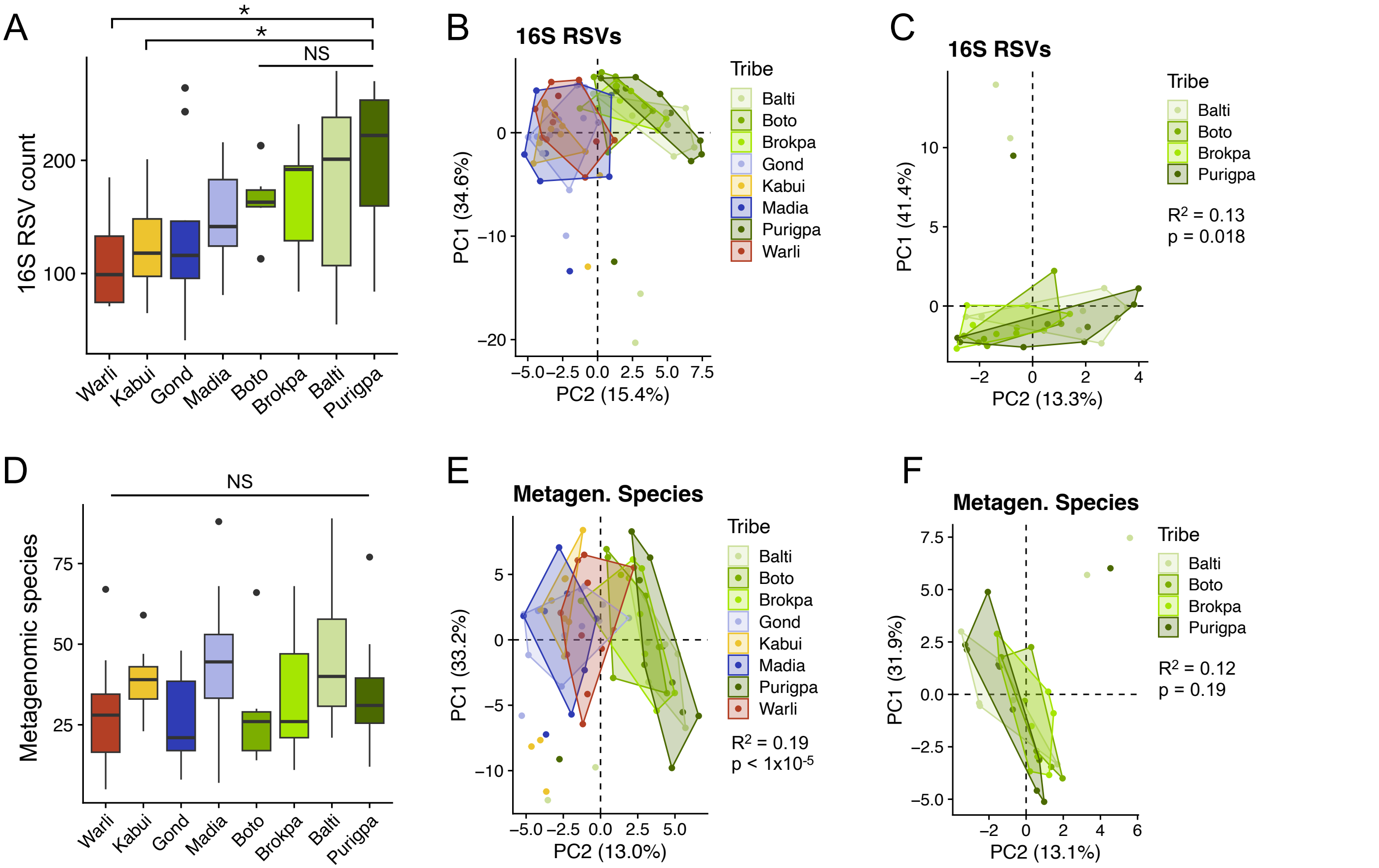

Figure 2 Supplement 4

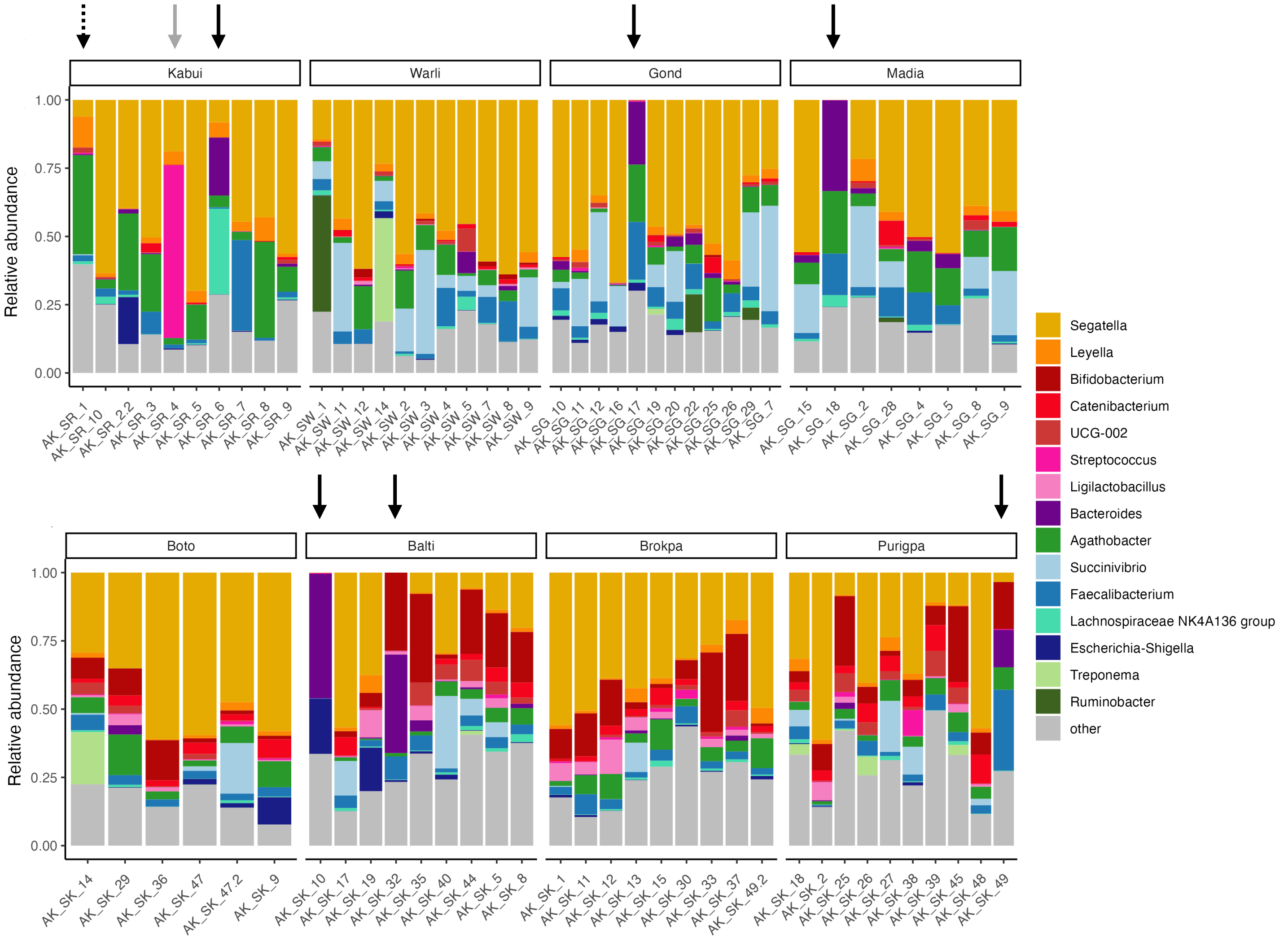

→ *Bacteroides/Phocaeicola/Parabacteroides* outlier, both 16S and metagenomics.

⇢ *Bacteroides/Phocaeicola/Parabacteroides* outlier, metagenomics only. AK_SR_2 failed 16S sequencing.

→ *Streptococcus* outlier

Figure 2 Supplement 5

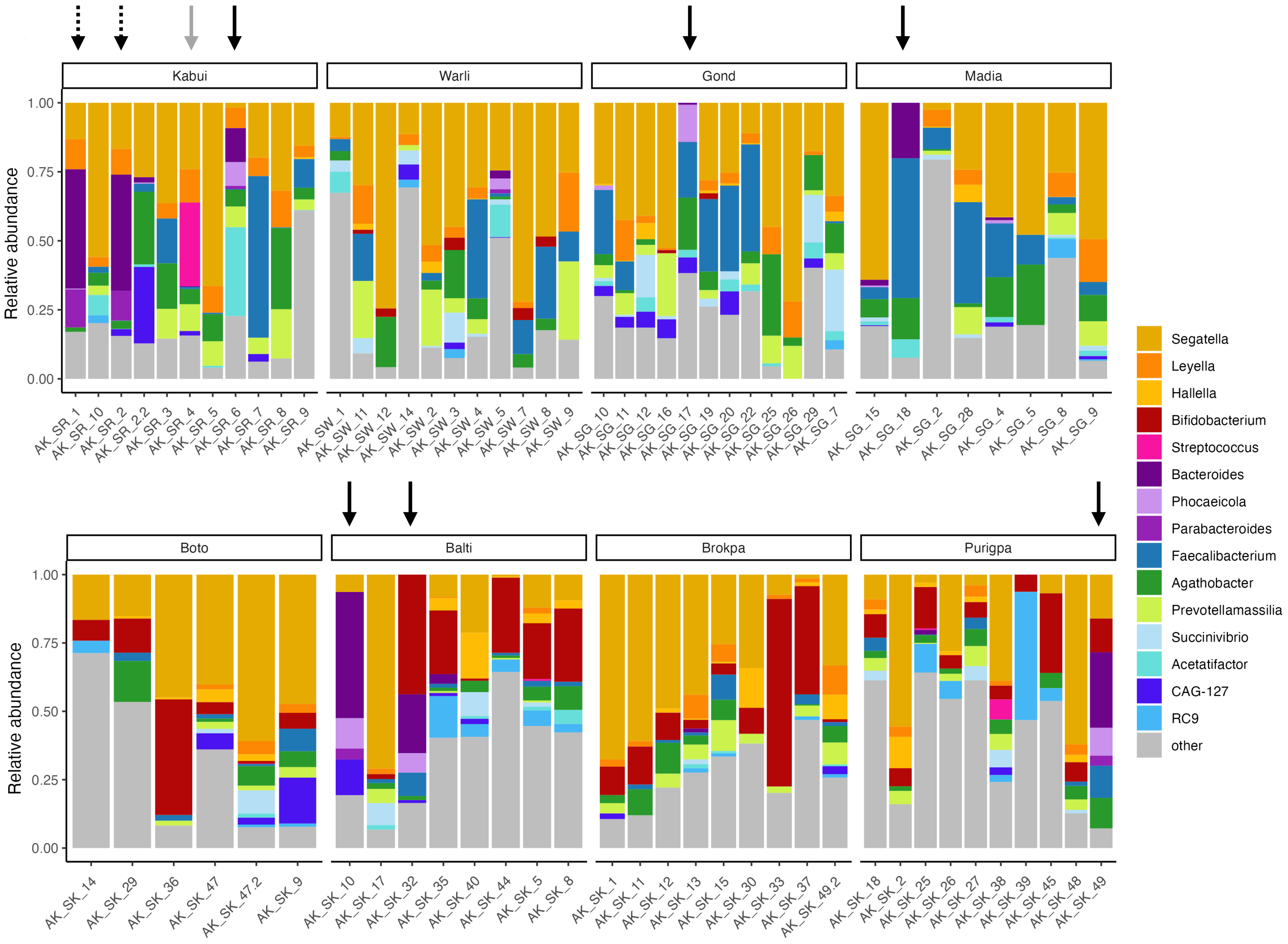

→ *Bacteroides/Phocaeicola/Parabacteroides* outlier, both 16S and metagenomics.

⋯→ *Bacteroides/Phocaeicola/Parabacteroides* outlier, metagenomics only. AK_SR_2 failed 16S sequencing.

→ *Streptococcus* outlier

Figure 2 Supplement 6

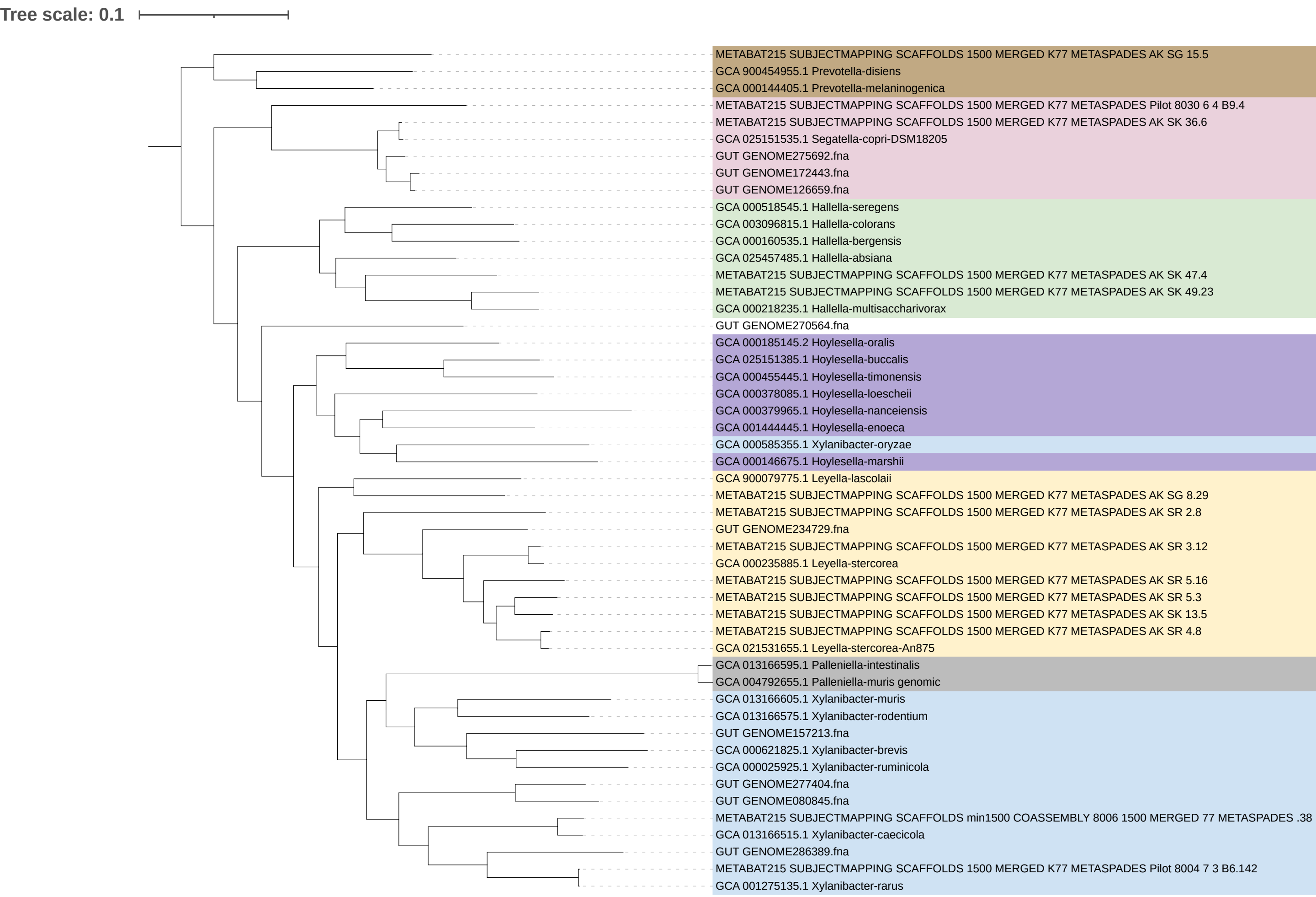

Figure 3 Supplement 1

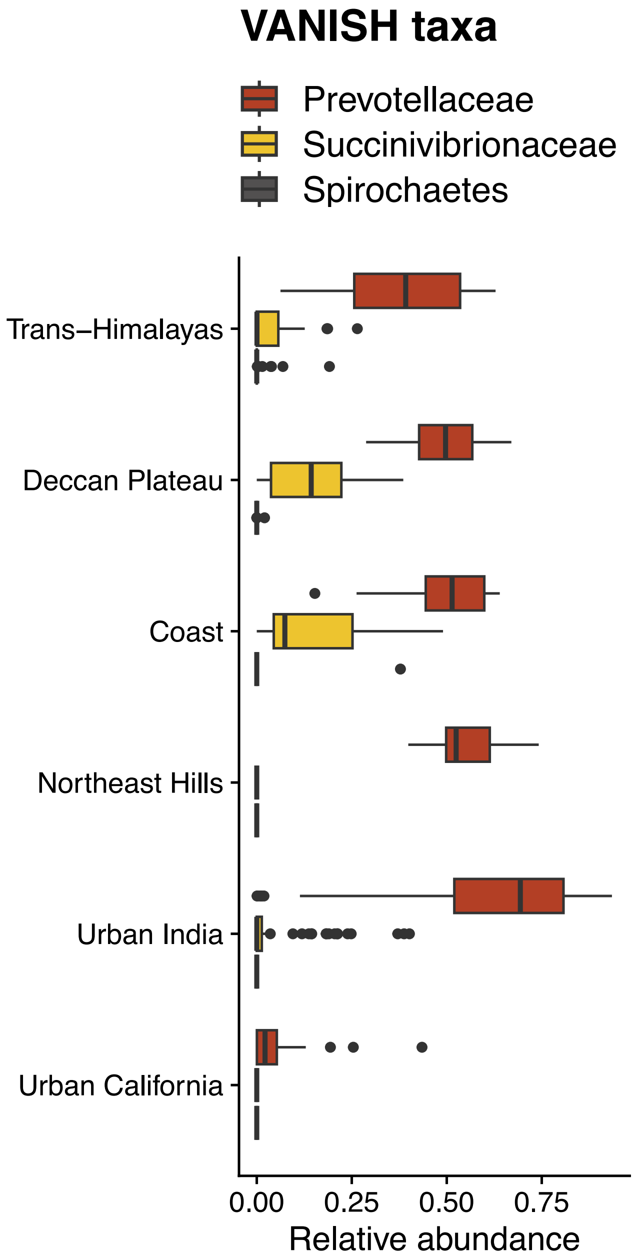

Figure 4 Supplement 1

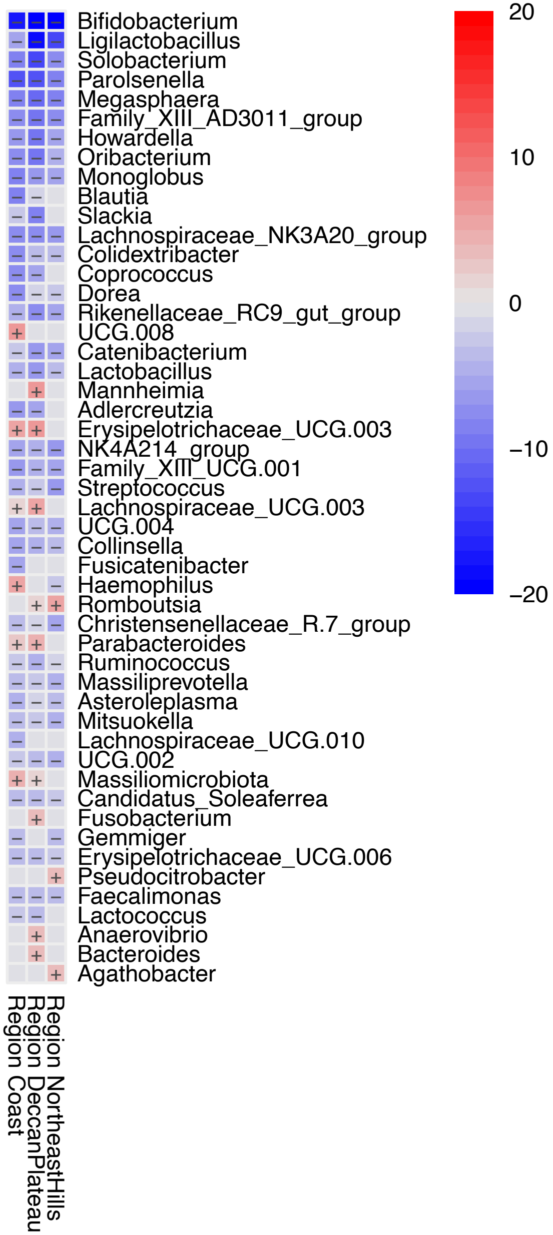

Figure 4 Supplement 2

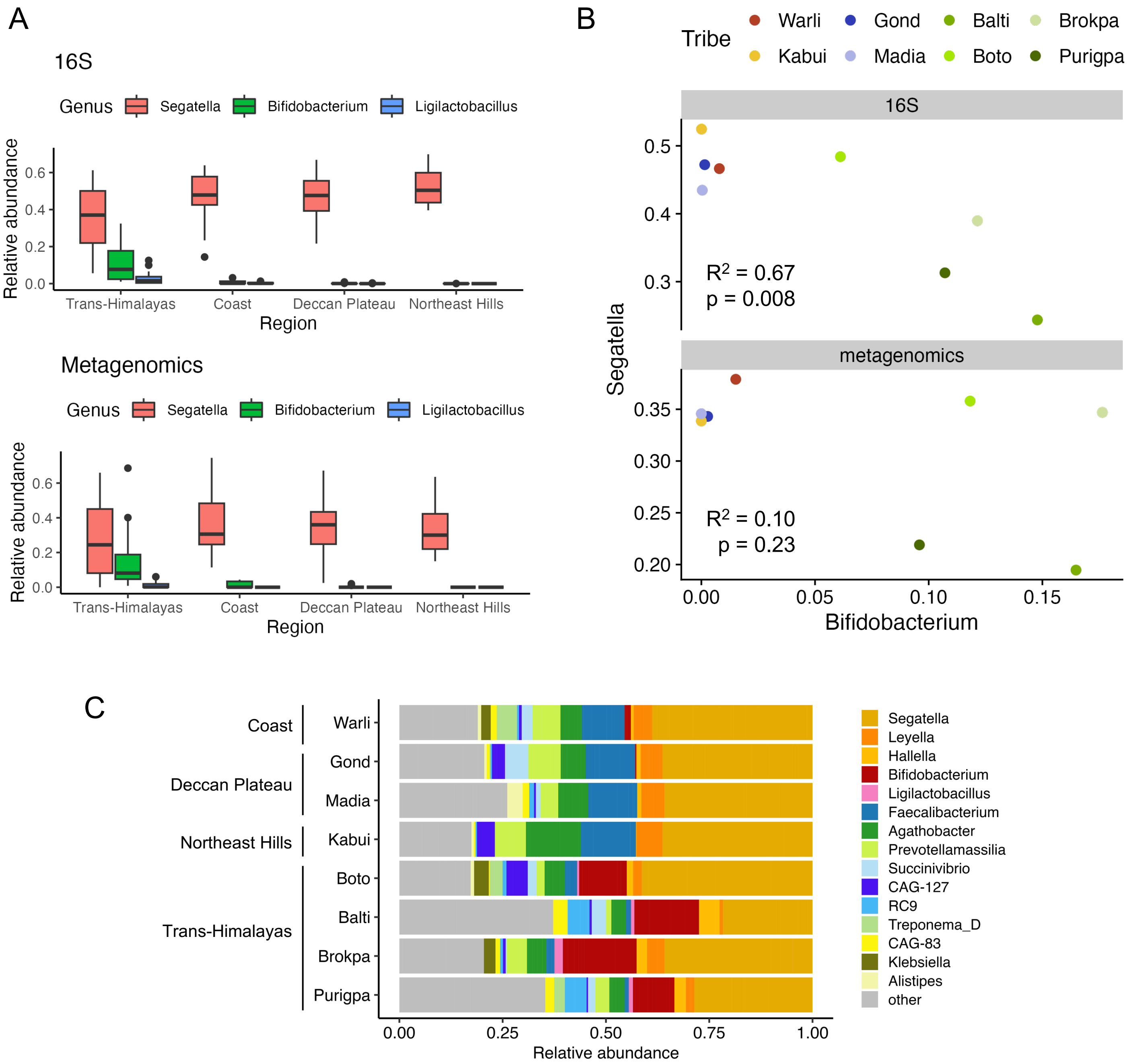

Figure 5 Supplement 1

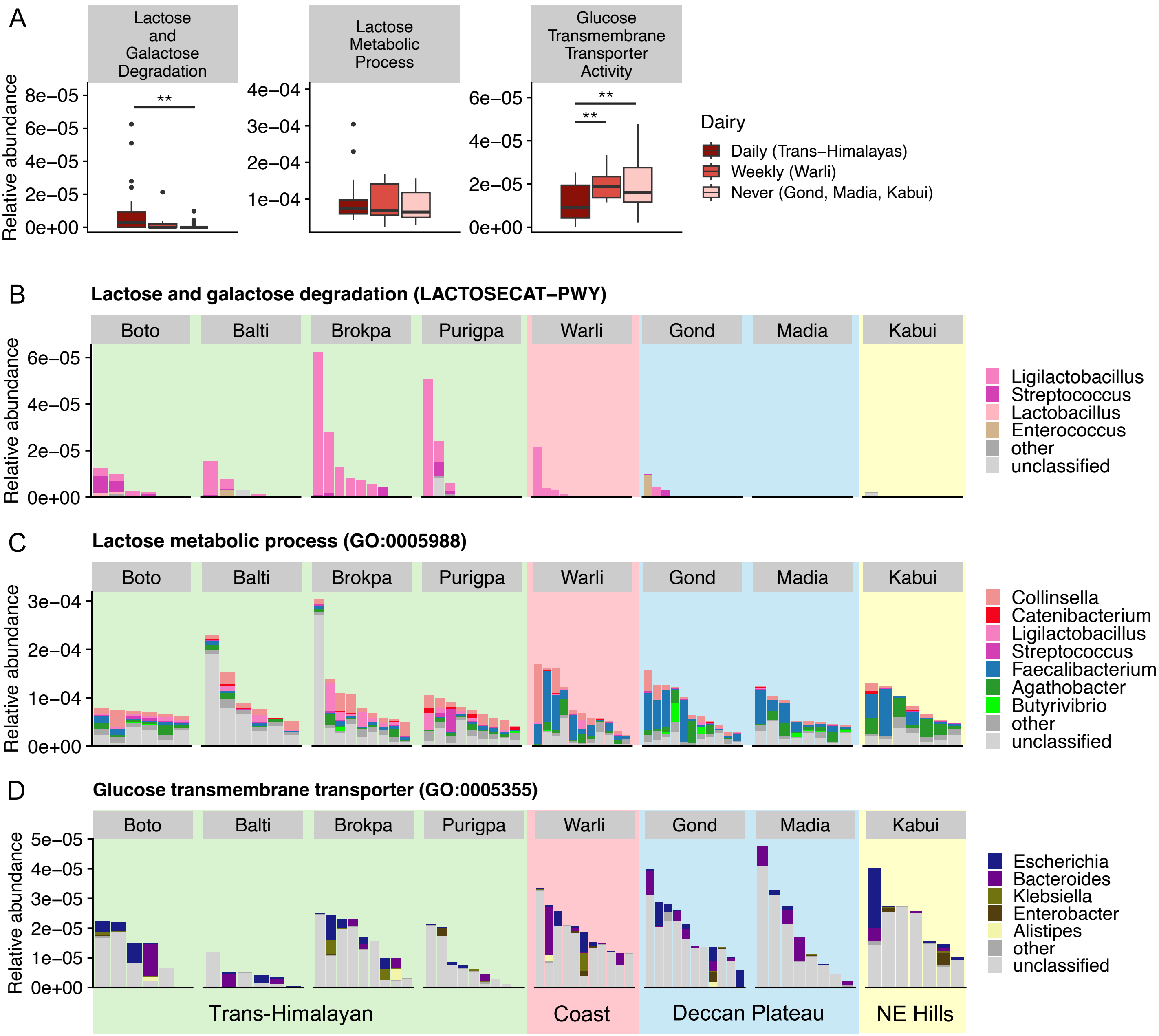

Figure 6 Supplement 1

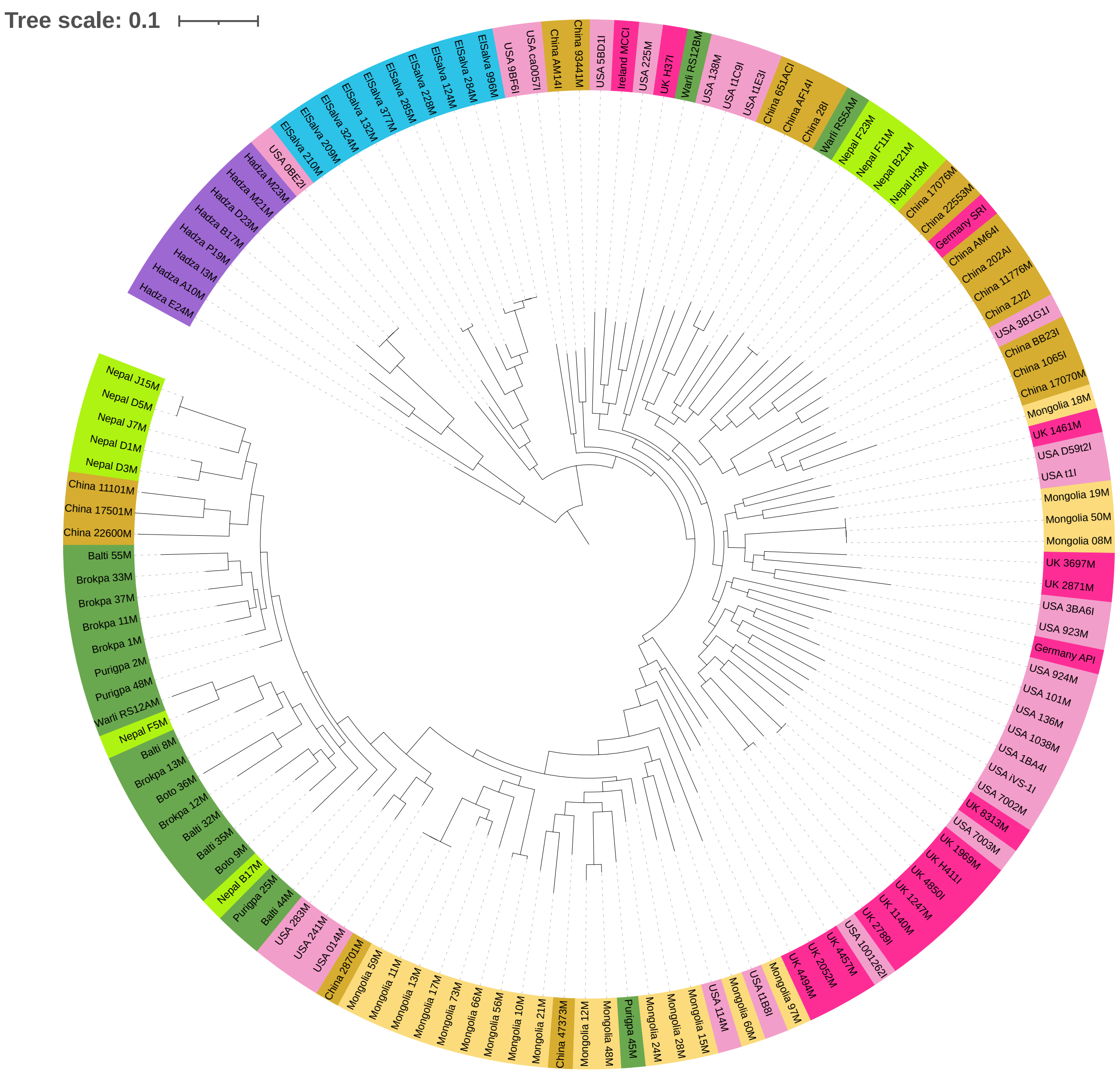

Figure 6 Supplement 2

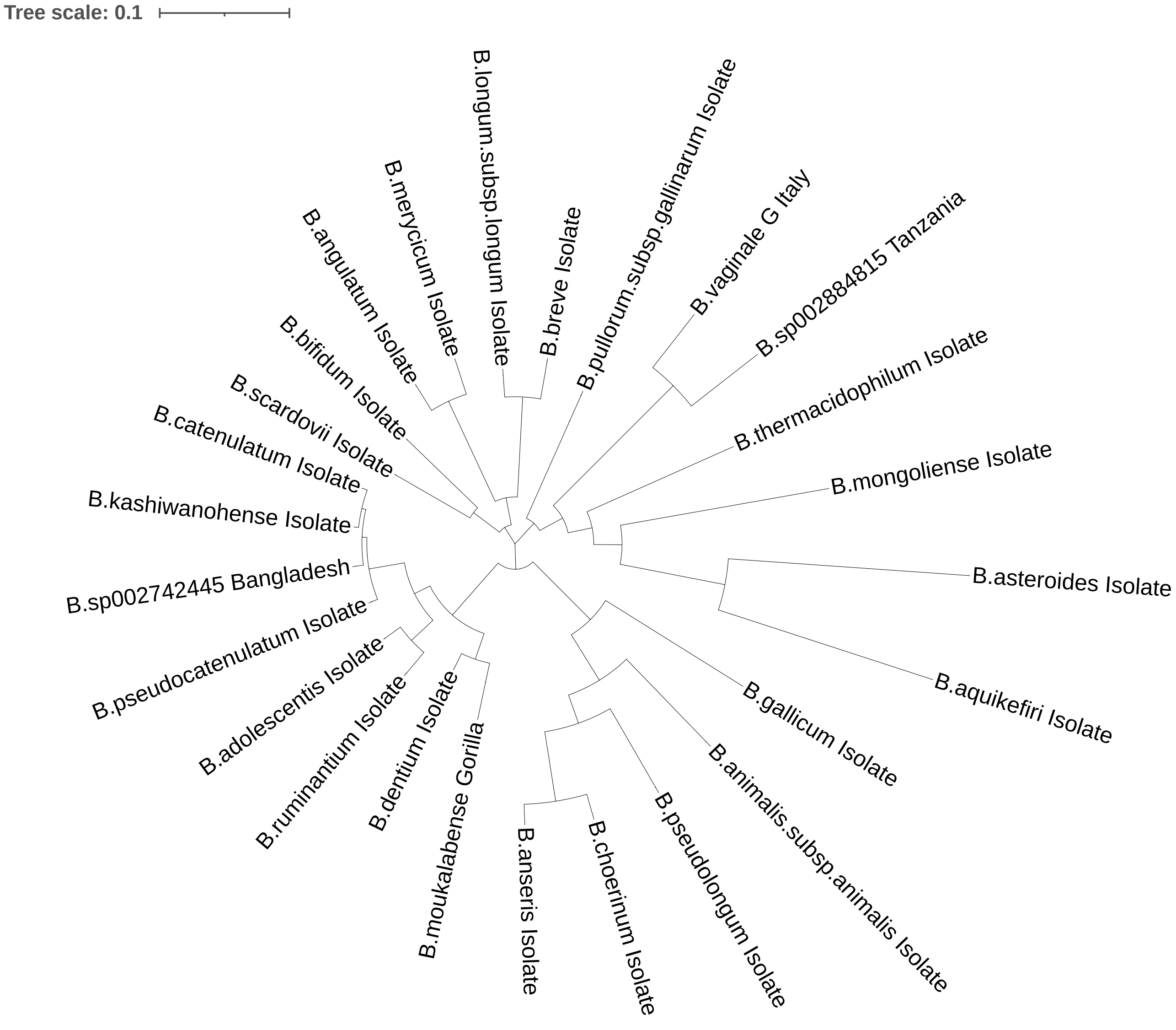

Supplement: REVISED Supplemental Figures.pdf [file KGMI_A_2694242_SM8683.pdf]
