# Supplementary material for: Gut microbiomes of tribal communities in India vary with dairy and grain consumption
Source: Gut Microbes. 2026 Jul 9;18(1):2694242. doi: 10.1080/19490976.2026.2694242 (PMC13353789; doi:10.1080/19490976.2026.2694242)
Supplement: Supplementary Materials excluding Figures.zip [file KGMI_A_2694242_SM8684.zip › Supplementary Materials excluding Figures/File S2 - Food Frequency Questionnaire.pdf]

Tribal Microbiome Study

Food Frequency Questionnaire

Code	Food-items	Freq	Code	Food-item	Freq
**A**	**BEVERAGES**		**I**	**OTHER VEGETABLES**	
1	Tea /Coffee		1	Commonly consumed	
2	Milk		2	Banana flower	
3	Others		3	Raw jackfruit	
4			4	Community specific	
5			5	Mushrooms	
6			6		
**B**	**MILK PRODUCTS**		7		
1	Butter / ghee		8		
2	Paneer / cheese		**J**	**GREEN LEAFY VEGETABLE**	
3	Curd / Buttermilk		1	Commonly consumed	
4	Sweets		2	Community specific	
5	Others		3	Others	
6			4		
7			5		
8			6		
**C**	**NON VEGETERIAN**		**K**	**RAW UNCOOKED FOODS**	
1	Meat / Raw meat		1	Cucumber/tomato/capsicum	
2	Fish		2	Radish/ carrot /beet	
3	Eggs		3	Mint / coriander / other leaves	
4	Dry fish/Dry meat/Smoked meat		4	Others	
5	Others		5		
6			6		
7			7		
8			8		
**D**	**STAPLE CEREAL**		**L**	**FERMENTED FOODS**	
1	Jowar roti		1	Idli / dosa / Dhokla	
2	Wheat roti		2	Beer<br>(rice/other fermented foods)	
3	Rice		3		
4	Bajara / Ragi		4		
5	Others		5		
6			6		
7			7		
8			8		
9			9		
**L**	**FRUITS**		**H**	**BAKERY PRODUCTS**	
1	Banana / Sapota		1	Bread / toast / butter	
2	Citrsu :<br>Orange/ Sweet Lime / Amla		2	Biscuits / khari / pattice	
3	Guava /Apple /Papaya		3	Cakes /pastries /cream role	
4	Maize		4	Others	
5	Others		5		
6			6		
7			7		

Tribal Microbiome Study

Food Frequency Questionnaire

Code	Food-items	Freq	Code	Food-item	Freq
8			**J**	**OUTSIDE/STREET FOOD**	
**E**	**PULSE/DAL**		1	Snacks	
1	Red Gram / Green Gram		2	Deep fried foods	
2	Black Gram / Bengal Gram		3	Outside meals	
3	Others		4		
4			5		
5			6		
**F**	**Whole Pulses**		**K**	**PROCESSED FOODS**	
1	Moth bean / Moong		1	Ready to eat foods	
2	Cowpea / Lentil /		2	Soups/juices	
3	Val / Rajma		3	Others	
4	Others		4		
5			5		
6			**L**	**SPICES**	
7			1	Garlic/ginger /curcumin	
**G**	**SPROUTS**		2	Medicinal herbs/leaves	
1	Sprouts of pulses		3		
2	Bamboo shoots		4		
3	Region specific special food		5		
4	Other		6		
5			**M**	**Type of Oil used in cooking (specify)**	
6			N	**FAST FOOD**	
**H**	**ROOT/TUBER/VEGETABLE**		1	Pizza/Burger	
1	Potato /Sweet potato		2	Oily/ fried/ Chips/ Others	
2	Radish / carrot /beet / Colocasia		3	Namkeen	
3	Tapioca / Region specific		4		
4			5		

D1 - Once / day W1 - Once /week M1 - Once / month
D2 - Twice or more / day W2 - Twice /week M2 - Twice / month N - Never

Name of the interviewer: ------------------------ Checked by: --------------------------------------

Checked by: ______________________ Signature: _____________ Date: □□/□□/□□□□	
Data entry by: ______________________ Signature: _____________ Date: □□/□□/□□□□	
