# Supplementary material for: Gut microbiomes of tribal communities in India vary with dairy and grain consumption
Source: Gut Microbes. 2026 Jul 9;18(1):2694242. doi: 10.1080/19490976.2026.2694242 (PMC13353789; doi:10.1080/19490976.2026.2694242)
Supplement: Supplementary Materials excluding Figures.zip [file KGMI_A_2694242_SM8684.zip › Supplementary Materials excluding Figures/File S4 - Health Status.pdf]

Tribal Microbiome Study

Health Status Questionnaire (Adult)

Q001	Did you suffer from any illness within the past 6 months?<br><br>(Probe for goitre/ thyroid disease,cancer and gastrointestinal disorders like: chronic constipation, dysentery, diarrhoea, lactose intolerance, celiac disease etc.)	1. Yes	2. No
Q002	Did you underwent a surgery within the past 6 months?	1. Yes	2. No
Q003	If yes, please mention		
Q004	Do you have a family history of a particular illness?<br>(eg: Diabetes, B.P, cardiovascular disease etc.)	1. Yes	2. No
Q005	Did you take any medication and antibiotics for the illness mentioned above?	1. Yes	2. No
Q006	If yes, please mention		
Q007	Do you suffer from HIV / AIDS?	1. Yes	2. No
Q008	Do you have cough/ fever for more than 21 days?	1. Yes	2. No
Q009	Did you take a de-worming tablet within the past 3 months?	1. Yes	2. No
Q010	Have you ever had unusual constipation or frequent loose stool since one month?	1. Yes	2. No
Q011	Have you had acute /chronic/frequent abdominal pain?	1. Yes	2. No
Q012	Do you frequently feel a burning sensation in the stomach?	1. Yes	2. No
Q013	Do you have pimples/ watery lesions/warts/wounds/white patches/rashes on your face, chest or arms?	1. Yes	2. No
Q014	Do you frequently have excessive skin itching?	1. Yes	2. No
Q015	Do you have frequent toothache or dental caries?	1. Yes	2. No
Q016	Do you have any ulcer/lesion in the oral cavity?	1. Yes	2. No
Q017	Have you experienced unusually frequent/ very low/ burning sensation<br>/ pain in urination recently/ in past one month?	1. Yes	2. No
Q018	Do you take any health supplements? (By tablet / injections)<br><br>(eg : iron / vitamin)	1. Yes	2. No
Q019	If yes, Please mention<br>______________________________		
Q020	Do you suffer from any illness from birth?	1. Yes	2. No

Health Status Questionnaire (Adult)

Q021	If yes, please mention ______________________________	
Q022	Do you suffer from asthma?	1. Yes 2. No
Q023	Do you suffer from anaemia?	1. Yes 2. No
Q024	Can you recall your blood haemoglobin count from last test	______________________
Q025	Do you take any supplementary food / powder?	1. Yes 2. No
Q026	If yes, plz mention ______________________________	
Q027	Do you suffer from arthritis?	1. Yes 2. No
	**For the female Study participants**	
Q028	What was your last menstruation date?	
Q029	Do you have unusually heavy/ scanty menstruation bleeding?	01.Yes 02. No
Q030	Do you have irregular or painful menstruation?	01.Yes 02. No
Q031	Do you have any abnormal vaginal discharge?	01.Yes 02. No
Q032	Whether you have sudden gain or loss of weight	01.Yes 02. No
