# Supplementary material for: Gut microbiomes of tribal communities in India vary with dairy and grain consumption
Source: Gut Microbes. 2026 Jul 9;18(1):2694242. doi: 10.1080/19490976.2026.2694242 (PMC13353789; doi:10.1080/19490976.2026.2694242)
Supplement: Supplementary Materials excluding Figures.zip [file KGMI_A_2694242_SM8684.zip › Supplementary Materials excluding Figures/File S3 - Inclusion Exclusion Criteria.pdf]

Tribal Microbiome Study

Inclusion-Exclusion Sheet

You are being invited to participate in a research study conducted by National Centre for Cell Science, Pune and Institute of Bioresources and Sustainable Development, Imphal. Taking part in this study is voluntary. To determine your eligibility for our proposed study, we would to ask you few questions with your verbal consent.

Name of Study participant: ____

**Inclusion Criteria**	**Yes/ No**
• Is your age between 18 – 55 years?	
• Self-declared healthy individual<br>(Probe for chronic disorders like Blood pressure, Diabetes, Cardiovascular diseases etc.)	
• Generations Endogamy questions<br>Which community do you belong to? ___________________________________<br>1. Do your parents belong to the same caste /endogamous group as each other?<br>2. Do both your paternal grandparents belong to the same caste /endogamous group as each other?<br>3. Do both your maternal grandparents belong to the same caste /endogamous group as each other?<br>All three should be answered yes. Then included. Same question could be asked for spouse, does your spouse belong to the same endogamous group as you? Then he/she may be recruited using separate questionnaire.	
**Exclusion Criteria**	
• Did you suffer from any illness/injury in the past 3 months?<br>If yes, plz specify __________________<br>(Probe : Any illness involving antibiotic treatment within the past 3 months)	
• Have you taken any antibiotics in the past 3 months?<br>If yes, plz specify __________________<br>(Any mild tonic/ homeopathic/ herbal/ ayurvedic / traditional medicines is allowed.)	
• Have you suffered from any stomach related issues in the past 3 months?<br>(Probe for constipation, dysentery, stomach pain, ulcer etc.)	
• For Women only :<br>1. Are you currently pregnant?<br>2. Are you breast-feeding your child?	
