# Supplementary material for: Gut microbiomes of tribal communities in India vary with dairy and grain consumption
Source: Gut Microbes. 2026 Jul 9;18(1):2694242. doi: 10.1080/19490976.2026.2694242 (PMC13353789; doi:10.1080/19490976.2026.2694242)
Supplement: Supplementary Materials excluding Figures.zip [file KGMI_A_2694242_SM8684.zip › Supplementary Materials excluding Figures/Table S3. Dairy references.pdf]

| Taxon | Evidence Type | References |
|---|---|---|
| *Bifidobacterium* | Fermented dairy | (Delcenserie et al., 2007; Watanabe et al., 2009) |
| *Bifidobacterium* | Grows on lactose | (González-Morelo et al., 2023) |
| *Bifidobacterium* | Raw milk | (Carafa et al., 2020; Delcenserie et al., 2007; Gryaznova et al., 2021) |
| *Bifidobacterium* | Cattle rumen | (Vlková et al., 2006; Y. Wang et al., 2021) |
| *Bifidobacterium* | Cattle feces | (Albert & Sela, 2018) |
| *Bifidobacterium* | Cattle reproductive | (Brulin et al., 2024; Frétin et al., 2018; Moraes et al., 2024; Shakhov, AG et al., 2018; Winders et al., 2023; Yang, Bajinka, et al., 2021) |
| *Ligilactobacillus* | Fermented dairy | (Linares et al., 2017) |
| *Ligilactobacillus* | Grows on lactose | (S. S. Zhang et al., 2020) |
| *Ligilactobacillus* | Raw milk | (Carafa et al., 2020; Frétin et al., 2018) |
| *Ligilactobacillus* | Cattle rumen | (Astriani, M. et al., 2020) |
| *Ligilactobacillus* | Cattle feces | (Coelho et al., 2022) |
| *Ligilactobacillus* | Cattle reproductive | (Verdier-Metz et al., 2012) |
| *Megasphaera* | Fermented dairy | (Fuka et al., 2013; Salazar et al., 2018) |
| *Megasphaera* | Grows on lactose | (Marounek et al., 1989) |
| *Megasphaera* | Cattle rumen | (Marounek et al., 1989; Ouwerkerk et al., 2002) |
| *Megasphaera* | Cattle feces | (Uchiyama et al., 2020; Ziemer, 2014) |
| *Collinsella* | Fermented dairy | (Rettedal et al., 2019) |
| *Collinsella* | Grows on lactose | (Qin et al., 2019) |
| *Collinsella* | Raw milk | (Guo et al., 2024; Rettedal et al., 2019) |
| *Collinsella* | Cattle rumen | (Pitta et al., 2016) |
| *Collinsella* | Cattle feces | (Kojima et al., 2022; Liang et al., 2022) |
| *Collinsella* | Cattle reproductive | (Messman et al., 2021; Wege Dias, 2023) |
| Rikenellaceae_RC9_gut_group | Raw milk | (Zhu et al., 2023) |

| | | |
|---|---|---|
| Rikenellaceae_RC9_gut_group | Cattle rumen | (Sha et al., 2021; K. Zhang et al., 2024) |
| Rikenellaceae_RC9_gut_group | Cattle feces | (Jia et al., 2023; Liang et al., 2022; Pang et al., 2022) |
| Rikenellaceae_RC9_gut_group | Cattle reproductive | (Moraes et al., 2024) |
| NK4A214_group | Raw milk | (Zhu et al., 2023) |
| NK4A214_group | Cattle rumen | (Dai et al., 2021; Guo et al., 2023; Pan et al., 2017; Wu et al., 2023) |
| NK4A214_group | Cattle feces | (Witzke, 2020) |
| NK4A214_group | Cattle reproductive | (Amat et al., 2021, p. 202) |
| *Parolsenella* | Grows on lactose | (Bilen et al., 2017) |
| *Parolsenella* | Cattle reproductive | (Wege Dias, 2023) |
| *Monoglobus* | Raw milk | (Mtshali et al., 2022; Zhu et al., 2023) |
| *Monoglobus* | Cattle rumen | (Gao et al., 2022; Zang et al., 2022, p. 202) |
| *Monoglobus* | Cattle feces | (Koester et al., 2020; Mtshali et al., 2022; L. Wang et al., 2023) |
| *Monoglobus* | Cattle reproductive | (Brulin et al., 2024) |
| *Oribacterium* | Grows on lactose | (Sizova et al., 2014) |
| *Oribacterium* | Cattle rumen | (Kang et al., 2019; See, 2022) |
| *Oribacterium* | Cattle feces | (De La Guardia Hidrogo, 2021) |
| *Oribacterium* | Cattle reproductive | (Winders et al., 2023) |
| Lachnospiraceae NK3A20 group | Raw milk | (Van De Grift, 2021) |
| Lachnospiraceae NK3A20 group | Cattle rumen | (Lopez Franco, 2019; See, 2022) |
| Lachnospiraceae NK3A20 group | Cattle feces | (See, 2022) |
| Lachnospiraceae NK3A20 group | Cattle reproductive | (Yagisawa et al., 2023) |
| UCG.004 | Cattle rumen | (Lee et al., 2024) |
| UCG.004 | Cattle feces | (Fan et al., 2021) |
| *Solobacterium* | Cattle rumen | (Barrett et al., 2022; Fregulia et al., 2022) |
| *Solobacterium* | Cattle feces | (See, 2022) |
| *Solobacterium* | Cattle reproductive | (Verdier-Metz et al., 2012) |

| | | |
|---|---|---|
| Family_XIII_AD3011_group | Raw milk | (Coates et al., 2022; Zhu et al., 2023) |
| Family_XIII_AD3011_group | Cattle rumen | (Zou et al., 2022) |
| Family_XIII_AD3011_group | Cattle feces | (Mahayri et al., 2022) |
| Family_XIII_AD3011_group | Cattle reproductive | (Quadros et al., 2020) |
| *Colidextribacter* | Raw milk | (Gryaznova et al., 2021; She et al., 2023) |
| *Colidextribacter* | Cattle rumen | (Hao et al., 2024; Shang et al., 2024) |
| *Colidextribacter* | Cattle feces | (Kim et al., 2024) |
| *Colidextribacter* | Cattle reproductive | (Brulin et al., 2024) |
| *Lactobacillus* | Fermented dairy | (Linares et al., 2017) |
| *Lactobacillus* | Grows on lactose | (S. S. Zhang et al., 2020) |
| *Lactobacillus* | Raw milk | (Carafa et al., 2020; Frétin et al., 2018; Zhu et al., 2023) |
| *Lactobacillus* | Cattle rumen | (Astriani, M. et al., 2020) |
| *Lactobacillus* | Cattle feces | (Coelho et al., 2022) |
| *Lactobacillus* | Cattle reproductive | (Moraes et al., 2024; Shakhov, AG et al., 2018; Verdier-Metz et al., 2012; Webb et al., 2023, 2023; Yang, Huang, et al., 2021) |
| *Howardella* | Cattle rumen | (Fregulia et al., 2022; Monteiro et al., 2022) |
| *Howardella* | Cattle feces | (See, 2022) |
| *Howardella* | Cattle reproductive | (Webb et al., 2023) |
| Family_XIII_UCG.001 | Cattle rumen | (McLoughlin et al., 2023) |
| Family_XIII_UCG.001 | Cattle feces | (Bibbal et al., 2022; De La Guardia Hidrogo, 2021) |
| Family_XIII_UCG.001 | Cattle reproductive | (Quadros et al., 2020) |
| *Faecalimonas* | Grows on lactose | (Sakamoto et al., 2017) |
| *Faecalimonas* | Cattle rumen | (Ogata et al., 2020) |

Albert, K., & Sela, D. A. (2018). Draft Genome Sequence of Bifidobacterium longum UMA026, Isolated from Holstein Dairy Cow Feces. *Genome Announcements*, *6*(25), 10.1128/genomea.00559-18. https://doi.org/10.1128/genomea.00559-18

Amat, S., Holman, D. B., Schmidt, K., Menezes, A. C. B., Baumgaertner, F., Winders, T., Kirsch, J. D., Liu, T., Schwinghamer, T. D., Sedivec, K. K., & Dahlen, C. R. (2021). The Nasopharyngeal, Ruminal, and Vaginal Microbiota and the Core Taxa Shared across These Microbiomes in Virgin Yearling Heifers Exposed to Divergent In Utero Nutrition during Their First Trimester of Gestation and in Pregnant Beef Heifers in Response to Mineral Supplementation. *Microorganisms*, *9*(10), 2011. https://doi.org/10.3390/microorganisms9102011

Astriani, M., Zubaidah, S., Abadi, A. L., & Suarsini, E. (2020). Isolation and identification of phosphate solubilizing bacteria from indigenous microorganisms (IMO) of cow rumen in East Java, Indonesia as eco-friendly biofertilizer. *Malaysian Journal of Microbiology*. https://doi.org/10.21161/mjm.190536

Barrett, K., Lange, L., Børsting, C. F., Olijhoek, D. W., Lund, P., & Meyer, A. S. (2022). Changes in the Metagenome-Encoded CAZymes of the Rumen Microbiome Are Linked to Feed-Induced Reductions in Methane Emission From Holstein Cows. *Frontiers in Microbiology*, *13*. https://www.frontiersin.org/articles/10.3389/fmicb.2022.855590

Bibbal, D., Ruiz, P., Sapountzis, P., Mazuy-Cruchaudet, C., Loukiadis, E., Auvray, F., Forano, E., & Brugère, H. (2022). Persistent Circulation of Enterohemorrhagic Escherichia coli (EHEC) O157:H7 in Cattle Farms: Characterization of Enterohemorrhagic Escherichia coli O157:H7 Strains and Fecal Microbial Communities of Bovine Shedders and Non-shedders. *Frontiers in Veterinary Science*, *9*. https://www.frontiersin.org/articles/10.3389/fvets.2022.852475

Bilen, M., Cadoret, F., Richez, M., Tomei, E., Daoud, Z., Raoult, D., & Fournier, P.-E. (2017). Libanicoccus massiliensis gen. Nov., sp. Nov., a new bacterium isolated from human stool. *New Microbes and New Infections*, *21*, 63–71. https://doi.org/10.1016/j.nmni.2017.11.001

Brulin, L., Ducrocq, S., Even, G., Sanchez, M. P., Martel, S., Merlin, S., Audebert, C., Croiseau, P., & Estellé, J. (2024). Characterization of bovine vaginal microbiota using 16S rRNA sequencing: Associations with host fertility, longevity, health, and production. *Scientific Reports*, *14*(1), 19277. https://doi.org/10.1038/s41598-024-69715-7

Carafa, I., Navarro, I. C., Bittante, G., Tagliapietra, F., Gallo, L., Tuohy, K., & Franciosi, E. (2020). Shift in the cow milk microbiota during alpine pasture as analyzed by culture dependent and high-throughput sequencing techniques. *Food Microbiology*, *91*, 103504. https://doi.org/10.1016/j.fm.2020.103504

Coates, L. C., Storms, D., Finley, J. W., Fukagawa, N. K., Lemay, D. G., Kalscheur, K. F., & Kable, M. E. (2022). A Low-Starch and High-Fiber Diet Intervention Impacts the Microbial Community of Raw Bovine Milk. *Current Developments in Nutrition*, *6*(6), nzac086. https://doi.org/10.1093/cdn/nzac086

Coelho, M. G., Virgínio Júnior, G. F., Tomaluski, C. R., de Toledo, A. F., Reis, M. E., Dondé, S. C., Mendes, L. W., Coutinho, L. L., & Bittar, C. M. M. (2022). Comparative study of different liquid diets for dairy calves and the impact on performance and the bacterial community during diarrhea. *Scientific Reports*, *12*(1), Article 1. https://doi.org/10.1038/s41598-022-17613-1

Dai, Q., Ma, J., Cao, G., Hu, R., Zhu, Y., Li, G., Zou, H., Wang, Z., Peng, Q., Xue, B., & Wang, L. (2021). Comparative study of growth performance, nutrient digestibility, and ruminal and fecal bacterial community between yaks and cattle-yaks raised by stall-feeding. *AMB Express*, *11*(1), 98. https://doi.org/10.1186/s13568-021-01259-9

De La Guardia Hidrogo, V. M. (2021). *Associations Between the Gastrointestinal Microbiome and Nitrogen Efficiency in Holstein and Jersey Cows* [M.S., Mississippi State University].

https://www.proquest.com/docview/2572607843/abstract/371579098FF84AABPQ/1
Delcenserie, V., Gavini, F., Beerens, H., Tresse, O., Franssen, C., & Daube, G. (2007). Description of a new species, *Bifidobacterium crudilactis* sp. Nov., isolated from raw milk and raw milk cheeses. *Systematic and Applied Microbiology*, *30*(5), 381–389. https://doi.org/10.1016/j.syapm.2007.01.004
Fan, P., Kim, M., Liu, G., Zhai, Y., Liu, T., Driver, J. D., & Jeong, K. C. (2021). The Gut Microbiota of Newborn Calves and Influence of Potential Probiotics on Reducing Diarrheic Disease by Inhibition of Pathogen Colonization. *Frontiers in Microbiology*, *12*. https://doi.org/10.3389/fmicb.2021.772863
Fregulia, P., Campos, M. M., Dias, R. J. P., Liu, J., Guo, W., Pereira, L. G. R., Machado, M. A., Faza, D. R. de L. R., Guan, L. L., Garnsworthy, P. C., & Neves, A. L. A. (2022). Taxonomic and predicted functional signatures reveal linkages between the rumen microbiota and feed efficiency in dairy cattle raised in tropical areas. *Frontiers in Microbiology*, *13*. https://www.frontiersin.org/articles/10.3389/fmicb.2022.1025173
Frétin, M., Martin, B., Rifa, E., Isabelle, V.-M., Pomiès, D., Ferlay, A., Montel, M.-C., & Delbès, C. (2018). Bacterial community assembly from cow teat skin to ripened cheeses is influenced by grazing systems. *Scientific Reports*, *8*(1), Article 1. https://doi.org/10.1038/s41598-017-18447-y
Fuka, M. M., Wallisch, S., Engel, M., Welzl, G., Havranek, J., & Schloter, M. (2013). Dynamics of Bacterial Communities during the Ripening Process of Different Croatian Cheese Types Derived from Raw Ewe's Milk Cheeses. *PLOS ONE*, *8*(11), e80734. https://doi.org/10.1371/journal.pone.0080734
Gao, J., Cheng, B. B., Liu, Y. F., Li, M. M., & Zhao, G. Y. (2022). Effects of red cabbage extract rich in anthocyanins on rumen fermentation, rumen bacterial community, nutrient digestion, and plasma indices in beef bulls. *Animal*, *16*(5), 100510. https://doi.org/10.1016/j.animal.2022.100510
González-Morelo, K. J., Galán-Vásquez, E., Melis, F., Pérez-Rueda, E., & Garrido, D. (2023). Structure of co-expression networks of Bifidobacterium species in response to human milk oligosaccharides. *Frontiers in Molecular Biosciences*, *10*. https://www.frontiersin.org/articles/10.3389/fmolb.2023.1040721
Gryaznova, M. V., Syromyatnikov, M. Y., Dvoretskaya, Y. D., Solodskikh, S. A., Klimov, N. T., Mikhalev, V. I., Zimnikov, V. I., Mikhaylov, E. V., & Popov, V. N. (2021). Microbiota of Cow's Milk with Udder Pathologies. *Microorganisms*, *9*(9), Article 9. https://doi.org/10.3390/microorganisms9091974
Guo, W., Bi, S. S., Wang, W. W., Zhou, M., Neves, A. L. A., Degen, A. A., Guan, L. L., & Long, R. J. (2023). Maternal rumen and milk microbiota shape the establishment of early-life rumen microbiota in grazing yak calves. *Journal of Dairy Science*, *106*(3), 2054–2070. https://doi.org/10.3168/jds.2022-22655
Guo, W., Liu, S., Khan, M. Z., Wang, J., Chen, T., Alugongo, G. M., Li, S., & Cao, Z. (2024). Bovine milk microbiota: Key players, origins, and potential contributions to early-life gut development. *Journal of Advanced Research*, *59*, 49–64. https://doi.org/10.1016/j.jare.2023.06.016
Hao, Y., Xia, J., Wang, W., Wang, Y., Cao, Z., Yang, H., Jiang, L., Ma, Z., Chu, K., Wang, S., Guan, L. L., & Li, S. (2024). Diurnal shifts of rumen fermentation and microbial profiles revealed circadian rhythms of rumen bacteria, methanogens, and protozoa under high-grain and high-forage diets. *JDS Communications*, *5*(6), 700–706. https://doi.org/10.3168/jdsc.2023-0526
Jia, X., He, Y., Kang, Z., Chen, S., Sun, W., Wang, J., & Lai, S. (2023). Comparison of Fecal Microbiota Communities between Primiparous and Multiparous Cows during Non-Pregnancy and Pregnancy. *Animals*, *13*(5), Article 5. https://doi.org/10.3390/ani13050869

Kang, S., Denman, S., & McSweeney, C. (2019). Draft Genome Sequence and Annotation of Oribacterium sp. Strain C9, Isolated from a Cattle Rumen. *Microbiology Resource Announcements*, *8*(13), 10.1128/mra.01562-18. https://doi.org/10.1128/mra.01562-18

Kim, H., Jo, J.-H., Lee, H.-G., Park, W., Lee, H.-K., Park, J.-E., & Shin, D. (2024). Inflammatory response in dairy cows caused by heat stress and biological mechanisms for maintaining homeostasis. *PLOS ONE*, *19*(3), e0300719. https://doi.org/10.1371/journal.pone.0300719

Koester, L. R., Poole, D. H., Serão, N. V. L., & Schmitz-Esser, S. (2020). Beef cattle that respond differently to fescue toxicosis have distinct gastrointestinal tract microbiota. *PLOS ONE*, *15*(7), e0229192. https://doi.org/10.1371/journal.pone.0229192

Kojima, M., Liu, H., Takemoto, S., Suda, Y., Inoue, R., Watanabe, G., Jin, W., & Nagaoka, K. (2022). Temporal changes in the fecal microbiome and blood metabolites of early neonatal calves. *Frontiers in Animal Science*, *3*. https://doi.org/10.3389/fanim.2022.934204

Lee, H., Kim, M., Masaki, T., Ikuta, K., Iwamoto, E., Nishihara, K., Nonaka, I., Ashihara, A., Baek, Y., Lee, S., Uemoto, Y., Haga, S., Terada, F., & Roh, S. (2024). Assessing the impact of three feeding stages on rumen bacterial community and physiological characteristics of Japanese Black cattle. *Scientific Reports*, *14*(1), 4923. https://doi.org/10.1038/s41598-024-55539-y

Liang, Z., Zhang, J., Du, M., Ahmad, A. A., Wang, S., Zheng, J., Salekdeh, G. H., Yan, P., Han, J., Tong, B., & Ding, X. (2022). Age-dependent changes of hindgut microbiota succession and metabolic function of Mongolian cattle in the semi-arid rangelands. *Frontiers in Microbiology*, *13*. https://doi.org/10.3389/fmicb.2022.957341

Linares, D. M., Gómez, C., Renes, E., Fresno, J. M., Tornadijo, M. E., Ross, R. P., & Stanton, C. (2017). Lactic Acid Bacteria and Bifidobacteria with Potential to Design Natural Biofunctional Health-Promoting Dairy Foods. *Frontiers in Microbiology*, *8*. https://www.frontiersin.org/articles/10.3389/fmicb.2017.00846

Lopez Franco, J. (2019). *Correlations between concentration of vitamin B12 in milk and the composition of the bovine microbiota*. McGill University. https://escholarship.mcgill.ca/concern/theses/j9602520n

Mahayri, T. M., Fliegerová, K. O., Mattiello, S., Celozzi, S., Mrázek, J., Mekadim, C., Sechovcová, H., Kvasnová, S., Atallah, E., & Moniello, G. (2022). Host Species Affects Bacterial Evenness, but Not Diversity: Comparison of Fecal Bacteria of Cows and Goats Offered the Same Diet. *Animals*, *12*(16), Article 16. https://doi.org/10.3390/ani12162011

Marounek, M., Fliegrova, K., & Bartos, S. (1989). Metabolism and some characteristics of ruminal strains of Megasphaera elsdenii. *Applied and Environmental Microbiology*, *55*(6), 1570–1573. https://doi.org/10.1128/aem.55.6.1570-1573.1989

McLoughlin, S., Spillane, C., Campion, F. P., Claffey, N., Sosa, C. C., McNicholas, Y., Smith, P. E., Diskin, M. G., & Waters, S. M. (2023). Breed and ruminal fraction effects on bacterial and archaeal community composition in sheep. *Scientific Reports*, *13*(1), Article 1. https://doi.org/10.1038/s41598-023-28909-1

Messman, R. D., Contreras-Correa, Z. E., Paz, H. A., & Lemley, C. O. (2021). Melatonin-induced changes in the bovine vaginal microbiota during maternal nutrient restriction. *Journal of Animal Science*, *99*(5), skab098. https://doi.org/10.1093/jas/skab098

Monteiro, H. F., Zhou, Z., Gomes, M. S., Peixoto, P. M. G., Bonsaglia, E. C. R., Canisso, I. F., Weimer, B. C., & Lima, F. S. (2022). Rumen and lower gut microbiomes relationship with feed efficiency and production traits throughout the lactation of Holstein dairy cows. *Scientific Reports*, *12*(1), Article 1. https://doi.org/10.1038/s41598-022-08761-5

Moraes, J. G. N., Gull, T., Ericsson, A. C., Poock, S. E., Caldeira, M. O., & Lucy, M. C. (2024). Establishment of the uterine microbiome following artificial insemination in virgin heifers.

*Frontiers in Microbiology*, *15*. https://doi.org/10.3389/fmicb.2024.1385505
Mtshali, K., Khumalo, Z. T. H., Kwenda, S., Arshad, I., & Thekisoe, O. M. M. (2022). Exploration and comparison of bacterial communities present in bovine faeces, milk and blood using 16S rRNA metagenomic sequencing. *PLOS ONE*, *17*(8), e0273799. https://doi.org/10.1371/journal.pone.0273799
Ogata, T., Kim, Y.-H., Iwamoto, E., Masaki, T., Ikuta, K., & Sato, S. (2020). Comparison of pH and bacterial communities in the rumen and reticulum during fattening of Japanese Black beef cattle. *Animal Science Journal*, *91*(1), e13487. https://doi.org/10.1111/asj.13487
Ouwerkerk, D., Klieve, A. V., & Forster, R. J. (2002). Enumeration of Megasphaera elsdenii in rumen contents by real-time Taq nuclease assay. *Journal of Applied Microbiology*, *92*(4), 753–758. https://doi.org/10.1046/j.1365-2672.2002.01580.x
Pan, X., Xue, F., Nan, X., Tang, Z., Wang, K., Beckers, Y., Jiang, L., & Xiong, B. (2017). Illumina Sequencing Approach to Characterize Thiamine Metabolism Related Bacteria and the Impacts of Thiamine Supplementation on Ruminal Microbiota in Dairy Cows Fed High-Grain Diets. *Frontiers in Microbiology*, *8*. https://www.frontiersin.org/articles/10.3389/fmicb.2017.01818
Pang, K., Yang, Y., Chai, S., Li, Y., Wang, X., Sun, L., Cui, Z., Wang, S., & Liu, S. (2022). Dynamics Changes of the Fecal Bacterial Community Fed Diets with Different Concentrate-to-Forage Ratios in Qinghai Yaks. *Animals*, *12*(18), Article 18. https://doi.org/10.3390/ani12182334
Pitta, D. W., Pinchak, W. E., Indugu, N., Vecchiarelli, B., Sinha, R., & Fulford, J. D. (2016). Metagenomic Analysis of the Rumen Microbiome of Steers with Wheat-Induced Frothy Bloat. *Frontiers in Microbiology*, *7*. https://doi.org/10.3389/fmicb.2016.00689
Qin, P., Zou, Y., Dai, Y., Luo, G., Zhang, X., & Xiao, L. (2019). Characterization a Novel Butyric Acid-Producing Bacterium Collinsella aerofaciens Subsp. Shenzhenensis Subsp. Nov. *Microorganisms*, *7*(3), Article 3. https://doi.org/10.3390/microorganisms7030078
Quadros, D. L., Zanella, R., Bondan, C., Zanella, G. C., Facioli, F. L., da Silva, A. N., & Zanella, E. L. (2020). Study of vaginal microbiota of Holstein cows submitted to an estrus synchronization protocol with the use of intravaginal progesterone device. *Research in Veterinary Science*, *131*, 1–6. https://doi.org/10.1016/j.rvsc.2020.03.027
Rettedal, E. A., Altermann, E., Roy, N. C., & Dalziel, J. E. (2019). The Effects of Unfermented and Fermented Cow and Sheep Milk on the Gut Microbiota. *Frontiers in Microbiology*, *10*. https://doi.org/10.3389/fmicb.2019.00458
Sakamoto, M., Iino, T., & Ohkuma, M. (2017). Faecalimonas umbilicata gen. Nov., sp. Nov., isolated from human faeces, and reclassification of Eubacterium contortum, Eubacterium fissicatena and Clostridium oroticum as Faecalicatena contorta gen. Nov., comb. Nov., Faecalicatena fissicatena comb. Nov. And Faecalicatena orotica comb. Nov. *International Journal of Systematic and Evolutionary Microbiology*, *67*(5), 1219–1227. https://doi.org/10.1099/ijsem.0.001790
Salazar, J. K., Carstens, C. K., Ramachandran, P., Shazer, A. G., Narula, S. S., Reed, E., Ottesen, A., & Schill, K. M. (2018). Metagenomics of pasteurized and unpasteurized gouda cheese using targeted 16S rDNA sequencing. *BMC Microbiology*, *18*(1), 189. https://doi.org/10.1186/s12866-018-1323-4
See, A. (2022). *Investigating Bacterial Community Composition and Antimicrobial Resistance Genes in Beef Cattle* (240) [University of Nebraska - Lincoln]. Theses and Dissertations in Animal Science. https://digitalcommons.unl.edu/animalscidiss/240
Sha, Y., Hu, J., Shi, B., Dingkao, R., Wang, J., Li, S., Zhang, W., Luo, Y., & Liu, X. (2021). Supplementary feeding of cattle-yak in the cold season alters rumen microbes, volatile fatty acids, and expression of SGLT1 in the rumen epithelium. *PeerJ*, *9*, e11048. https://doi.org/10.7717/peerj.11048

Shakhov, AG, Yerina, TA, & Sashnina, LY. (2018). Use of probiotics giprolam and simbiter-2 to correct the vagina biocenosis in down-calving cows. *AGRICULTURAL BIOLOGY*, *414*.

Shang, S., Li, J., Zhang, W., Zhang, X., Bai, J., Yang, Z., Wang, X., Fortina, R., Gasco, L., & Guo, K. (2024). Impact of High-Moisture Ear Corn on Antioxidant Capacity, Immunity, Rumen Fermentation, and Microbial Diversity in Pluriparous Dairy Cows. *Fermentation*, *10*(1), Article 1. https://doi.org/10.3390/fermentation10010044

She, Y., Liu, J., Su, M., Li, Y., Guo, Y., Liu, G., Deng, M., Qin, H., Sun, B., Guo, J., & Liu, D. (2023). A Study on Differential Biomarkers in the Milk of Holstein Cows with Different Somatic Cells Count Levels. *Animals*, *13*(15), Article 15. https://doi.org/10.3390/ani13152446

Sizova, M. V., Muller, P. A., Stancyk, D., Panikov, N. S., Mandalakis, M., Hazen, A., Hohmann, T., Doerfert, S. N., Fowle, W., Earl, A. M., Nelson, K. E., & Epstein, S. S. (2014). Oribacterium parvum sp. Nov. And Oribacterium asaccharolyticum sp. Nov., obligately anaerobic bacteria from the human oral cavity, and emended description of the genus Oribacterium. *International Journal of Systematic and Evolutionary Microbiology*, *64*(Pt 8), 2642–2649. https://doi.org/10.1099/ijs.0.060988-0

Uchiyama, J., Murakami, H., Sato, R., Mizukami, K., Suzuki, T., Shima, A., Ishihara, G., Sogawa, K., & Sakaguchi, M. (2020). Examination of the fecal microbiota in dairy cows infected with bovine leukemia virus. *Veterinary Microbiology*, *240*, 108547. https://doi.org/10.1016/j.vetmic.2019.108547

Van De Grift, D. S. (2021). *An Investigation of Seasonal Microbiome Changes in Raw Milk from Organic and Conventional Dairy Farms in Oregon*. Oregon State University. https://ir.library.oregonstate.edu/concern/graduate_thesis_or_dissertations/3484zq262

Verdier-Metz, I., Gagne, G., Bornes, S., Monsallier, F., Veisseire, P., Delbès-Paus, C., & Montel, M.-C. (2012). Cow Teat Skin, a Potential Source of Diverse Microbial Populations for Cheese Production. *Applied and Environmental Microbiology*, *78*(2), 326–333. https://doi.org/10.1128/AEM.06229-11

Vlková, E., Trojanová, I., & Rada, V. (2006). Distribution of bifidobacteria in the gastrointestinal tract of calves. *Folia Microbiologica*, *51*(4), 325–328. https://doi.org/10.1007/BF02931825

Wang, L., Wu, D., Zhang, Y., Li, K., Wang, M., & Ma, J. (2023). Dynamic distribution of gut microbiota in cattle at different breeds and health states. *Frontiers in Microbiology*, *14*. https://doi.org/10.3389/fmicb.2023.1113730

Wang, Y., Nan, X., Zhao, Y., Jiang, L., Wang, M., Wang, H., Zhang, F., Xue, F., Hua, D., Liu, J., Yao, J., & Xiong, B. (2021). Rumen microbiome structure and metabolites activity in dairy cows with clinical and subclinical mastitis. *Journal of Animal Science and Biotechnology*, *12*(1), 36. https://doi.org/10.1186/s40104-020-00543-1

Watanabe, K., Makino, H., Sasamoto, M., Kudo, Y., Fujimoto, J., & Demberel, S. (2009). Bifidobacterium mongoliense sp. Nov., from airag, a traditional fermented mare's milk product from Mongolia. *International Journal of Systematic and Evolutionary Microbiology*, *59*(6), 1535–1540. https://doi.org/10.1099/ijs.0.006247-0

Webb, E. M., Holman, D. B., Schmidt, K. N., Crouse, M. S., Dahlen, C. R., Cushman, R. A., Snider, A. P., McCarthy, K. L., & Amat, S. (2023). A Longitudinal Characterization of the Seminal Microbiota and Antibiotic Resistance in Yearling Beef Bulls Subjected to Different Rates of Gain. *Microbiology Spectrum*, *11*(2), e05180-22. https://doi.org/10.1128/spectrum.05180-22

Wege Dias, N. (2023). *Changes in vaginal microbiome of beef cows enrolled in estrous synchronization protocols and its relation to fertility*. https://vtechworks.lib.vt.edu/handle/10919/113248

Winders, T. M., Holman, D. B., Schmidt, K. N., Luecke, S. M., Smith, D. J., Neville, B. W., Dahlen, C. R., Swanson, K. C., & Amat, S. (2023). Feeding hempseed cake alters the

bovine gut, respiratory and reproductive microbiota. *Scientific Reports*, *13*(1), Article 1. https://doi.org/10.1038/s41598-023-35241-1

Witzke, M. C. (2020). *The Effects of Heat Stress and Feed Restriction on the Microbiome of Lactating Dairy Cows* [M.S., University of Missouri - Columbia]. https://www.proquest.com/docview/2595963849/abstract/38432B6C30BE42D9PQ/1

Wu, Y., Jiao, C., Diao, Q., & Tu, Y. (2023). Effect of Dietary and Age Changes on Ruminal Microbial Diversity in Holstein Calves. *Microorganisms*, *12*(1), 12. https://doi.org/10.3390/microorganisms12010012

Yagisawa, T., Uchiyama, J., Takemura-Uchiyama, I., Ando, S., Ichii, O., Murakami, H., Matsushita, O., & Katagiri, S. (2023). Metataxonomic Analysis of the Uterine Microbiota Associated with Low Fertility in Dairy Cows Using Endometrial Tissues Prior to First Artificial Insemination. *Microbiology Spectrum*, *11*(3), e04764-22. https://doi.org/10.1128/spectrum.04764-22

Yang, L., Bajinka, O., Jarju, P. O., Tan, Y., Taal, A. M., & Ozdemir, G. (2021). The varying effects of antibiotics on gut microbiota. *AMB Express*, *11*(1), 116. https://doi.org/10.1186/s13568-021-01274-w

Yang, L., Huang, W., Yang, C., Ma, T., Hou, Q., Sun, Z., & Zhang, H. (2021). Using PacBio sequencing to investigate the effects of treatment with lactic acid bacteria or antibiotics on cow endometritis. *Electronic Journal of Biotechnology*, *51*, 67–78. https://doi.org/10.1016/j.ejbt.2021.02.004

Zang, X.-W., Sun, H.-Z., Xue, M.-Y., Zhang, Z., Plastow, G., Yang, T., Guan, L. L., & Liu, J.-X. (2022). Heritable and Nonheritable Rumen Bacteria Are Associated with Different Characters of Lactation Performance of Dairy Cows. *mSystems*, *7*(5), e00422-22. https://doi.org/10.1128/msystems.00422-22

Zhang, K., Teng, Z., Meng, Q., Liu, S., Yuan, L., Fu, T., Zhang, N., & Gao, T. (2024). Dynamics of Fermentation Parameters and Bacterial Community in Rumen of Calves During Dietary Protein Oscillation. *Microorganisms*, *12*(11), Article 11. https://doi.org/10.3390/microorganisms12112123

Zhang, S. S., Xu, Z. S., Qin, L. H., & Kong, J. (2020). Low-sugar yogurt making by the co-cultivation of Lactobacillus plantarum WCFS1 with yogurt starter cultures. *Journal of Dairy Science*, *103*(4), 3045–3054. https://doi.org/10.3168/jds.2019-17347

Zhu, H., Miao, R., Tao, X., Wu, J., Liu, L., Qu, J., Liu, H., Sun, Y., Li, L., & Qu, Y. (2023). Longitudinal Changes in Milk Microorganisms in the First Two Months of Lactation of Primiparous and Multiparous Cows. *Animals*, *13*(12), Article 12. https://doi.org/10.3390/ani13121923

Ziemer, C. J. (2014). Newly Cultured Bacteria with Broad Diversity Isolated from Eight-Week Continuous Culture Enrichments of Cow Feces on Complex Polysaccharides. *Applied and Environmental Microbiology*, *80*(2), 574–585. https://doi.org/10.1128/AEM.03016-13

Zou, B., Long, F., Xue, F., Qu, M., Chen, C., Zhang, X., & Xu, L. (2022). Alleviation effects of niacin supplementation on beef cattle subjected to heat stress: A metagenomic insight. *Frontiers in Microbiology*, *13*. https://www.frontiersin.org/articles/10.3389/fmicb.2022.975346
