# Supplementary material for: Gut microbiomes of tribal communities in India vary with dairy and grain consumption
Source: Gut Microbes. 2026 Jul 9;18(1):2694242. doi: 10.1080/19490976.2026.2694242 (PMC13353789; doi:10.1080/19490976.2026.2694242)
Supplement: Supplementary Materials excluding Figures.zip [file KGMI_A_2694242_SM8684.zip › Supplementary Materials excluding Figures/File S1 - 24 hr food intake questionnaire.pdf]

Tribal Microbiome Study

24 Hours Food Intake

24 Hours Dietary assessment

Time	Food item consumed	Ingredients of food item	Quantity
**Morning**	Tea/ coffee/<br>(with/without milk)		
	Milk		
	If others, plz specify		
**Breakfast**	Bread/biscuit/butter		
	Roti/chapati/poori/thalipith		
	Idli/dosa/wada		
	Pohe /upit/sheera		
	Sweets		
	If others, plz specify		
**Lunch**	Roti/paratha/phulka/poori		

	Rice		
	Dal/curry/lentil soup		
	Vegetables		
	Curd/ buttermilk/milk		
	Raw salad/foods		
	If others, plz specify		
**Afternoon**	Tea/ coffee/		
	sharbat/juice		
	Snack /outside snack		
	If others, plz specify		
**Dinner**	Roti/paratha/phulka/poori		
	Rice		

	Dal/curry/usual/besan		
	Vegetable		
	Curd/ buttermilk/milk		
	Raw salad/foods		
	Fruit		
**OFF time**	Alcohol/beer/ Aerated drink		
	Any other fermented beverage		
