# Supplementary material for: Gut microbiomes of tribal communities in India vary with dairy and grain consumption
Source: Gut Microbes. 2026 Jul 9;18(1):2694242. doi: 10.1080/19490976.2026.2694242 (PMC13353789; doi:10.1080/19490976.2026.2694242)
Supplement: Supplementary Captions [file KGMI_A_2694242_SM8229.docx]

## Supplementary Captions

[**Table S1.**](https://docs.google.com/spreadsheets/d/1Xi6QXYL1z_HBjOwDz7Vfx-HYg_aFvDFWzZsVh8TfcjQ/edit?usp=sharing) **Demographic information of study participants.**

[**Table S2.**](https://docs.google.com/spreadsheets/d/1YD7Vk0Avjf9uOd5tqJIhksWogXY8uTvkZXI4X-lNSus/edit?usp=sharing) **MAGs assembled in this study, including 14 novel species.** MAGs were considered novel species if they shared less than 95% nucleotide identity with any genome in GTDB-tk. Genomes labeled with sample codes TS1, TS5, TS8, TS12, RS1, RS5, RS8, or RS12 were assembled from DNA obtained from cultured fecal samples, while all other genomes were assembled from fecal metagenomics.

[**Table S3.**](https://docs.google.com/document/d/1EgEb9R50pipLpr_jYvCr_QzllOlpJDlqzv8Yn7K_hBE/edit?usp=sharing) **References linking genus-level taxa enriched in the Trans-Himalayan populations to dairy farming, fermentation, or metabolism.**

[**Table S4.**](https://docs.google.com/spreadsheets/d/1tY9RZYs4OmayY02hFjVUhFFnH201mK5knZla6ejPBZM/edit?usp=sharing) ***Bifidobacterium* species detected with metagenomics.** N indicates the number of fecal samples where the species was detected, and Rel_ab indicates the average relative abundance among those samples.

[**Table S5.**](https://docs.google.com/spreadsheets/d/1WSfr-OAMYww62-M4AMRX5PrQVwMHSHHtH9hL9tm-U6A/edit?usp=sharing) ***Bifidobacterium adolescentis* genomes.** 20 MAGs from this study plus 112 previously sequenced *B. adolescentis* MAGs and isolates from NCBI and global metagenomic datasets.

[**Table S6.**](https://docs.google.com/spreadsheets/d/1Gd9OFtgAGN0xWwNTOGbqVZAHLDsJVYREa-vFwQEqEl8/edit?usp=sharing) **Episodic diversifying selection of a GH42 domain in *Bifidobacterium*.** Columns A-G are BUSTED results; columns H-R are SLAC results. Higher ER and LR indicate stronger evidence for selection.

[**Table S7**](https://docs.google.com/spreadsheets/d/1gxQuE8zh4n-rXqHdo0kUYegAWB4rudAu3n5WZQgCIjY/edit?usp=sharing)**. CAZyme substrates derived from** <http://www.cazy.org/> and dbCAN-PUL.

**Figure 1 Supplement 1. Food items consumed by each population at any frequency.** Populations are hierarchically clustered using food frequency data.

**Figure 2 Supplement 1. Sequencing depth and taxon detection. ab,** The number of taxa detected per sample is correlated with sequencing depth. **c,** Taxa detected per sample by sequencing method. The overlap of black and white histograms appear gray. **d,** complete per-sample rarefaction curves from 16S RSV read counts. Samples are colored randomly. **e**, expected per-sample richness of metagenomic species at low depths. Unlike in **e**, species discovery within samples is related to species discovery across samples because assembly of novel SRGs is required for detection.

**Figure 2 Supplement 2. Relative abundance correlations between 16S and metagenomic genera.** The 13 genus-level taxa shown are among the 15 most abundant in the 16S data, excluding two groups without equivalent classifications in metagenomics. Statistics are from linear models for each population.

**Figure 2 Supplement 3. Diversity metrics for 16S and metagenomic sequencing.**  **ad**, Taxa detected per sample by population. *p<0.05, Tukey’s Honest Significant Differences test after ANOVA. **bcef**, PCA of taxonomic beta diversity by population, using eight or four populations. Statistics from PERMANOVA by population.

**Figure 2 Supplement 4. Stacked bar plots of 16S genera in each sample.** The 15 most abundant genera are labeled, with rarer taxa grouped as ‘other.’ Genera formerly known as *Prevotella* are in shades of orange. Genera formerly known as *Bacteroides* are in shades of purple. Genera in shades of red are significantly more abundant in the Trans-Himalayan populations than populations from at least one other region (q<0.05). Arrows indicate subjects classified as outliers.

**Figure 2 Supplement 5. Stacked bar plots of metagenomic genera in each sample.** The 15 most abundant genera are labeled, with rarer taxa grouped as ‘other.’ Genera formerly known as *Prevotella* are in shades of orange. Genera formerly known as *Bacteroides* are in shades of purple. Genera in shades of red are significantly more abundant in the Trans-Himalayan populations than populations from at least one other region (q<0.05). Arrows indicate subjects classified as outliers.

**Figure 2 Supplement 6. Manual reclassification of *Prevotella* genomes**. UHGG genomes and MAGs from this study classified as *Prevotella* by GTDB-tk were compared to type genomes of genera recently defined by Hitch et al., 2022 using GToTree.

**Figure 3 Supplement 1. 16S relative abundance of VANISH taxa by population.** VANISH stands for Volatile and/or Associated Negatively with Industrialized Societies of Humans. Published 16S data from samples of two urban Indian populations are also shown: 80 individuals from the city of Ahmedabad in Gujarat, India (Tandon et al., 2018) and 36 individuals from the San Francisco metro area in California, USA (Wastyk et al., 2021).

**Figure 4 Supplement 1. Top 50 16S taxa significantly associated with region.** Blue/red cells indicate higher/lower abundance in the Trans-Himalayan region, which was used as a reference. Gray cells indicate no significant difference. Plot generated by Maaslin2.

**Figure 4 Supplement 2. Multiple abundant taxa distinguish Trans-Himalayan gut microbiomes. a,** Relative abundance of *Segatella*, *Bifidobacterium*, and *Ligilactobacillus* by region via 16S (top) and metagenomics (bottom). **b,** Scatterplots comparing mean relative abundance of *Segatella* to *Bifidobacterium* by population via 16S (top) and metagenomics (bottom). Statistics from linear models. **c,** Stacked bar plot of the mean relative abundance per population of the 15 most abundant genera detected by metagenomics. Rarer taxa are grouped as ‘other.’

**Figure 5 Supplement 1. Additional gut functional pathways related to lactose, galactose, and glucose metabolism. a,** Mean relative abundance of Metacyc and GO functional pathways for lactose, galactose, and glucose catabolism quantified with HUMAnN 2.0. Asterisks represent results of Student’s t-tests. **b-d,** Contribution of metagenomic genera to the pathways from **a.** ***p<0.001, **p<0.01, *p<0.05.

**Figure 6 Supplement 1. Phylogeny of *Bifidobacterium adolescentis* genomes from gut microbiomes across the world.** Tree constructed by RAxML from 104,289 SNVs across 133 genomes. The last character of each tip label indicates whether the genome is a MAG (M) or Isolate (I). Colors correspond to the same countries as in **Figure 6A.**

**Figure 6 Supplement 2. Phylogenetic tree of *Bifidobacterium* species found in the human gut.** Tree was constructed from 74 conserved bacterial loci using GToTree on reference genomes downloaded from NCBI.
