# Supplementary figures and images for: Gut microbiomes of tribal communities in India vary with dairy and grain consumption
Source: Gut Microbes. 2026 Jul 9;18(1):2694242. doi: 10.1080/19490976.2026.2694242 (PMC13353789; doi:10.1080/19490976.2026.2694242)

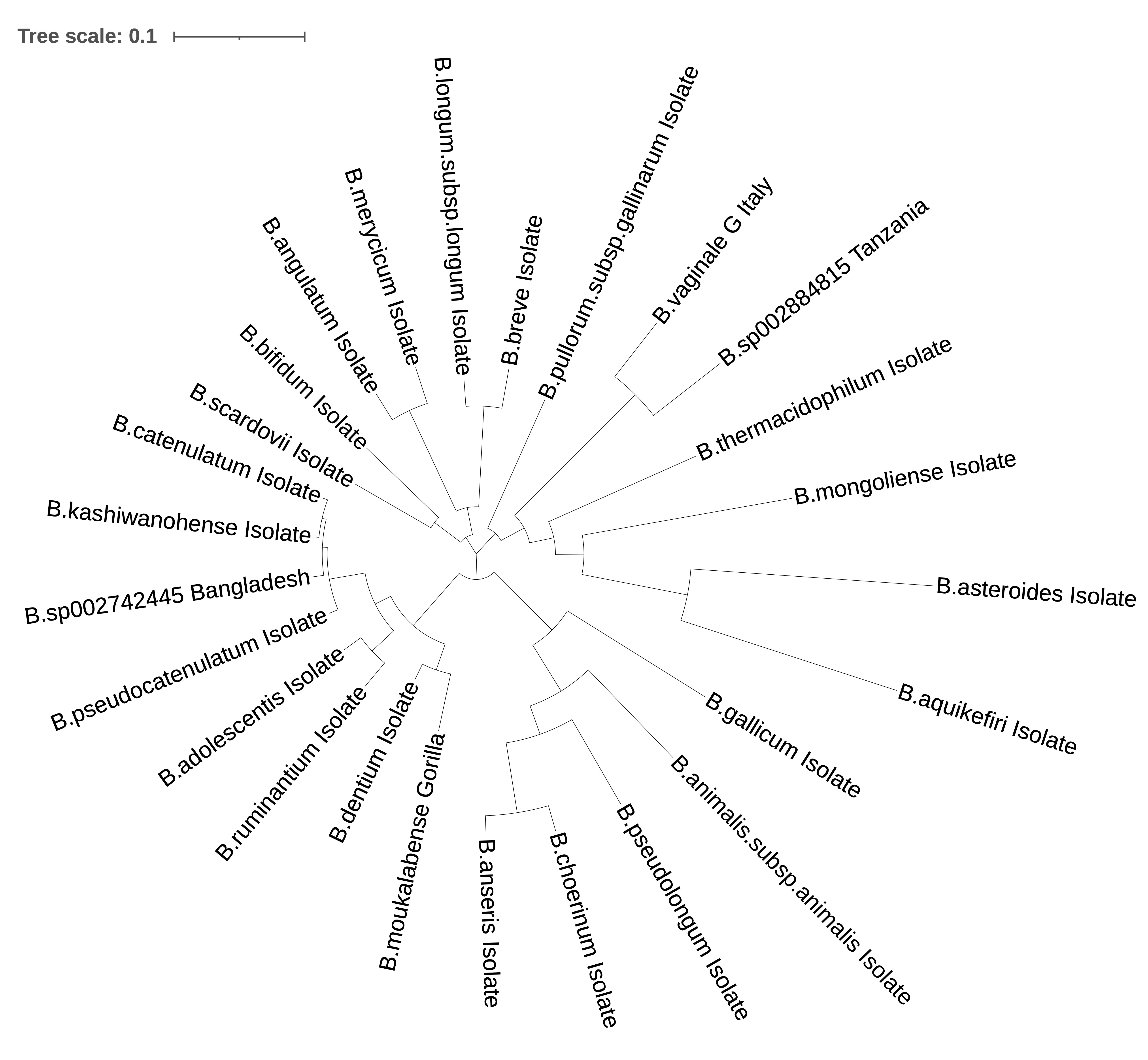

Supplement: Supp Files.zip [file KGMI_A_2694242_SM8685.zip › SFig6-2of2.jpg]

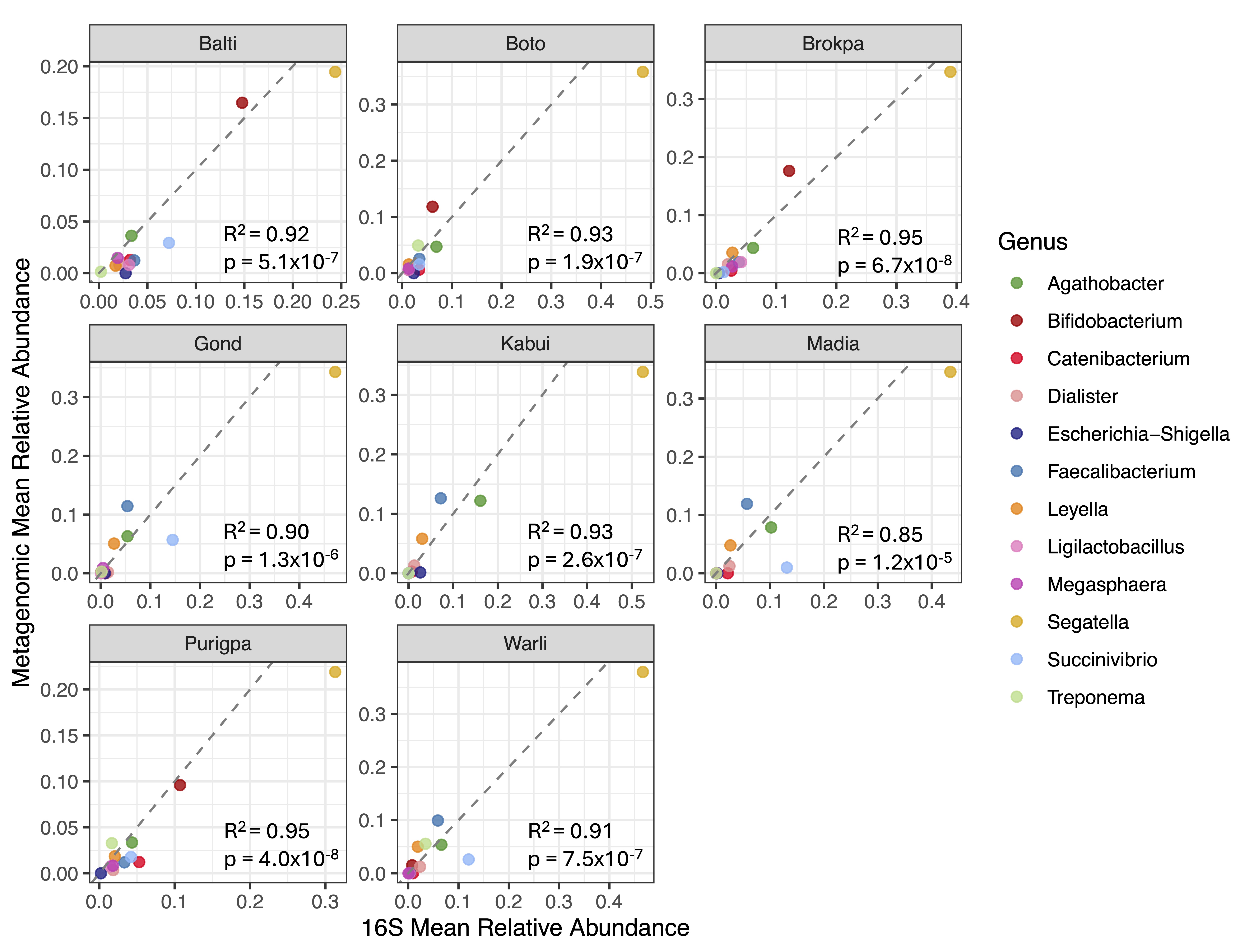

Supplement: Supp Files.zip [file KGMI_A_2694242_SM8685.zip › SFig2-2of6.jpg]

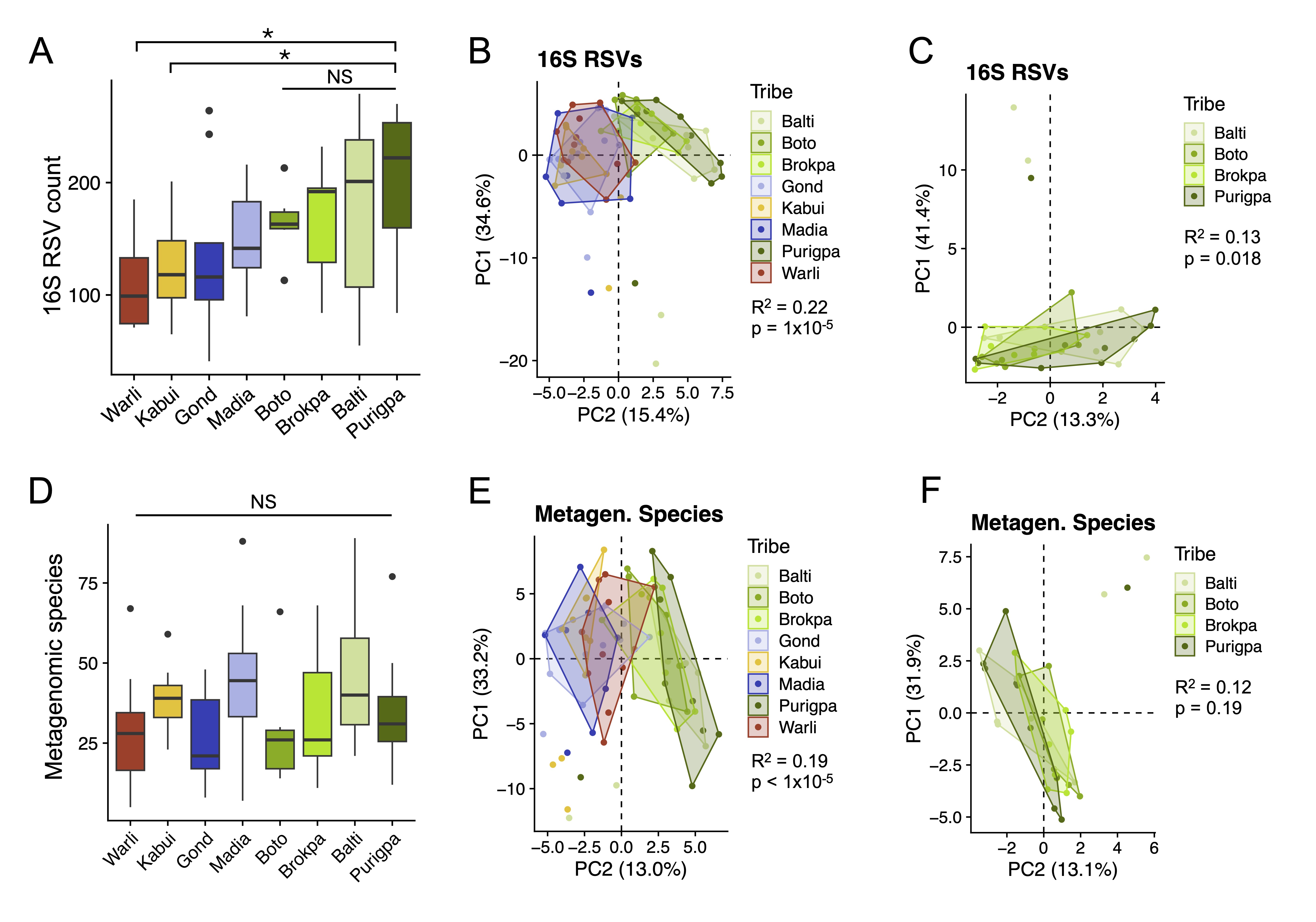

Supplement: Supp Files.zip [file KGMI_A_2694242_SM8685.zip › SFig2-3of6.jpg]

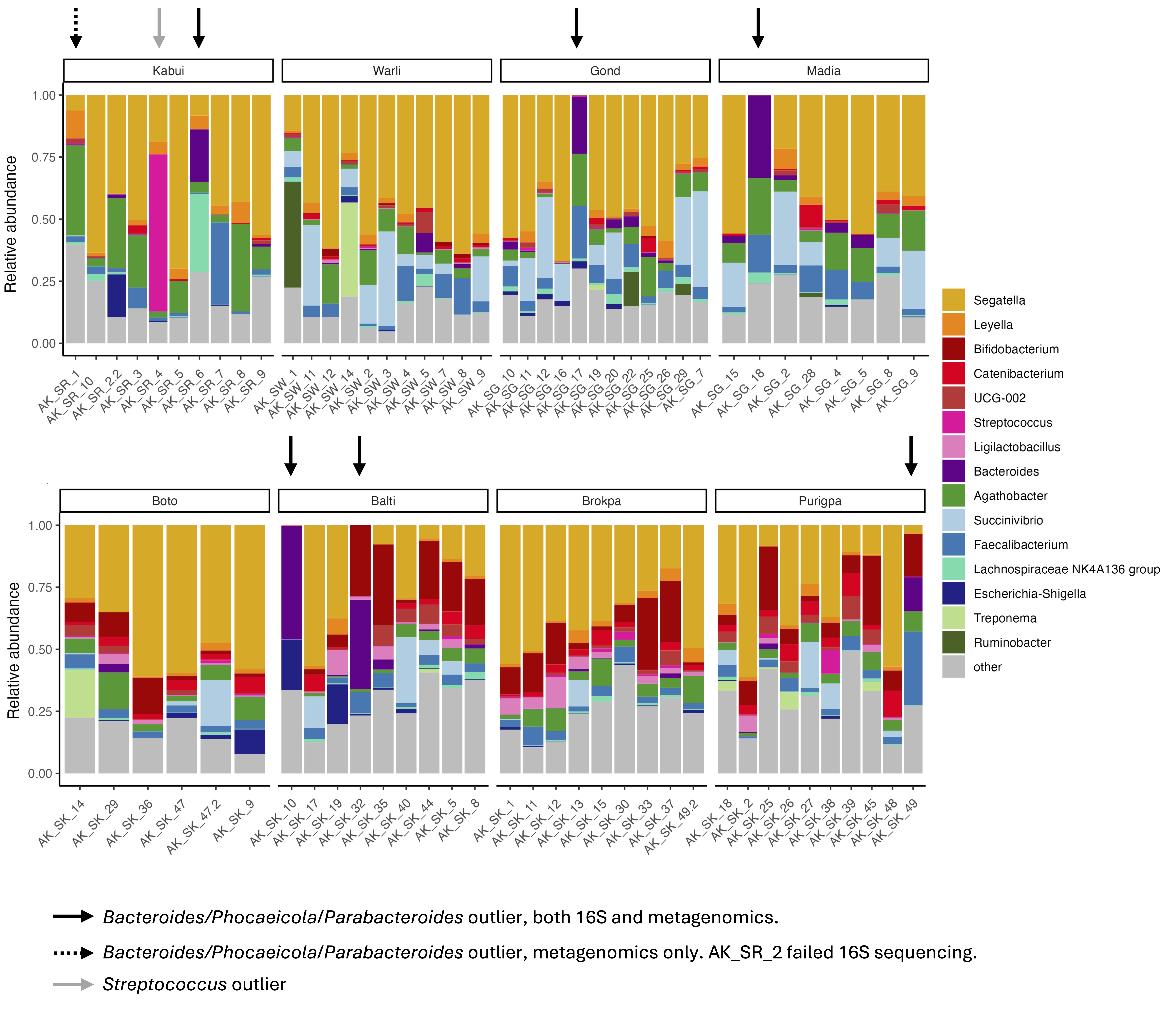

Supplement: Supp Files.zip [file KGMI_A_2694242_SM8685.zip › SFig2-4of6.jpg]

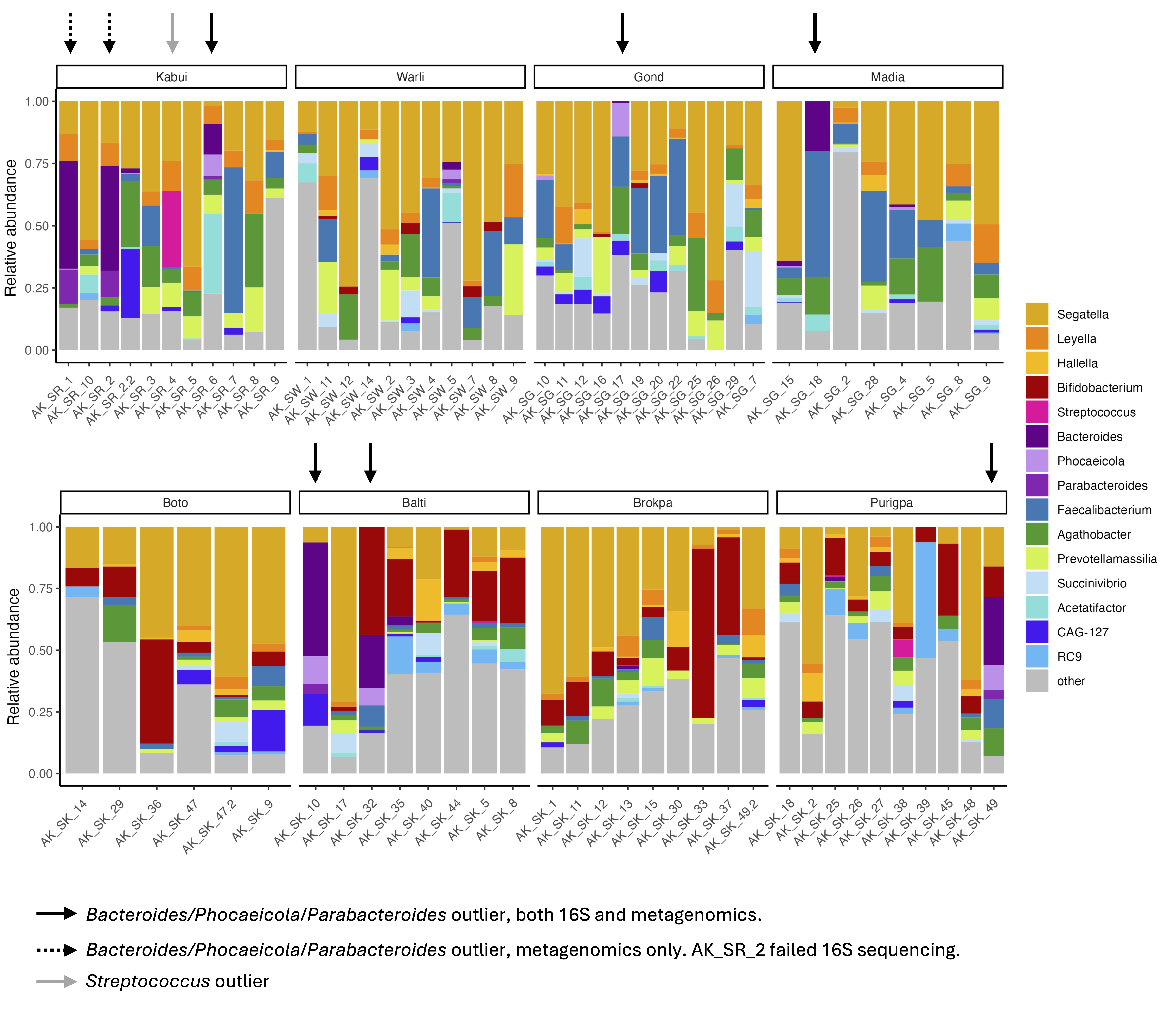

Supplement: Supp Files.zip [file KGMI_A_2694242_SM8685.zip › SFig2-5of6.jpg]

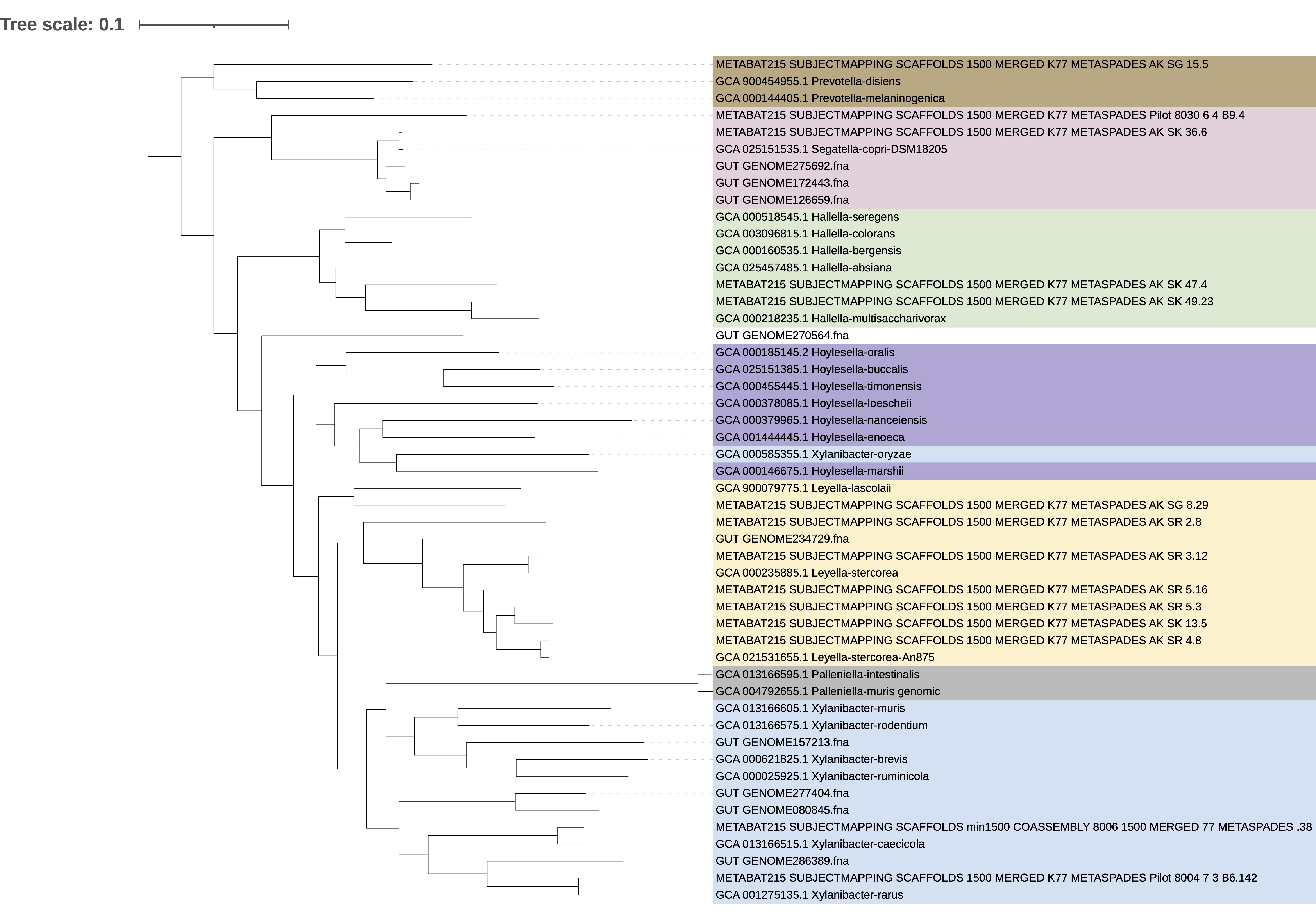

Supplement: Supp Files.zip [file KGMI_A_2694242_SM8685.zip › SFig2-6of6.jpg]

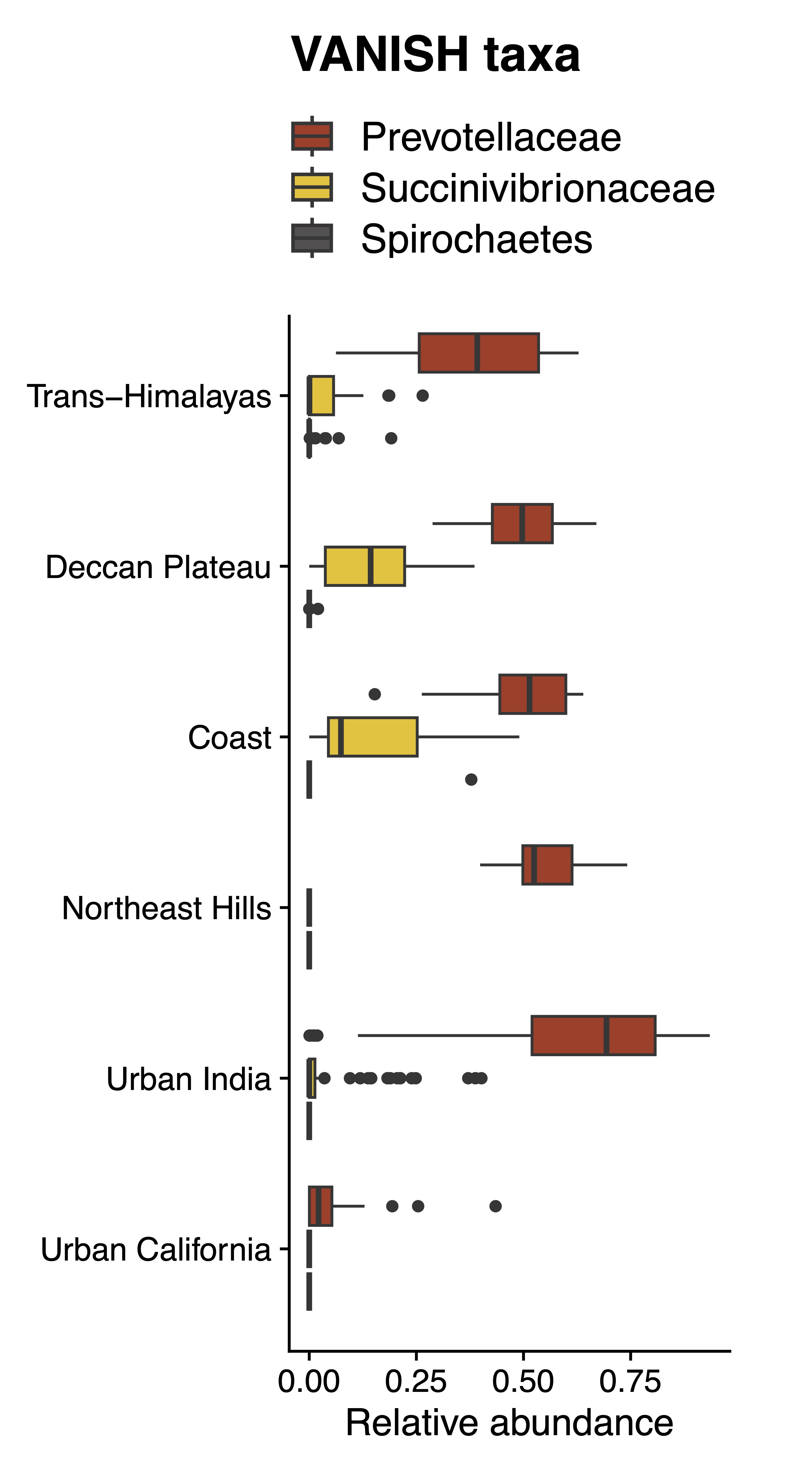

Supplement: Supp Files.zip [file KGMI_A_2694242_SM8685.zip › SFig3-1of1.jpg]

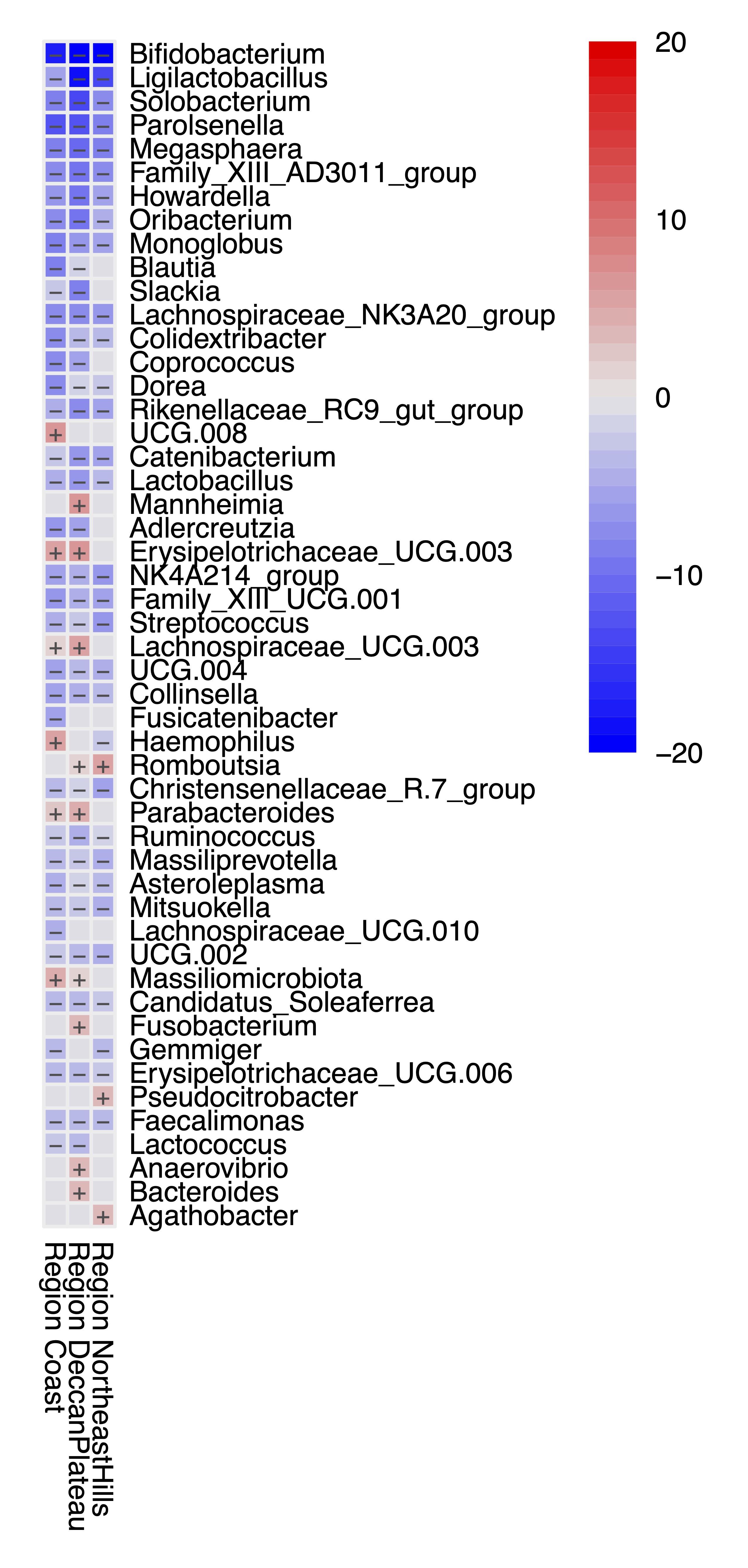

Supplement: Supp Files.zip [file KGMI_A_2694242_SM8685.zip › SFig4-1of2.jpg]

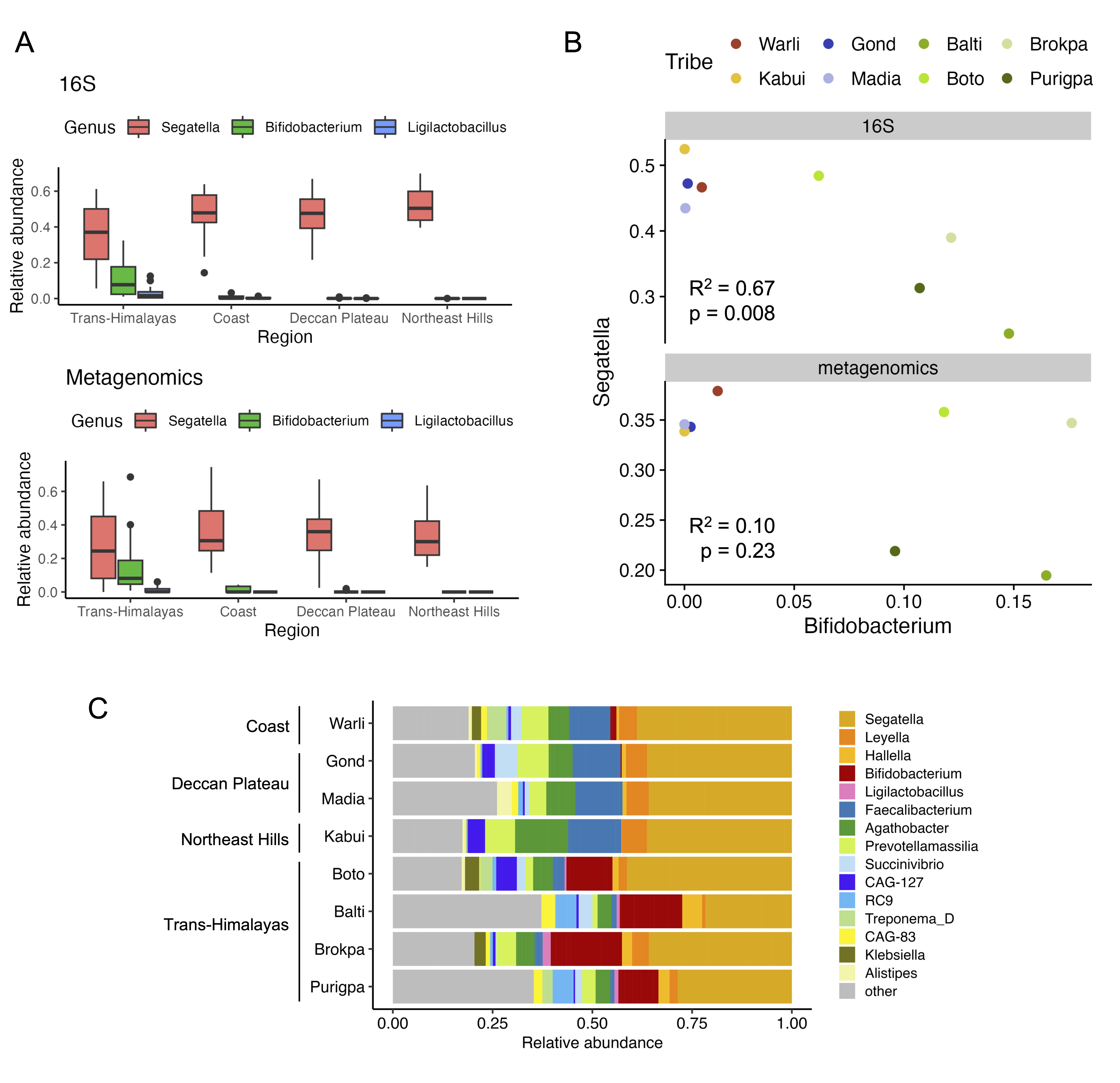

Supplement: Supp Files.zip [file KGMI_A_2694242_SM8685.zip › SFig4-2of2.jpg]

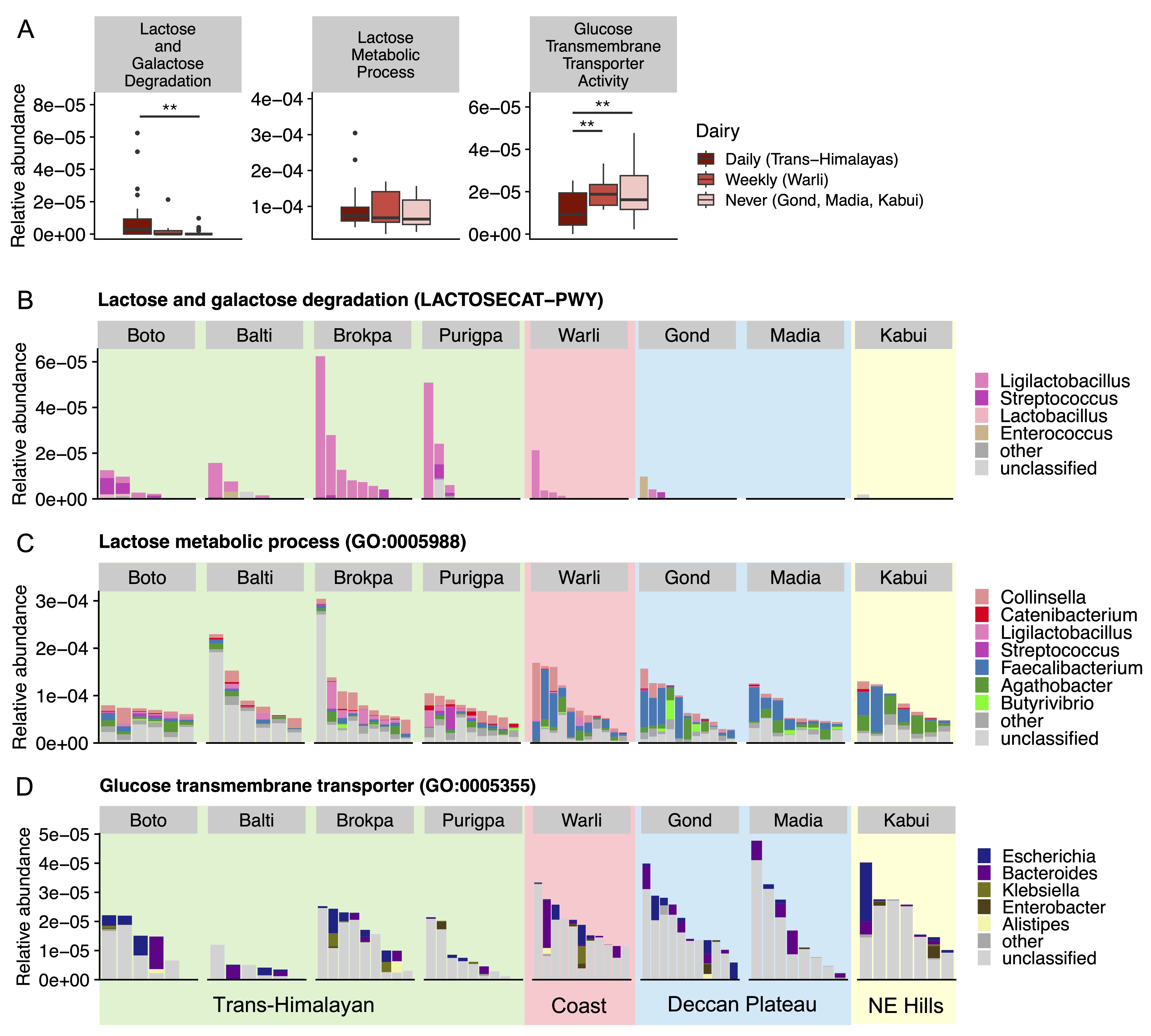

Supplement: Supp Files.zip [file KGMI_A_2694242_SM8685.zip › SFig5-1of1.jpg]

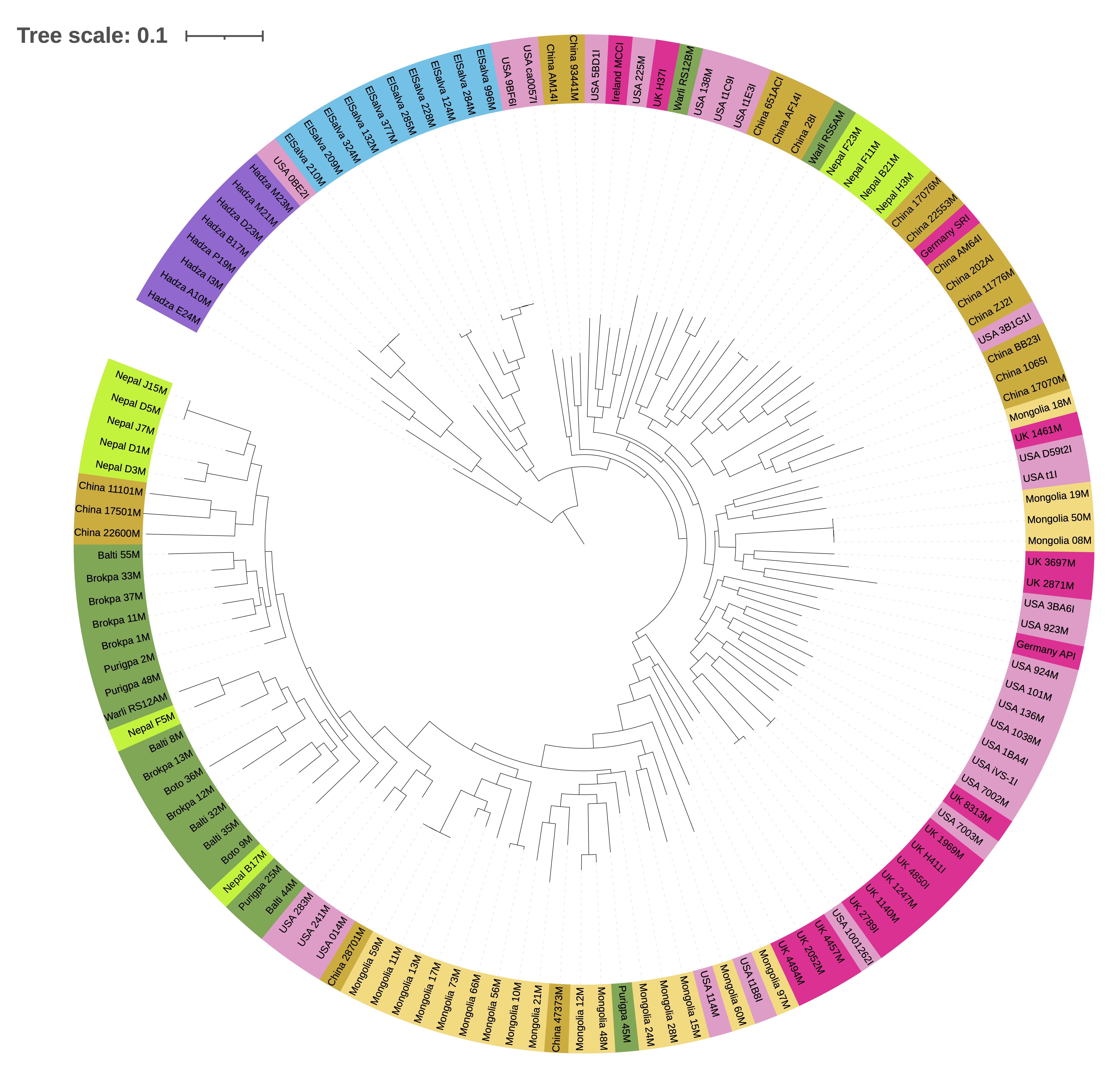

Supplement: Supp Files.zip [file KGMI_A_2694242_SM8685.zip › SFig6-1of2.jpg]

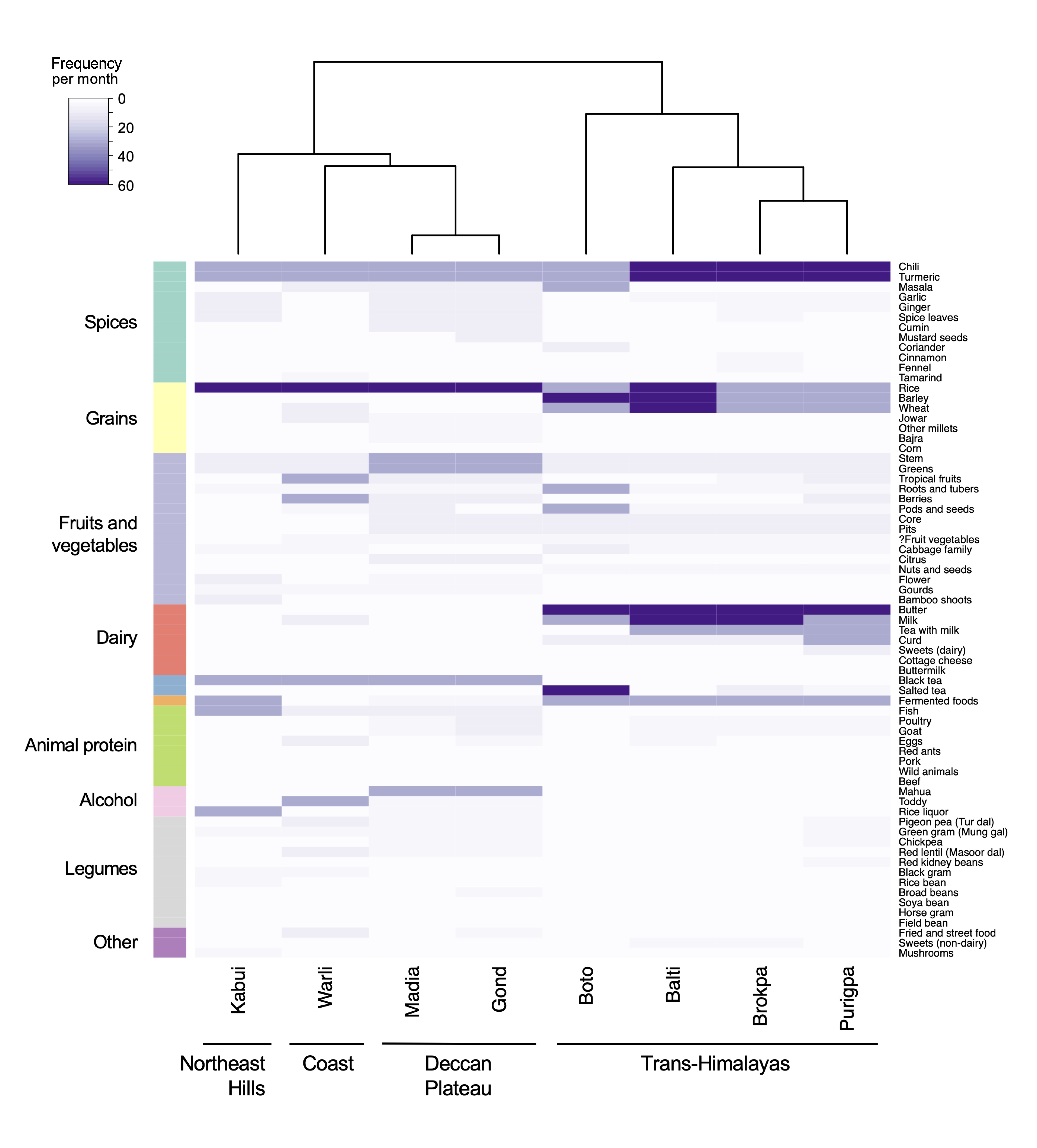

Supplement: Supp Files.zip [file KGMI_A_2694242_SM8685.zip › SFig1-1of1.jpg]

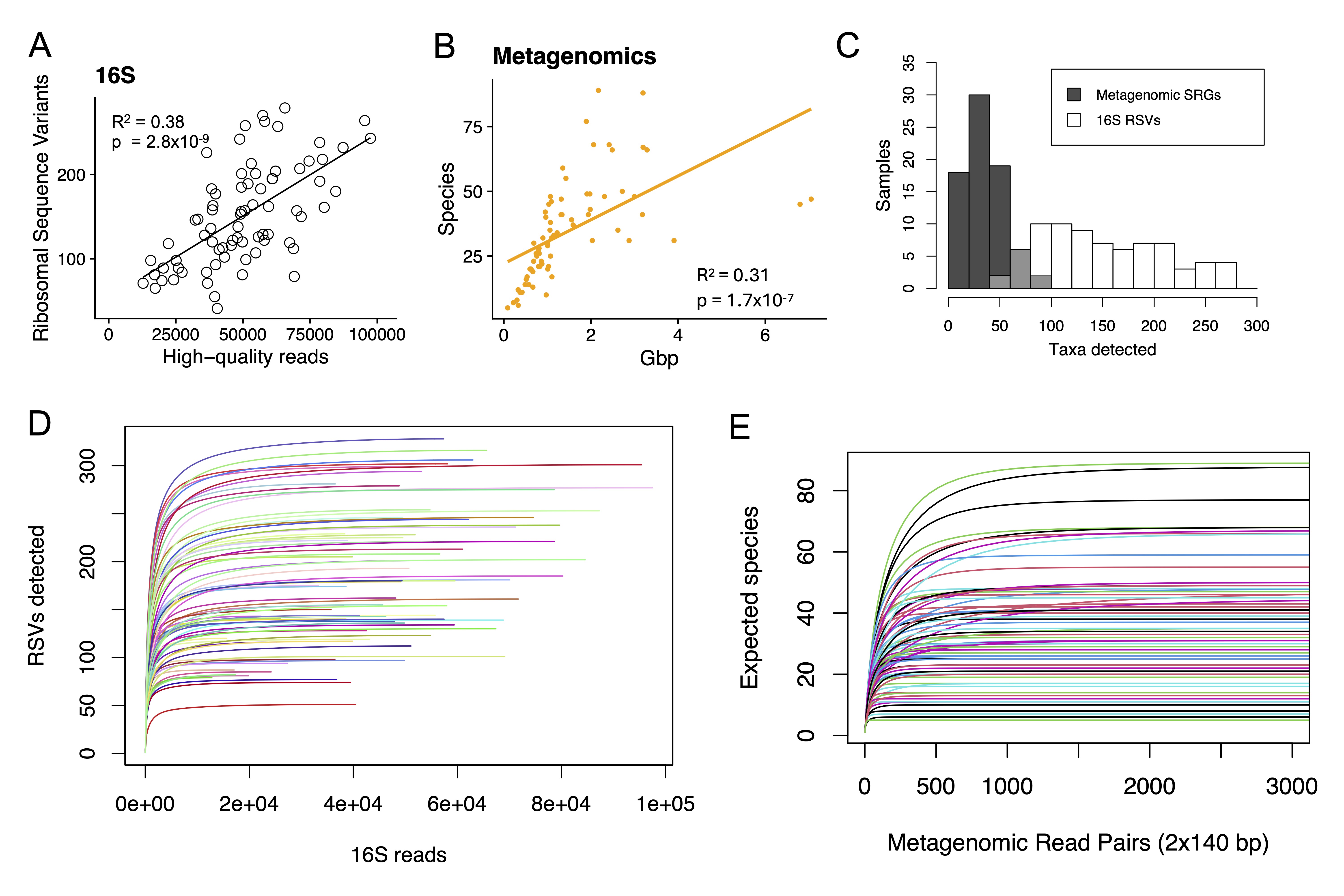

Supplement: Supp Files.zip [file KGMI_A_2694242_SM8685.zip › SFig2-1of6.jpg]
